# Sleep disturbance and psychiatric disorders: a bidirectional Mendelian randomisation study

**DOI:** 10.1017/S2045796021000810

**Published:** 2022-04-25

**Authors:** Xiaohui Sun, Bin Liu, Sitong Liu, David J. H. Wu, Jianming Wang, Yi Qian, Ding Ye, Yingying Mao

**Affiliations:** 1 Department of Epidemiology and Biostatistics, School of Public Health, Zhejiang Chinese Medical University, Hangzhou 310053, China; 2 Department of Internal Medicine, University of Minnesota-Twin Cities Medical School, Minneapolis, MN, USA; 3 Department of Radiation Oncology, Rutgers Cancer Institute of New Jersey, Robert Wood Johnson Medical School, Rutgers, the State University of New Jersey, New Brunswick, NJ 08901, USA

**Keywords:** Epidemiology, evidence-based psychiatry, factor analysis, psychosis, sleep

## Abstract

**Aims:**

Sleep disturbance is an important factor in the pathophysiology and progression of psychiatric disorders, but whether it is a cause, or a downstream effect is still not clear.

**Methods:**

To investigate causal relationships between three sleep-associated traits and seven psychiatric diseases, we used genetic variants related to insomnia, chronotype and sleep duration to perform a two-sample bidirectional Mendelian randomisation analysis. Summary-level data on psychiatric disorders were extracted from the Psychiatric Genomics Consortium. Effect estimates were obtained by using the inverse-variance-weighted (IVW), weights modified IVW, weighted-median methods, MR-Egger regression, MR pleiotropy residual sum and outlier (MR-PRESSO) test and Robust Adjusted Profile Score (RAPS).

**Results:**

The causal odds ratio (OR) estimate of genetically determined insomnia was 1.33 (95% confidence interval (CI) 1.22–1.45; *p* = 5.03 × 10^−11^) for attention-deficit/hyperactivity disorder (ADHD), 1.31 (95% CI 1.25–1.37; *p* = 6.88 × 10^−31^) for major depressive disorder (MDD) and 1.32 (95% CI 1.23–1.40; *p* = 1.42 × 10^−16^) for post-traumatic stress disorder (PTSD). There were suggestive inverse associations of morningness chronotype with risk of MDD and schizophrenia (SCZ). Genetically predicted sleep duration was also nominally associated with the risk of bipolar disorder (BD). Conversely, PTSD and MDD were associated with an increased risk of insomnia (OR = 1.06, 95% CI 1.03–1.10, *p* = 7.85 × 10^−4^ for PTSD; OR = 1.37, 95% CI 1.14–1.64; *p* = 0.001 for MDD). A suggestive inverse association of ADHD and MDD with sleep duration was also observed.

**Conclusions:**

Our findings provide evidence of potential causal relationships between sleep disturbance and psychiatric disorders. This suggests that abnormal sleep patterns may serve as markers for psychiatric disorders and offer opportunities for prevention and management in psychiatric disorders.

## Introduction

Psychiatric disorders have become a leading public health concern with high morbidity and mortality (Demyttenaere *et al*., 2004). It is estimated that the global burden of psychiatric illness contributes to nearly 21.2–32.4% of years lived with disability (Gurillo *et al*., 2015). By now, psychiatric illness management is still challenging in clinical settings, which usually addresses the symptoms of the disease without targeting the underlying cause (Rush *et al*., 2006). Therefore, a better understanding of the pathophysiology and potential risk factors of psychiatric disorders is urgently needed for developing novel prevention and intervention strategies.

To date, the aetiology of psychiatric disorders remains unclear. Emerging evidence suggests that the causes of psychiatric illness are multifactorial, involving environmental and genetic exposures, as well as parental psychopathologic conditions (Paananen *et al*., 2021). Recently, increasing interest has been devoted to exploring the relationships between sleep disturbances and psychiatric disorders. Abnormal sleep patterns, such as evening chronotype, shorter sleep duration and insomnia, were found to be disproportionately higher in patients with psychiatric illness. For example, previous observational studies indicated that evening chronotype was associated with increased risks of anxiety disorders (Antypa *et al*., 2016), major depressive disorder (MDD) (Bahk *et al*., 2014), bipolar disorder (BD) (Haraden *et al*., 2017), as well as schizophrenia (SCZ) (Chiu *et al*., 2017). However, other studies failed to provide evidence to support such relationships (Zanini *et al*., 2015; Chiu *et al*., 2017). Several cross-sectional studies have instead observed a U-shaped association between sleep duration and post-traumatic stress disorder (PTSD), MDD and BD (Xiang *et al*., 2009; Park *et al*., 2010; Geoffroy *et al*., 2020). Another study also reported that a polygenic risk score accounting for 1.4% of the phenotypic variance in sleep duration was associated with the risk of depression (Dashti *et al*., 2019). For insomnia, some cohort studies have suggested positive associations of insomnia with attention-deficit/hyperactivity disorder (ADHD) (Liu *et al*., 2020), BD (Lundervold *et al*., 2020), PTSD (Neylan, 2013), MDD (Ohayon and Roth, 2003), obsessive-compulsive disorder (OCD) (Alvaro *et al*., 2017) and SCZ (Chung *et al*., 2018). These associations between sleep-associated traits and psychiatric disorders may be to some extent affected by confounding or selection biases inherent in conventional observational studies. Moreover, whether such sleep disturbance is a cause, or a downstream effect of psychiatric disorders is still controversial. Thus, any potential underlying cause-effect relationships still require further investigation.

In such cases, Mendelian randomisation (MR), a genetic epidemiological method is a useful tool for evaluating the causal role of sleep disturbances in psychiatric illness. By using genetic variants such as single nucleotide polymorphisms (SNPs) as instrumental variables (IVs) for modifiable disease risk factors or exposures, MR design can strengthen the causal inference on an exposure-outcome association. According to Mendel's Law of Inheritance, because genetic variants are randomly allocated in the process of gamete formation, they are therefore less susceptible to confounding factors. In addition, since genotype cannot be changed by the development of the disease, confounding factors and reverse causation can be minimized. To clarify whether sleep disturbance is associated with psychiatric disorders and to evaluate the directionality of such associations, we performed a two-sample bidirectional MR study by using the most up-to-date genome-wide association studies (GWASs). As these sleep-related traits can be a target for direct pharmacological treatment and lifestyle alterations, elucidating the impacts of these traits on the risk of psychiatric disorders may provide useful improvements for clinical monitoring and precision medicine in the future.

## Method

### Study design

**Fig. 1.**
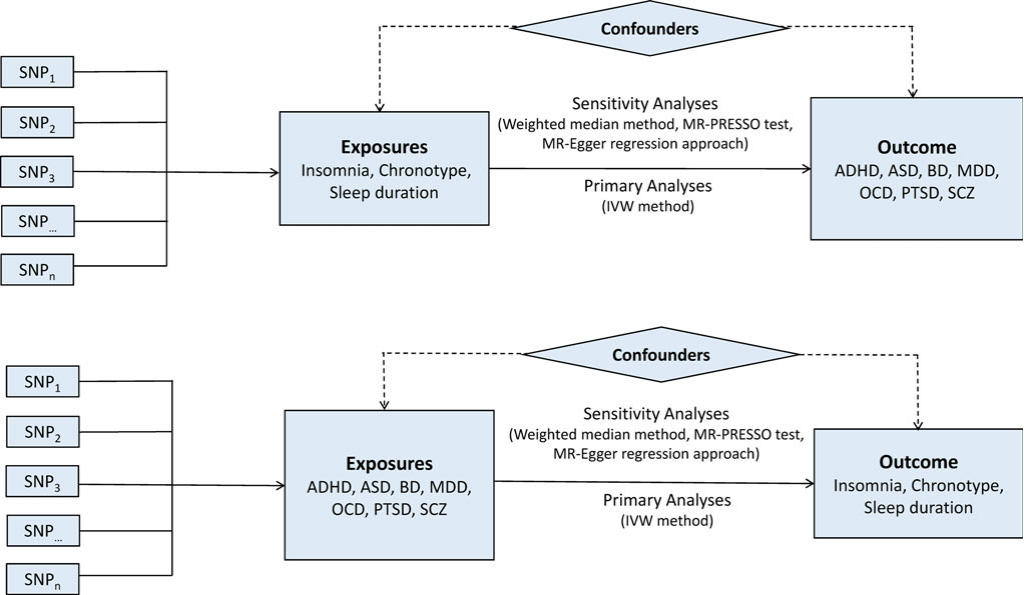
Bidirectional Mendelian randomisation study workflow. ADHD, attention-deficit/hyperactivity disorder; ASD, autism spectrum disorder; BD, bipolar disorder; IVW, inverse variance weighted method; MDD, major depressive disorder; MR-PRESSO, Mendelian randomisation pleiotropy residual sum and outlier test; OCD, obsessive-compulsive disorder; PTSD, post-traumatic stress disorder; SCZ, schizophrenia; SNP, single nucleotide polymorphism.

A two-sample bidirectional MR design was used to evaluate the potential causal effects of sleep-associated traits on the risk of psychiatric disorders, including ADHD, autism spectrum disorder (ASD), BD, MDD, OCD, PTSD and SCZ (Fig. 1). The assumptions of MR design include: (1) the genetic variants used as IVs are related to exposures of interest; (2) IVs are not related to the confounders; (3) IVs affect the risk of outcome only via the exposure of interest (Lawlor, 2016). In this study, the SNPs that achieved genome-wide significance (*p* < 5 × 10^−8^) for chronotype, sleep duration and insomnia symptoms were used as IVs (Dashti *et al*., 2019; Jansen *et al*., 2019; Jones *et al*., 2019). Data on the genetic associations of these SNPs with psychiatric disorders were obtained from the Psychiatric Genomics (PGC) Consortium. This study was based on publicly available summarised data and the approval for each research can be found in the corresponding original studies. More information regarding the GWAS datasets employed in this study is displayed in online Supplementary Table 1.

### Genetic associations with sleep-associated traits

Genetic predictors for three sleep-related traits, chronotype, sleep duration and insomnia, were extracted from the most up-to-date GWASs. For chronotype, we used data from a GWAS meta-analysis with 697 828 individuals (Jones *et al*., 2019). In this study, the morning chronotype was evaluated via self-reported assessment. Each participating GWAS was adjusted for the corresponding population structure and individual relatedness. Genetic instruments for sleep duration were extracted from the most recent GWAS, which included up to 446 118 participants (Dashti *et al*., 2019). Sleep duration was determined by self-reported information, and it was treated as a continuous variable. A recent GWAS meta-analysis performed by Jansen *et al*., was used to identify genetic variants associated with insomnia symptoms (Jansen *et al*., 2019). This study included a total of 1 331 010 individuals from two contributing studies (UK Biobank and 23andMe Inc.).

Summary statistics for three sleep-related traits were obtained from the UK Biobank from which well-powered GWAS data was available. The UK Biobank is a long-term prospective cohort of more than 500 000 participants, with collections of anthropometric, health, and lifestyle data, as well as biological samples (Collins, 2012). Of 487 409 individuals genotyped in the UK Biobank, we obtained summary data from 449 734 participants with chronotype measures, 446 118 with sleep duration and 386 533 with insomnia (online Supplementary Table 1). More detailed information on the UK Biobank has been described elsewhere (Bycroft *et al*., 2018; Jones *et al*., 2019).

### Genetic associations with psychiatric disorders

The genetic instruments for psychiatric disorders were derived from the largest GWAS or GWAS meta-analysis (online Supplementary Table 1). Briefly, all individuals included in these GWASs were of European ethnic origin, except for ADHD, in which 96.5% of participants were European. The SNPs which achieved *p* < 5 × 10^−8^ were selected as IVs. Since Zhu *et al*., recommended that the application of at least 10 independent SNPs with exposure-associated as IVs can maintain the study power in MR analysis (Zhu *et al*., 2018), we relaxed the GWAS *p*-value threshold to 5 × 10^−6^ in ADHD, ASD, OCD and PTSD to allow for a sufficient number of SNPs to be included in the MR analysis.

Summary statistics for seven psychiatric disorders were derived from the PGC Consortium. To reduce the potential bias of population heterogeneity, genetic data from the European population in accordance with the exposure data were used. The respective sample sizes were as follows: ADHD (19 099 cases and 34 194 controls), ASD (14 759 cases and 155 327 controls), BD (20 352 cases and 31 358 controls), MDD (135 458 cases and 344 901 control), OCD (2688 cases and 7037 controls), PTSD (23 212 cases and 151 447 controls) and SCZ (33 640 cases and 43 456 controls). Due to partial sample overlap in the data source for sleep-related traits and the largest GWASs for MDD and PTSD, we also used summary-level data from previous smaller GWASs for sensitivity analysis (Major Depressive Disorder Working Group of the Psychiatric *et al*., 2013; Duncan *et al*., 2018). Detailed information of data source is displayed in online Supplementary Table 1.

### Selection of genetic instruments

Independent SNPs (*r*^2^ < 0.01 and distance > 250 kb) associated with predicted exposures were selected. To meet the independence assumption of MR analysis, SNPs which have been reported to be associated with secondary traits by searching the GWAS Catalog (http://www.ebi.ac.uk/gwas, accessed 8 October 2020) were removed (online Supplementary Tables 2 and 3). After exclusion of potential pleiotropic SNPs, 228 SNPs for chronotype, 69 SNPs for sleep duration and 150 SNPs for insomnia were used as IVs (online Supplementary Table 4). For psychiatric disorders, 55 SNPs for ADHD, 9 SNP for ASD, 18 SNPs for BD, 12 SNPs for MDD, 13 SNPs for OCD, 21 SNPs for PTSD and 85 SNPs for SCZ were selected. Detailed information of these SNPs is displayed in online Supplementary Table 5. The SNPs associated with the exposure were extracted from each outcome, and where SNPs for the exposure were not available, they were further removed. The number of genetic variants included in the final MR analysis are displayed in online Supplementary Tables 6 and 7.

### Statistical analysis

For MR analysis, the random-effects inverse-variance weighted (IVW) method was applied to evaluate the association of genetically predicted sleep-associated traits with the risk of psychiatric disorders as the main analysis. This method obtained effect estimates of each SNP on exposure and risk of outcome to calculate the Wald estimates. Though this method can provide precise estimates, it is susceptible to the influences of invalid IVs and potential pleiotropic effects. Therefore, we carried out several sensitivity analyses to assess the robustness of the association. First, we estimated associations using the weighted-median method in which we assumed that at least 50% of weights were derived from the valid instruments. Second, MR-Egger regression was employed to identify the presence of directional pleiotropy by testing whether the intercept was statistically different from zero. Third, the MR pleiotropy residual sum and outlier (MR-PRESSO) test was used to detect possible outliers and obtain the corrected results via outlier removal. Fourth, weights modified IVW method was used which modified second order weighs within the radial regression based on the IVW meta-analysis (Bowden *et al*., 2019). This method can provide a higher statistical power if the key assumptions of the MR analyses are met. Finally, we performed a recently proposed MR method named Robust Adjusted Profile Score (RAPS). A broad consistency in the results of these methods suggests a robust validity of the causal effect estimates.

The associations between genetically predicted sleep-related traits and risk of psychiatric disorders were presented as odds ratios (ORs) with their 95% confidence intervals (CIs). All analyses were performed using R (version 3.6.0) with ‘MendelianRandomization’, ‘TwoSampleMR’, ‘MR-PRESSO’, ‘mr.raps’ and ‘RadialMR’ packages. An observed *p* < 0.0024 (0.05/21) was considered as statistically significant evidence for a causal association. A *p*-value < 0.05, but above the Bonferroni-corrected threshold, was considered suggestive evidence for a potential causal association.

## Results

### Genetically predicted sleep-associated traits on psychiatric disorders

For IVs used for sleep-associated traits, all the *F*-statistics were above 10, ranging from 26.9 to 220.8. The median *F*-statistic was 53.7 for chronotype, 34.7 for sleep duration and 37.3 for insomnia, suggesting weak instrument bias was unlikely.

**Fig. 2.**
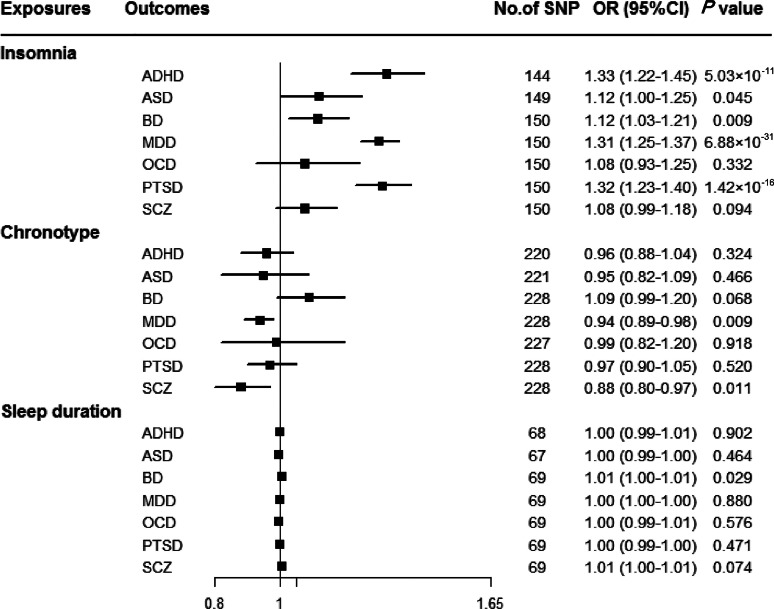
Associations between genetically predicted sleep-associated traits and risk of psychiatric disorders. ADHD, attention-deficit/hyperactivity disorder; ASD, autism spectrum disorder; BD, bipolar disorder; CI, confidence interval; MDD, major depressive disorder; OR, odds ratio; OCD, obsessive-compulsive disorder; PTSD, post-traumatic stress disorder; SCZ, schizophrenia; SNP, single nucleotide polymorphism.

Genetically predicted insomnia was associated with higher odds of ADHD (OR = 1.33, 95% CI 1.22–1.45; *p* = 5.03 × 10^−11^), MDD (OR = 1.31, 95% CI 1.25–1.37; *p* = 6.88 × 10^−31^) and PTSD (OR = 1.32, 95% CI 1.23–1.40; *p* = 1.42 × 10^−16^) in the main analyses (Fig. 2). Results were consistent across sensitivity analyses albeit with larger CIs in weighted-median approaches (online Supplementary Table 8). None of the MR-Egger intercepts significantly deviated from zero, with *p*-values of 0.359 for ADHD, 0.851 for MDD and 0.840 for PTSD (online Supplementary Table 8). By using the MR-PRESSO test, three outlier SNPs of ADHD, and two outliers of MDD were detected. After correcting for possible outliers, the significance and magnitude of all these associations persisted (OR = 1.35, 95% CI 1.24–1.45; *p* = 9.23 × 10^−12^ for ADHD; OR = 1.30, 95% CI 1.25–1.36; *p* = 8.10 × 10^−24^ for MDD; OR = 1.31, 95% CI 1.23–1.40; *p* = 7.16 × 10^−14^ for PTSD) (online Supplementary Table 8). The weights modified IVW and RAPS yielded similar estimates (online Supplementary Table 8). Furthermore, the association between insomnia and risk of PTSD was consistent when using a smaller outcome dataset which did not include data from the UK Biobank (OR = 1.30, 95% CI 1.08–1.56). For MDD, the OR was 1.15 (95% CI 0.97–1.36) when using the smaller GWAS dataset (online Supplementary Table 9).

In addition, we observed nominal associations between genetically predicted insomnia and higher odds of ASD (OR = 1.12, 95% CI 1.00–1.25; *p* = 0.045) and BD (OR = 1.12, 95% CI 1.03–1.21; *p* = 0.009) (online Supplementary Table 8). The weighted-median method produced a similar effect estimate (OR = 1.08, 95% CI 0.92–1.26, *p* = 0.337 for ASD; OR = 1.12, 95% CI 1.01–1.23, *p* = 0.025 for BD). There is no evidence of horizontal pleiotropic bias by using MR-Egger regression (*p* for intercept = 0.061 for ASD; *p* for intercept = 0.297 for BD). Similar associations were also observed in MR-PRESSO test, weights modified IVW and RAPS methods (online Supplementary Table 8). However, no statistically significant effects of insomnia symptoms on risk of OCD (OR = 1.08, 95% CI 0.93–1.25; *p* = 0.332) or SCZ (OR = 1.08, 95% CI 0.99–1.18; *p* = 0.094) were observed (Fig. 2).

There were suggestive inverse associations between genetically predicted morningness chronotype and risk of MDD (OR = 0.94, 95% CI 0.89–0.98; *p* = 0.009) and SCZ (OR = 0.88, 95% CI 0.80–0.97; *p* = 0.011) using the IVW method (Fig. 2). Sensitivity analyses provided consistent results with the main analysis (online Supplementary Table 10). Nevertheless, no statistically significant associations between genetically determined chronotype and risk of ADHD, ASD, BD, OCD, or PTSD were observed.

For sleep duration, the IVW method yielded a nominal association of sleep duration on BD (OR = 1.01, 95% CI 1.00–1.01; *p* = 0.029) (Fig. 2). Sensitivity analysis of weighted-median gained a similar result (OR = 1.01, 95% CI 1.00–1.01; *p* = 0.026) (online Supplementary Table 11). Results of MR-Egger regression did not suggest evidence of pleiotropy (*p* for intercept = 0.545). Additionally, we did not observe statistically significant effects of sleep duration on other psychiatric disorders was observed (Fig. 2).

### Genetically predicted psychiatric disorders on sleep-associated traits

Reserve MR analyses were performed to investigate the potential causal effects of psychiatric disorders on sleep-associated traits. The median *F*-statistic was 25.11 for ADHD, 23.48 for ASD, 32.3 for BD, 35.7 for MDD, 22.75 for OCD, 23.72 for PTSD and 36.0 for SCZ, indicating that weak instrument bias was not severe.

**Fig. 3.**
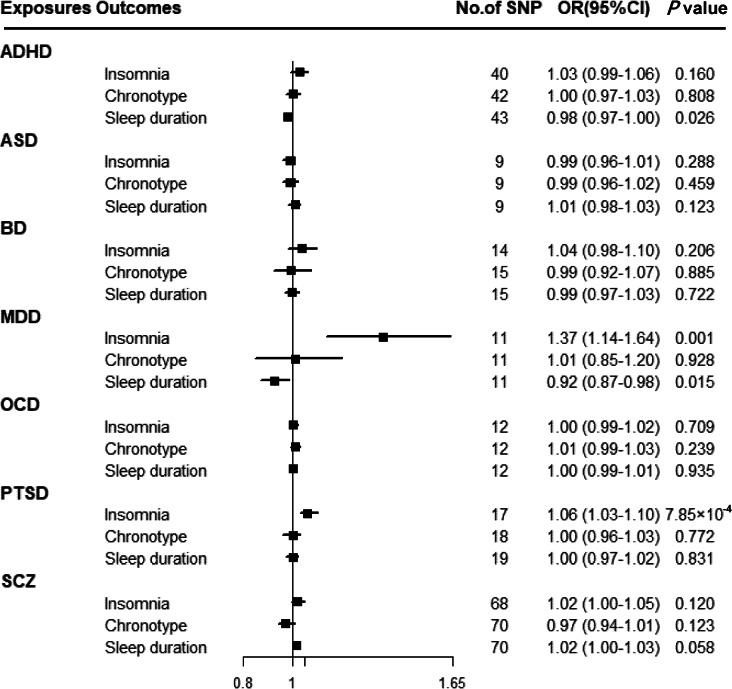
Associations between genetically predicted psychiatric disorders and sleep-associated traits. ADHD, attention-deficit/hyperactivity disorder; ASD, autism spectrum disorder; BD, bipolar disorder; CI, confidence interval; MDD, major depressive disorder; OCD, obsessive-compulsive disorder; OR, odds ratio; PTSD, post-traumatic stress disorder; SCZ, schizophrenia; SNP, single nucleotide polymorphism.

Figure 3 shows that there was a statistically significant causal effect of MDD on insomnia after adjusting for multiple testing. The corresponding effect estimate was 1.37 (95% CI 1.14–1.64; *p* = 0.001) for MDD in the IVW method and remained consistent in the weighted-median method (OR = 1.40, 95% CI 1.18–1.65; *p* = 1.24 × 10^−4^) (online Supplementary Table 12). MR-Egger intercept did not identify any pleiotropic SNPs (*p* for intercept = 0.067). After eliminating the outlier SNP, we found the effect between MDD and insomnia was still statistically significant by using MR-PRESSO test (OR = 1.45, 95% CI 1.24–1.69; *p* = 0.001). Moreover, genetically predicted PTSD showed an adverse effect on insomnia (OR = 1.06, 95% CI 1.03–1.10; *p* = 7.85 × 10^−4^) with no evidence of directional pleiotropy (*p* for intercept = 0.603) (online Supplementary Table 12). The findings from sensitivity analyses were consistent.

Suggestive evidence was observed between ADHD and sleep duration with an OR of 0.98 (95% CI 0.97–1.00; *p* = 0.026) in the IVW method (online Supplementary Table 12). MR-Egger intercepts showed no evidence for significant horizontal pleiotropy (*p* for intercept = 0.989). However, such a relationship disappeared in the weighted-median method. Moreover, a suggestive association between MDD and sleep duration was found using the IVW method (OR = 0.92, 95% CI 0.87–0.98; *p* = 0.015), and consistent results were observed in sensitivity analyses (online Supplementary Table 12).

## Discussion

In the present study, we performed a bidirectional MR analysis to evaluate the associations between three sleep-associated traits and risk of psychiatric diseases. Our findings indicated that genetically predicted insomnia was associated with an increased risk of ADSD, MDD and PTSD. There was also suggestive evidence for a possible causal effect of chronotype on MDD and SCZ, as well as sleep duration on BD. Conversely in the other direction, MDD and PTSD may be causally associated with insomnia.

Growing evidence from epidemiological studies indicated that sleep-related traits are associated with overall or specific psychiatric disorders. Several observational studies suggested a one-way relationship, where sleep disturbance predicts psychiatric illness (Ohayon and Roth, 2003). For example, a meta-analysis of thirteen cohort studies indicated that insomnia was associated with an increased risk of depression, anxiety and psychosis (Hertenstein *et al*., 2019). Other longitudinal studies also reported that insomnia was strongly associated with future onset of ADHD (Liu *et al*., 2020), BD (Lundervold *et al*., 2020), PTSD (Neylan, 2013), MDD (Neylan, 2013), OCD (Alvaro *et al*., 2017) and SCZ (Chung *et al*., 2015). For chronotype, several cross-sectional studies suggested positive correlations of evening chronotype with MDD, BD, PTSD and SCZ (Kitamura *et al*., 2010; Levandovski *et al*., 2011). There are limited observational data on the association of sleep duration with the risk of psychiatric disorders. A cross-sectional study with 6510 Korean participants highlighted a U-shaped association between sleep duration and psychiatric disorders (Bycroft *et al*., 2018). Our findings corroborated with results from some, but not all, observational studies. Under the MR assumption, we found genetically predicted insomnia was associated with an increased risk of ADHD, ASD, BD, MDD and SCZ, which was partially consistent with a previous MR study (Gao *et al*., 2019). Findings from study of Gao *et al*., indicated a potential causal effect of insomnia on the risk of ASD (OR = 1.74, 95% CI 1.22–2.49) and BD (OR = 1.79, 95% CI 1.40–2.29), but no effects were found for ADHD, MDD or SCZ. In present study, since the GWASs with a larger sample size were utilised, we obtained consistent results with the previously established association of insomnia with ASD and BD, in addition to finding new potential causal associations for ADHD, MDD and SCZ.

Psychiatric illnesses have also been reported to lead to sleep disturbance. For example, a cross-sectional study found ADHD was associated with both short and long sleep duration (Wynchank *et al*., 2018). Besides, a cohort study with 2619 individuals showed that short sleep duration persisted even after remittance of MDD (van Mill *et al*., 2010). Consistent with these results, we found suggestive evidence for these relationships. Moreover, we found that PTSD and MDD may exert a potential causal effect on insomnia, suggesting a bidirectional causal association between them. Consistently, it is reported that approximately 30–60% of individuals with PTSD meet the criteria for comorbid insomnia (Colvonen *et al*., 2018). Findings from other studies also suggested that insomnia plays a vital role in the development of PTSD (Gehrman *et al*., 2013; Koffel *et al*., 2013). In terms of MDD, a cohort study involving 3134 individuals suggested that MDD could increase the risk of subsequent insomnia by 2–3 fold, and meanwhile, insomnia increased the subsequent risk of MDD (Roberts and Duong, 2013). Another cohort study in Switzerland also supported this reciprocal effect (Buysse *et al*., 2008).

For underlying mechanisms, the shared common abnormalities in neurobiology, including hypothalamic–pituitary–adrenal (HPA) axis activation, serotonin system dysfunction, as well as overexpression of immune system peptides may interpret it (Gao *et al*., 2019). Additionally, genetic mutations in the circadian ‘clock gene’ were found to be related with multiple psychiatric disorders (Landgraf *et al*., 2014). Moreover, the presence of a biological clock allows the organism to anticipate and adapt to environmental alterations by controlling circadian rhythms and orchestrating the expression of appropriate downstream genes (Tordjman *et al*., 2015). An erratic sleep pattern may alter the functioning of biological, emotional and social rhythms, consequently leading to social impairments and vulnerability to psychiatric disorders (Charrier *et al*., 2017). The relationships of sleep-associated traits with different psychiatric disorders identified in the present study indicate that though they share similarities, there also exist differences in biological pathways and etiopathogenic mechanisms. However, further studies are required to clarify such mechanisms.

## Strengths and limitations

A major strength of the present study was the MR study design, which minimises residual confounding and reverse causality inherent in observational studies and allows us to interrogate the potential causality between sleep disturbance and psychiatric disorders. The consistency across sensitivity analyses further supports the validity of the effect estimates. Moreover, IVs used in this study were extracted from the most up to date GWAS of sleep-related traits with the largest sample sizes and were strongly associated with the exposure of interest, which minimised weak instrument bias and increased statistical power.

However, there are some limitations in this study. First, genetic instruments of ADHD in the reverse MR were derived from GWASs of trans-ancestry populations, which may cause bias because of the population mixture. The same genetic variant may exhibit different pleiotropic effects in different ethnic groups. However, bias resulting from this would be minimal because 96.5% of participants in the ADHD GWAS were of European ancestry, and previous studies demonstrated the genetic architecture for common diseases was likely similar across ethnic populations. Second, MR analyses performed on a dichotomisation of an underlying continuous factor (e.g. chronotype, sleep duration) may be biased by horizontal pleiotropy, since the pleiotropic effects from within-category variation cannot be fully identified by MR approaches (Taylor *et al*., 2014). Third, the associations identified in this study were the cumulative effects of exposure over an individual's lifetime. Additional studies could look to evaluate sleep-associated traits at various points in the life span instead, to uncover the possible effects of changes in sleep on the risk of psychiatric disorders. Moreover, since a small fraction of phenotypic variance was explained in some traits, it might lead to lower statistical power and therefore less precise estimates. Finally, as we were unable to investigate the potential nonlinear effect of sleep-associated traits on psychiatric disorders due to the assumption of summary-data level MR analyses, further studies are warranted to verify the nature of these relationships.

## Conclusions

The findings of this study provided evidence that insomnia was associated with an increased risk of ADHD, MDD and PTSD. There was suggestive evidence for a possible causal effect of chronotype on MDD and SCZ, as well as sleep duration on BD. Conversely, PTSD and MDD may be causally associated with insomnia, as well as ADHD and MDD with sleep duration. Further research is warranted to understand the biological pathways underpinning these associations to help clinicians and researchers develop novel prophylactic and interventional approaches for psychiatric disorders.

## Data Availability

Data on sleep traits was provided by Jones *et al*. (2019), Dashti *et al*. (2019) and Jansen *et al*. (2019). Data on psychiatric disorders was provided by the PGC Consortium investigators and can be downloaded from https://www.med.unc.edu/pgc/.

## Supplementary material

For supplementary material accompanying this paper visit https://doi.org/10.1017/S2045796021000810. click here to view supplementary material
