# Supplementary material for: Sleep disturbance and psychiatric disorders: a bidirectional Mendelian randomisation study
Source: Epidemiol Psychiatr Sci. 2022 Apr 25;31:e26. doi: 10.1017/S2045796021000810 (PMC9069588; doi:10.1017/S2045796021000810)
Supplement: Supplementary file 1 [file S2045796021000810sup001.docx]

**Supplementary Materials**

**Supplementary Table 1.** Detailed information regarding studies and datasets used in the present study.

**Supplementary Table 2.** The potential secondary phenotypes of sleep traits-associated SNPs from the GWAS Catalog.

**Supplementary Table 3.** The potential secondary phenotypes of psychiatric disorders-associated SNPs from the GWAS Catalog.

**Supplementary Table 4.** Characteristics of the associations of instrumental variables for three sleep-associated traits.

**Supplementary Table 5.** Characteristics of instrumental variables for psychiatric disorders used in the bidirectional Mendelian randomization.

**Supplementary Table 6.** The number of genetic variants used as instrumental variables for sleep-related traits in the present study.

**Supplementary Table 7.** The number of genetic variants used as instrumental variables psychiatric disorders in present study.

**Supplementary Table 8.** Associations between genetically predicted insomnia and risk of psychiatric disorders.

**Supplementary Table 9.** Mendelian randomization analyses of sleep-associated traits with major depressive disorder and post-traumatic stress disorder using dataset excluding the UK Biobank dataset.

**Supplementary Table 10.** Associations between genetically predicted chronotype and risk of psychiatric disorders.

**Supplementary Table 11.** Associations between genetically predicted sleep duration and risk of risk of psychiatric disorders.

**Supplementary Table 12.** Associations between genetically predicted psychiatric disorders and sleep-associated traits.

| **Supplementary Table1. Detailed information of studies and datasets used in the present study.** | | | | |
| --- | --- | --- | --- | --- |
| Exposure or outcome | PMID | Ancestry | Participants | Web source |
| **Data used in MR** |  |  |  |  |
| Chronotype | 30696823 | European ancestry | 697,828 individuals | / |
| Insomnia | 30804565 | European ancestry | 1,331,010 individuals | / |
| Sleep duration | 30846698 | European ancestry | 446,118 individuals | / |
| Attention-deficit/hyperactivity disorder | 30478444 | European ancestry | 19,099 cases and 34,194 controls | <https://www.med.unc.edu/pgc/download-results/> |
| Autism spectrum disorder | 28540026 | European ancestry | 14,759 cases and 155,327 controls | <https://www.med.unc.edu/pgc/download-results/> |
| Bipolar disorder | 31043756 | European ancestry | 20,352 cases and 31,358 controls | <https://www.med.unc.edu/pgc/download-results/> |
| Major depressive disorder | 29700475 | European ancestry | 135,458 cases and 344,901 controls | <https://www.med.unc.edu/pgc/download-results/> |
| Obsessive-compulsive disorder | 28761083 | European ancestry | 2,688 cases and 7,037 controls | https://www.med.unc.edu/pgc/download-results/ |
| Post-traumatic stress disorder | 31594949 | European ancestry | 23,212 cases and 151,447 controls | <https://www.med.unc.edu/pgc/download-results/> |
| Schizophrenia | 25056061 | European ancestry | 33,640 cases and 43,456 controls | <https://www.med.unc.edu/pgc/download-results/> |
| **Data used in inverse MR** |  |  |  |  |
| Attention-deficit/hyperactivity disorder | 30478444 | European and Asian ancestry | 20,183 cases and 35,191 controls | / |
| Autism spectrum disorder | 28540026 | European ancestry | 14,759 cases and 155,327 controls | / |
| Bipolar disorder | 31043756 | European ancestry | 20,352 cases and 31,358 controls | / |
| Major depressive disorder | 29700475 | European ancestry | 135,458 cases and 344,901 controls | / |
| Post-traumatic stress disorder | 31594949 | European ancestry | 23,212 cases and 151,447 controls | / |
| Schizophrenia | 29483656 | European ancestry | 40,675 cases and 64,643 controls | / |
| Chronotype | 30696823 | European ancestry | 449,734 individuals | <http://www.t2diabetesgenes.org/data/> |
| Insomnia | 30804565 | European ancestry | 386,533 individuals | https://ctg.cncr.nl/software/summary_statistics |
| Sleep duration | 30846698 | European ancestry | 446,118 individuals | <http://sleepdisordergenetics.org/> |
| **Data used in sensitivity analysis excluding UKBB dataset** | | |  |  |
| Major depressive disorder | 22472876 | European ancestry | 16,023 cases and 62,014 controls | https://www.med.unc.edu/pgc/download-results/ |
| Post-traumatic stress disorder | 28439101 | European ancestry | 5,131 cases and 15,092 controls | https://www.med.unc.edu/pgc/download-results/ |

**Supplementary Table 2. The potential secondary phenotypes of sleep traits-associated SNPs from the GWAS Catalog.**

| SNP | Trait(s) | *P*-value | PMID |
| --- | --- | --- | --- |
| **Chronotype** |  |  |  |
| rs12140153 | Body fat percentage | 1.00E-09 | 30593698 |
|  | Sleep traits (multi-trait analysis) | 1.00E-10 | 27992416 |
|  | Body mass index | 8.00E-27 | 30595370 |
|  | C-reactive protein levels | 5.00E-08 | 31900758 |
|  | Waist-hip ratio | 8.00E-10 | 30239722 |
|  | Type 2 diabetes | 1.00E-08 | 30297969 |
| rs72720396 | Smoking initiation | 1.00E-08 | 30617275 |
|  | Smoking initiation (ever regular vs never regular) | 7.00E-10 | 30679032 |
| rs975025 | Schizophrenia | 2.00E-09 | 31740837 |
| rs1064213 | Body mass index | 3.00E-13 | 31669095 |
|  | Allergic disease (asthma | 5.00E-12 | 29083406 |
|  | Insomnia | 6.00E-10 | 30804565 |
|  | Body mass index | 9.00E-14 | 30595370 |
| rs113851554 | Sleep traits (multi-trait analysis) | 4.00E-16 | 27992416 |
|  | Insomnia complaints | 4.00E-19 | 28604731 |
|  | Insomnia symptoms (never/rarely vs. usually) | 1.00E-43 | 30804566 |
|  | Relative amplitude of rest-activity cycles | 6.00E-11 | 30120083 |
|  | Insomnia complaints (dichotomous) | 2.00E-19 | 28604731 |
|  | Sleep duration | 3.00E-18 | 30531941 |
|  | Restless legs syndrome | 2.00E-280 | 29029846 |
|  | Insomnia complaints (continuous) | 6.00E-22 | 28604731 |
|  | Insomnia | 2.00E-51 | 30804565 |
| rs11681299 | Height | 2.00E-68 | 30595370 |
| rs2706762 | Blood protein levels | 1.00E-121 | 30072576 |
|  | Educational attainment (years of education) | 2.00E-09 | 30595370 |
|  | Cognitive performance (MTAG) | 2.00E-09 | 30038396 |
|  | Educational attainment (MTAG) | 3.00E-09 | 30038396 |
|  | Highest math class taken (MTAG) | 1.00E-08 | 30038396 |
| rs6433478 | Accelerometer-based physical activity measurement (fraction of time with accelerations >425 milli-gravities) | 5.00E-09 | 29899525 |
| rs75120545 | Alcohol consumption (drinks per week) | 6.00E-09 | 30679032 |
|  | Smoking status | 3.00E-08 | 30595370 |
|  | Number of sexual partners | 1.00E-11 | 30643258 |
| rs812925 | Immature fraction of reticulocytes | 2.00E-20 | 27863252 |
| rs35346733 | Anorexia nervosa | 8.00E-10 | 31835028 |
|  | Schizophrenia | 2.00E-12 | 29483656 |
| rs42210 | Educational attainment (MTAG) | 2.00E-11 | 30038396 |
|  | Highest math class taken (MTAG) | 4.00E-12 | 30038396 |
|  | Self-reported math ability (MTAG) | 6.00E-09 | 30038396 |
|  | Educational attainment (years of education) | 3.00E-08 | 30595370 |
| rs77960 | Self-reported math ability (MTAG) | 2.00E-11 | 30038396 |
| rs2653349 | Medication use (agents acting on the renin-angiotensin system) | 1.00E-08 | 31015401 |
| rs3923809 | Insomnia | 2.00E-08 | 30804565 |
|  | Restless legs syndrome | 1.00E-17 | 17634447 |
| rs766406 | Height | 2.00E-12 | 28552196 |
|  | Asthma | 6.00E-14 | 32296059 |
|  | Educational attainment (MTAG) | 2.00E-23 | 30038396 |
| rs2072413 | QT interval | 1.00E-49 | 24952745 |
| rs4729854 | Type 2 diabetes | 3.00E-10 | 30595370 |
| rs2737245 | Medication use (HMG CoA reductase inhibitors) | 4.00E-10 | 31015401 |
| rs6468316 | Blood protein levels | 1.00E-08 | 30072576 |
| rs295268 | Eosinophil counts | 1.00E-15 | 30595370 |
| rs1163238 | Birth weight | 8.00E-09 | 31097437 |
| rs2298117 | Triglycerides | 2.00E-09 | 30275531 |
| rs3808964 | Self-reported math ability (MTAG) | 4.00E-10 | 30038396 |
|  | Self-reported math ability | 1.00E-09 | 30038396 |
| rs10832648 | White blood cell count | 4.00E-08 | 30595370 |
| rs10838687 | C-reactive protein levels | 9.00E-13 | 30388399 |
|  | Proinsulin levels | 7.00E-12 | 21873549 |
| rs2433634 | Automobile speeding propensity | 3.00E-09 | 30643258 |
| rs7975791 | Heel bone mineral density | 3.00E-10 | 30598549 |
| rs45597035 | Colorectal cancer | 2.00E-10 | 31089142 |
|  | Colorectal cancer or advanced adenoma | 3.00E-09 | 30510241 |
| rs12927162 | Insomnia | 4.00E-09 | 30804565 |
| rs1421085 | Waist circumference variance | 3.00E-52 | 31453325 |
|  | Childhood body mass index | 5.00E-16 | 26604143 |
|  | Hip circumference | 2.00E-152 | 31453325 |
|  | Basal metabolic rate | 9.00E-154 | 31453325 |
|  | Obesity | 6.00E-39 | 23563607 |
|  | Body fat percentage | 3.00E-50 | 30593698 |
|  | Basal metabolic rate variance | 9.00E-47 | 31453325 |
|  | Body mass index | 1.00E-300 | 30595370 |
|  | Hand grip strength | 5.00E-38 | 29691431 |
|  | Pulse pressure | 2.00E-10 | 30487518 |
|  | Type 2 diabetes | 3.00E-84 | 30297969 |
|  | Alcohol consumption | 9.00E-13 | 31358974 |
|  | Age at adiposity rebound | 3.00E-30 | 31840077 |
|  | Medication use (HMG CoA reductase inhibitors) | 5.00E-15 | 31015401 |
|  | Waist circumference | 3.00E-166 | 31453325 |
|  | Hip circumference variance | 2.00E-48 | 31453325 |
|  | Alcohol use disorder | 2.00E-11 | 30940813 |
|  | Obesity (early onset extreme) | 3.00E-28 | 23563609 |
|  | Dietary macronutrient intake | 1.00E-16 | 29988085 |
|  | Fat-free mass | 4.00E-62 | 30593698 |
| rs17604349 | Educational attainment (years of education) | 2.00E-12 | 30038396 |
| rs58681483 | Monocyte count | 1.00E-12 | 29403010 |
| rs1788784 | Mental health study participation (provided email address) | 1.00E-10 | 31263887 |
| rs9956387 | Height | 4.00E-19 | 30595370 |
| rs9964420 | Insomnia | 5.00E-08 | 30804565 |
| rs56113850 | Smoking behavior (cigarette pack-years) | 6.00E-25 | 30679032 |
|  | Post bronchodilator FEV1 | 2.00E-08 | 26634245 |
|  | Coffee consumption | 8.00E-16 | 31046077 |
|  | Smoking cessation | 2.00E-48 | 30643251 |
|  | Smoking cessation (MTAG) | 6.00E-10 | 30643251 |
|  | Bitter non-alcoholic beverage consumption | 7.00E-11 | 31046077 |
|  | Bitter beverage consumption | 8.00E-11 | 31046077 |
|  | Lung cancer in ever smokers | 4.00E-13 | 28604730 |
|  | Nicotine metabolite ratio in current smokers | 6.00E-261 | 32157176 |
|  | Lung cancer | 5.00E-19 | 28604730 |
|  | Non-small cell lung cancer | 3.00E-09 | 31326317 |
|  | Smoking behavior (cigarettes smoked per day) | 4.00E-99 | 30643251 |
|  | Local histogram emphysema pattern | 1.00E-09 | 25006744 |
|  | Cigarettes smoked per day (MTAG) | 5.00E-58 | 30643251 |
|  | Post bronchodilator FEV1/FVC ratio | 5.00E-09 | 26634245 |
|  | Smoking intensity (cotinine levels/cigarettes per day) | 3.00E-13 | 32157176 |
|  | Lung adenocarcinoma | 9.00E-10 | 28604730 |
|  | Squamous cell lung carcinoma | 7.00E-11 | 28604730 |
| rs2072727 | Sleep duration | 4.00E-08 | 30846698 |
| rs6007594 | Male-pattern baldness | 4.00E-27 | 30573740 |
| rs812925 | Immature fraction of reticulocytes | 2.00E-20 | 61453858 |
| **Insomnia** |  |  |  |
| rs1015438 | Self-reported math ability (MTAG) | 2.00E-11 | 30038396 |
|  | Vertical cup-disc ratio (multi-trait analysis) | 1.00E-09 | 31959993 |
| rs1038093 | Age of smoking initiation (MTAG) | 2.00E-08 | 30643251 |
| rs10502966 | Intelligence (MTAG) | 1.00E-11 | 29326435 |
| rs10947428 | Eczema | 2.00E-10 | 30595370 |
|  | Allergic disease (asthma | 4.00E-13 | 29083406 |
| rs11090039 | Experiencing mood swings | 2.00E-10 | 29500382 |
| rs113851554 | Morning person | 2.00E-13 | 30696823 |
|  | Sleep traits (multi-trait analysis) | 4.00E-16 | 27992416 |
|  | Morningness | 1.00E-08 | 30804565 |
|  | Chronotype | 2.00E-13 | 30696823 |
|  | Relative amplitude of rest-activity cycles | 6.00E-11 | 30120083 |
|  | Sleep duration | 3.00E-18 | 30531941 |
|  | Restless legs syndrome | 2.00E-280 | 29029846 |
| rs12187443 | General risk tolerance (MTAG) | 2.00E-12 | 30643258 |
| rs12251016 | Body mass index | 1.00E-25 | 30239722 |
| rs12520974 | Depressive symptoms | 2.00E-12 | 30643256 |
| rs13010288 | Educational attainment (MTAG) | 1.00E-21 | 30038396 |
|  | Highest math class taken (MTAG) | 2.00E-11 | 30038396 |
| rs13010288 | Educational attainment (years of education) | 3.00E-21 | 30038396 |
| rs13135092 | Alcohol use disorder (total score) | 3.00E-14 | 30336701 |
|  | Hand grip strength | 3.00E-26 | 29691431 |
|  | White blood cell count | 4.00E-09 | 30595370 |
|  | Adventurousness | 3.00E-11 | 30643258 |
|  | Total cholesterol levels | 5.00E-11 | 29507422 |
|  | HDL cholesterol | 3.00E-13 | 25961943 |
|  | HDL cholesterol levels in current drinkers | 7.00E-11 | 30698716 |
|  | High density lipoprotein cholesterol levels | 7.00E-21 | 29507422 |
|  | Obese vs. thin | 1.00E-08 | 30677029 |
|  | HDL cholesterol x physical activity interaction (2df test) | 5.00E-19 | 30670697 |
|  | HDL cholesterol levels x alcohol consumption (drinkers vs non-drinkers) interaction (2df) | 2.00E-31 | 30698716 |
|  | Multisite chronic pain | 2.00E-13 | 31194737 |
|  | Bitter alcoholic beverage consumption | 3.00E-14 | 31046077 |
|  | Predicted visceral adipose tissue | 7.00E-13 | 31501611 |
|  | Alcohol use disorder (dependence and problematic use scores) | 5.00E-15 | 30336701 |
|  | HDL cholesterol levels x alcohol consumption (regular vs non-regular drinkers) interaction (2df) | 2.00E-33 | 30698716 |
| rs1536053 | Body mass index | 8.00E-11 | 30239722 |
| rs1620977 | General cognitive ability | 3.00E-11 | 29844566 |
|  | Household income (MTAG) | 1.00E-08 | 31844048 |
|  | Educational attainment (years of education) | 8.00E-20 | 30595370 |
|  | Autism and major depressive disorder (MTAG) | 7.00E-09 | 30804558 |
|  | Bipolar disorder or body mass index | 9.00E-13 | 31754094 |
| rs17005118 | Sleep duration (short sleep) | 3.00E-09 | 30846698 |
| rs1731951 | Neurociticism | 6.00E-09 | 29500382 |
| rs176644 | Smoking status (ever vs never smokers) | 3.00E-09 | 30643258 |
| rs1861412 | Body mass index | 1.00E-32 | 30239722 |
| rs2030672 | Highest math class taken | 2.00E-08 | 30038396 |
| rs324017 | Appendicular lean mass | 5.00E-10 | 31761296 |
| rs34967082 | Educational attainment (years of education) | 2.00E-08 | 30038396 |
| rs429358 | Hippocampal volume | 2.00E-19 | 29274321 |
|  | Parental longevity (father's attained age) | 2.00E-28 | 29227965 |
|  | Total cholesterol levels | 1.00E-145 | 29507422 |
|  | Neurofibrillary tangles | 3.00E-31 | 31497858 |
|  | Alzheimer's disease progression score | 4.00E-33 | 29860282 |
|  | HDL cholesterol | 1.00E-142 | 30275531 |
|  | High density lipoprotein cholesterol levels | 3.00E-50 | 29507422 |
|  | Logical memory (immediate recall) | 2.00E-13 | 29274321 |
|  | Alzheimer disease (clinical subgroup AD+++) | 1.00E-75 | 31473137 |
|  | Cerebral amyloid deposition positivity (PET imaging) | 5.00E-20 | 26252872 |
|  | Lewy body disease | 5.00E-12 | 25188341 |
|  | Parental longevity (mother's age at death or mother's attained age) | 4.00E-57 | 31484785 |
|  | Type 2 diabetes | 3.00E-18 | 30297969 |
|  | Adult onset asthma or type 2 diabetes | 2.00E-10 | 31669095 |
|  | Alzheimer's disease biomarkers | 5.00E-14 | 23419831 |
|  | Cerebrospinal fluid AB1-42 levels | 1.00E-51 | 29274321 |
|  | Hippocampal volume | 1.00E-10 | 29860282 |
|  | Parental longevity (mother's age at death) | 7.00E-19 | 29227965 |
|  | C-reactive protein levels | 7.00E-36 | 29403010 |
|  | Advanced age-related macular degeneration | 2.00E-42 | 26691988 |
|  | Red cell distribution width | 8.00E-26 | 27863252 |
|  | Longevity (age >99th survival percentile) | 1.00E-36 | 31413261 |
|  | Cerebrospinal AB1-42 levels in Alzheimer's disease dementia | 4.00E-17 | 25027320 |
|  | Dementia with Lewy bodies | 3.00E-64 | 29263008 |
|  | Cerebrospinal AB1-42 levels in normal cognition | 9.00E-11 | 29274321 |
|  | Cerebrospinal fluid p-tau levels in mild cognitive impairment | 2.00E-11 | 29274321 |
|  | Parental longevity (father's age at death) | 9.00E-13 | 29227965 |
|  | Alzheimer's disease (onset at age over 80) | 4.00E-43 | 30979435 |
|  | Parental longevity (both parents in top 10%) | 7.00E-46 | 29227965 |
|  | Cerebrospinal fluid p-tau levels | 3.00E-18 | 29274321 |
|  | Moderate to vigorous physical activity levels | 6.00E-13 | 29899525 |
|  | Waist-hip ratio | 4.00E-37 | 30239722 |
|  | Triglycerides | 1.00E-39 | 29507422 |
|  | Waist-to-hip ratio adjusted for BMI | 3.00E-19 | 31669095 |
|  | Cerebrospinal fluid t-tau levels | 1.00E-20 | 29274321 |
|  | Refractive error | 3.00E-17 | 32231278 |
|  | Blood protein levels | 4.00E-159 | 29875488 |
|  | Parental lifespan | 3.00E-83 | 30642433 |
|  | Parental longevity (mother's attained age) | 1.00E-68 | 29227965 |
|  | Mental health study participation (completed survey) | 5.00E-19 | 31263887 |
|  | Cerebrospinal AB1-42 levels in mild cognitive impairment | 6.00E-27 | 29274321 |
|  | Neuritic plaques or cerebral amyloid angiopathy (pleiotropy) | 5.00E-40 | 29458411 |
|  | Alzheimer's disease | 2.00E-303 | 31473137 |
|  | Platelet count | 3.00E-09 | 27863252 |
|  | Parental longevity (combined parental age at death) | 5.00E-22 | 29227965 |
|  | Cortical amyloid beta load | 3.00E-50 | 29860282 |
|  | Cerebrospinal fluid t-tau levels in mild cognitive impairment | 1.00E-13 | 29274321 |
|  | HDL cholesterol levels x short total sleep time interaction (2df test) | 4.00E-37 | 31719535 |
|  | Brain imaging | 1.00E-09 | 20100581 |
|  | Neurofibrillary tangles or cerebral amyloid angiopathy (pleiotropy) | 6.00E-35 | 29458411 |
|  | Longevity (age >90th survival percentile) | 1.00E-61 | 31413261 |
|  | Logical memory (delayed recall) | 2.00E-18 | 29274321 |
|  | Low density lipoprotein cholesterol levels | 3.00E-187 | 29507422 |
|  | Neuritic plaque | 9.00E-25 | 31497858 |
|  | Cognitive decline (age-related) | 2.00E-14 | 28078323 |
|  | Mortality | 1.00E-20 | 27029810 |
|  | Cerebral amyloid deposition (PET imaging) | 8.00E-32 | 26252872 |
|  | Neuritic plaques or neurofibrillary tangles (pleiotropy) | 3.00E-47 | 29458411 |
| rs4702 | Number of sexual partners | 6.00E-17 | 30643258 |
|  | Age at first sexual intercourse | 3.00E-10 | 27089180 |
|  | Autism spectrum disorder or schizophrenia | 1.00E-10 | 28540026 |
|  | General risk tolerance (MTAG) | 3.00E-09 | 30643258 |
|  | anorexia nervosa | 2.00E-11 | 31835028 |
|  | Feeling hurt | 2.00E-09 | 29500382 |
|  | Schizophrenia | 2.00E-15 | 31740837 |
|  | Cognitive ability | 3.00E-11 | 31374203 |
| rs4858708 | Adventurousness | 4.00E-08 | 30643258 |
| rs4981170 | Waist circumference adjusted for body mass index | 3.00E-11 | 31669095 |
| rs5877 | Subjective well-being (MTAG) | 2.00E-08 | 29292387 |
| rs6119267 | Highest math class taken (MTAG) | 2.00E-10 | 30038396 |
|  | Red cell distribution width | 4.00E-30 | 27863252 |
|  | Educational attainment (MTAG) | 4.00E-10 | 30038396 |
| rs62158170 | Sleep duration | 6.00E-20 | 30531941 |
|  | Diastolic blood pressure | 7.00E-15 | 30224653 |
| rs6457796 | Body mass index | 1.00E-14 | 26426971 |
| rs66674044 | Fat-free mass | 1.00E-08 | 30593698 |
| rs7040224 | Educational attainment (years of education) | 6.00E-23 | 30038396 |
| rs77641763 | Ease of getting up in the morning | 2.00E-11 | 30804565 |
|  | Chronotype | 2.00E-12 | 27494321 |
|  | Morning vs. evening chronotype | 7.00E-09 | 27494321 |
| rs910187 | Well-being spectrum (multivariate analysis) | 2.00E-11 | 30643256 |
|  | Depressive symptoms | 4.00E-10 | 30643256 |
|  | Neuroticism | 3.00E-10 | 30643256 |
|  | Migraine | 4.00E-08 | 27182965 |
| rs9964420 | Morning person | 2.00E-37 | 30696823 |
|  | Chronotype | 2.00E-37 | 30696823 |
|  | Morningness | 4.00E-21 | 30804565 |
| **Sleep duration** |  |  |  |
| rs174560 | Serum metabolite ratios in chronic kidney disease | 2.00E-32 | 29545352 |
|  | Phosphatidylinositol levels | 1.00E-20 | 31551469 |
|  | Serum metabolite ratios in chronic kidney disease | 7.00E-18 | 29545352 |
|  | Phosphatidylcholine-ether levels | 2.00E-08 | 31551469 |
| rs1991556 | Alcohol consumption | 5.00E-23 | 31358974 |
|  | Lung function (FVC) | 1.00E-53 | 30595370 |
| rs2072727 | Morningness | 3.00E-08 | 30804565 |
|  | Morning person | 4.00E-15 | 30696823 |
|  | Chronotype | 4.00E-15 | 30696823 |

Abbreviations: Chr, chromosome; EA, effect allele; SNP, single nucleotide polymorphism.

**Supplementary Table 3. The potential secondary phenotypes of psychiatric disorders-associated SNPs from the GWAS Catalog.**

| SNP | Trait | *P* value^*^ | PMID |
| --- | --- | --- | --- |
| **Attention deficit**  **hyperactivity disorder** | |  |  |
| rs114181198 | Attention deficit hyperactivity disorder, putamen volume | 4.00E-09 | 30818988 |
| rs1484144 | Anorexia nervosa, obsessive-compulsive disorder, attention deficit hyperactivity disorder, Tourette syndrome, unipolar depression, schizophrenia, autism spectrum disorder, bipolar disorder | 4.00E-09 | 31835028 |
| rs227378 | Obsessive-compulsive disorder, attention deficit hyperactivity disorder | 2.00E-08 | 33686288 |
|  | Attention deficit hyperactivity disorder, unipolar depression | 5.00E-08 | 33686288 |
| rs145108385 | Attention deficit hyperactivity disorder, Cannabis use | 3.00E-08 | 30610198 |
|  | Upper aerodigestive tract neoplasm | 5.00E-06 | 33667223 |
|  | Squamous cell lung carcinoma | 9.00E-06 | 28604730 |
| rs4916723 | Alcohol consumption measurement | 1.00E-11 | 30643258 |
|  | Autism spectrum disorder | 3.00E-06 | 30804558 |
|  | Alcohol consumption measurement | 2.00E-10 | 31358974 |
|  | Alcohol consumption measurement | 9.00E-10 | 32451486 |
|  | Anorexia nervosa, obsessive-compulsive disorder, attention deficit hyperactivity disorder, Tourette syndrome, unipolar depression, schizophrenia, autism spectrum disorder, bipolar disorder | 2.00E-09 | 31835028 |
|  | Alcohol consumption measurement | 2.00E-11 | 30643251 |
|  | Unipolar depression, autism spectrum disorder | 5.00E-08 | 33686288 |
|  | Neuroticism measurement | 2.00E-08 | 29942085 |
| rs1592757 | Insomnia measurement | 2.00E-10 | 30804566 |
| rs141547796 | Smoking status measurement | 6.00E-10 | 30643251 |
|  | Smoking initiation | 5.00E-08 | 30643251 |
|  | Age at first sexual intercourse measurement | 1.00E-17 | 34211149 |
| rs74760947 | Attention deficit hyperactivity disorder, Cannabis use | 2.00E-10 | 30610198 |
|  | Attention deficit hyperactivity disorder, unipolar depression | 2.00E-08 | 33686288 |
| rs713240 | Attention deficit hyperactivity disorder, schizophrenia | 5.00E-09 | 33686288 |
| rs12265655 | Mental or behavioural disorder | 1.00E-08 | 32534018 |
| rs4275621 | Smoking status measurement | 4.00E-16 | 30643251 |
| rs11245604 | Serum alanine aminotransferase measurement | 4.00E-09 | 34594039 |
| rs10400419 | Total cortical area measurement | 2.00E-11 | 32198502 |
|  | Axial length measurement | 2.00E-07 | 24144296 |
| rs1427829 | Self reported educational attainment | 2.00E-13 | 30038396 |
| rs8039398 | Attention deficit hyperactivity disorder, Cannabis use | 4.00E-11 | 30610198 |
|  | Attention deficit hyperactivity disorder, unipolar depression | 1.00E-08 | 33686288 |
| rs8058677 | Attention deficit hyperactivity disorder, unipolar depression | 4.00E-08 | 33686288 |
| rs212178 | Attention deficit hyperactivity disorder, unipolar depression | 3.00E-08 | 33686288 |
| **Bipolar disorder** |  |  |  |
| rs9834970 | Schizophrenia | 4.00E-14 | 32606422 |
|  | Anorexia nervosa, obsessive-compulsive disorder, attention deficit hyperactivity disorder, Tourette syndrome, unipolar depression, schizophrenia, autism spectrum disorder | 2.00E-17 | 31835028 |
|  | Lifestyle measurement | 3.00E-09 | 32317632 |
| rs2302417 | Pulse pressure measurement | 2.00E-08 | 30578418 |
|  | Body mass index | 3.00E-16 | 31669095 |
| rs2388334 | Risk-taking behavior | 2.00E-10 | 30643258 |
|  | Autism spectrum disorder, self reported educational attainment | 3.00E-12 | 30804558 |
|  | Intelligence | 2.00E-30 | 29844566 |
|  | Anorexia nervosa, obsessive-compulsive disorder, attention deficit hyperactivity disorder, Tourette syndrome, unipolar depression, schizophrenia, autism spectrum disorder | 1.00E-11 | 31835028 |
| rs113779084 | Self reported educational attainment | 3.00E-08 | 27225129 |
| **Major depressive disorder** | |  |  |
| rs12129573 | Anorexia nervosa, obsessive-compulsive disorder, attention deficit hyperactivity disorder, Tourette syndrome, unipolar depression, schizophrenia, autism spectrum disorder, bipolar disorder | 5.00E-17 | 31835028 |
|  | Schizophrenia | 9.00E-15 | 29483656 |
| rs159963 | Neutrophil percentage of granulocytes | 4.00E-16 | 27863252 |
|  | Eosinophil count | 6.00E-28 | 30595370 |
|  | Respiratory system disease | 1.00E-11 | 30595370 |
| rs1226412 | Unipolar depression, bipolar disorder | 1.00E-08 | 31926635 |
|  | Anorexia nervosa, obsessive-compulsive disorder, attention deficit hyperactivity disorder, Tourette syndrome, unipolar depression, schizophrenia, autism spectrum disorder, bipolar disorder | 2.00E-08 | 31835028 |
| rs11682175 | Irritability measurement | 1.00E-08 | 29500382 |
|  | Schizophrenia | 1.00E-12 | 26198764 |
|  | Schizophrenia, autism spectrum disorder | 1.00E-12 | 28540026 |
|  | Bipolar disorder | 1.00E-09 | 32606422 |
|  | Sleep duration | 1.00E-13 | 30804565 |
| rs7430565 | Unipolar depression, bipolar disorder | 2.00E-11 | 31926635 |
| rs34215985 | Anorexia nervosa, obsessive-compulsive disorder, attention deficit hyperactivity disorder, Tourette syndrome, unipolar depression, schizophrenia, autism spectrum disorder, bipolar disorder | 3.00E-10 | 31835028 |
| rs116755193 | Anorexia nervosa, obsessive-compulsive disorder, attention deficit hyperactivity disorder, Tourette syndrome, unipolar depression, schizophrenia, autism spectrum disorder, bipolar disorder | 1.00E-08 | 31835028 |
| rs11135349 | Unipolar depression, bipolar disorder | 3.00E-11 | 31926635 |
| rs4869056 | Unipolar depression, bipolar disorder | 5.00E-09 | 31926635 |
| rs10950398 | Blood protein measurement | 6.00E-27 | 30072576 |
| rs61867293 | Anorexia nervosa, obsessive-compulsive disorder, attention deficit hyperactivity disorder, Tourette syndrome, unipolar depression, schizophrenia, autism spectrum disorder, bipolar disorder | 8.00E-15 | 31835028 |
| rs1806153 | Unipolar depression, bipolar disorder | 3.00E-09 | 31926635 |
|  | Neuroticism measurement | 2.00E-13 | 29255261 |
|  | Anorexia nervosa, obsessive-compulsive disorder, attention deficit hyperactivity disorder, Tourette syndrome, unipolar depression, schizophrenia, autism spectrum disorder, bipolar disorder | 1.00E-09 | 31835028 |
|  | Wellbeing measurement | 5.00E-14 | 30643256 |
| rs4074723 | Unipolar depression, bipolar disorder | 3.00E-09 | 31926635 |
| rs4143229 | Unipolar depression, bipolar disorder | 3.00E-10 | 31926635 |
| rs12552 | Unipolar depression, bipolar disorder | 1.00E-23 | 31926635 |
|  | Intelligence | 3.00E-08 | 29326435 |
|  | Anorexia nervosa, obsessive-compulsive disorder, attention deficit hyperactivity disorder, Tourette syndrome, unipolar depression, schizophrenia, autism spectrum disorder, bipolar disorder | 1.00E-08 | 31835028 |
|  | Self reported educational attainment | 5.00E-09 | 30038396 |
|  | Unipolar depression | 6.00E-19 | 29700475 |
| rs915057 | Anorexia nervosa, obsessive-compulsive disorder, attention deficit hyperactivity disorder, Tourette syndrome, unipolar depression, schizophrenia, autism spectrum disorder, bipolar disorder | 3.00E-09 | 31835028 |
| rs10149470 | Unipolar depression, bipolar disorder | 1.00E-10 | 31926635 |
|  | Intelligence | 5.00E-11 | 29326435 |
|  | Anorexia nervosa, obsessive-compulsive disorder, attention deficit hyperactivity disorder, Tourette syndrome, unipolar depression, schizophrenia, autism spectrum disorder, bipolar disorder | 5.00E-13 | 31835028 |
|  | Intelligence, self reported educational attainment | 2.00E-09 | 29186694 |
| rs8063603 | Unipolar depression, bipolar disorder | 5.00E-11 | 31926635 |
| rs7200826 | Anorexia nervosa, obsessive-compulsive disorder, attention deficit hyperactivity disorder, Tourette syndrome, unipolar depression, schizophrenia, autism spectrum disorder, bipolar disorder | 1.00E-08 | 31835028 |
|  | Neuroticism measurement | 4.00E-09 | 30643256 |
|  | Wellbeing measurement | 3.00E-09 | 30643256 |
|  | Unipolar depression | 2.00E-08 | 29700475 |
| rs11643192 | Unipolar depression, bipolar disorder | 1.00E-11 | 31926635 |
|  | Chronotype measurement | 1.00E-09 | 30804565 |
| rs62099069 | Unipolar depression, bipolar disorder | 1.00E-09 | 31926635 |
| rs11663393 | Unipolar depression, bipolar disorder | 2.00E-10 | 31926635 |
| rs12958048 | Unipolar depression, bipolar disorder | 5.00E-14 | 31926635 |
|  | Anorexia nervosa, obsessive-compulsive disorder, attention deficit hyperactivity disorder, Tourette syndrome, unipolar depression, schizophrenia, autism spectrum disorder, bipolar disorder | 1.00E-12 | 31835028 |
|  | Heel bone mineral density | 1.00E-09 | 30598549 |
|  | Neuroticism measurement | 3.00E-12 | 29942085 |
| rs5758265 | Schizophrenia | 8.00E-10 | 32606422 |
|  | Anorexia nervosa, obsessive-compulsive disorder, attention deficit hyperactivity disorder, Tourette syndrome, unipolar depression, schizophrenia, autism spectrum disorder, bipolar disorder | 5.00E-14 | 31835028 |
| **Obsessive Compulsive Disorder** | |  |  |
| rs12568997 | Obsessive-compulsive disorder, autism spectrum disorder | 3.00E-08 | 33686288 |
| rs12504244 | Anorexia nervosa, obsessive-compulsive disorder | 4.00E-07 | 30087453 |
| **Post-traumatic stress disorder** | |  |  |
| rs36127550 | Waist-hip ratio | 1.00E-10 | 30595370 |
| rs1136201 | Glomerular filtration rate | 8.00E-29 | 31451708 |
| **Schizophrenia** |  |  |  |
| rs12129573 | Anorexia nervosa, obsessive-compulsive disorder, attention deficit hyperactivity disorder, Tourette syndrome, unipolar depression, autism spectrum disorder, bipolar disorder | 5.00E-17 | 31835028 |
|  | Unipolar depression | 4.00E-12 | 29700475 |
| rs140505938 | Anorexia nervosa, obsessive-compulsive disorder, attention deficit hyperactivity disorder, Tourette syndrome, unipolar depression, autism spectrum disorder, bipolar disorder | 2.00E-12 | 31835028 |
| rs6694545 | Anorexia nervosa, obsessive-compulsive disorder, attention deficit hyperactivity disorder, Tourette syndrome, unipolar depression, autism spectrum disorder, bipolar disorder | 4.00E-09 | 31835028 |
| rs12991836 | Intelligence, self reported educational attainment | 7.00E-10 | 31374203 |
| rs1451488 | Intelligence, self reported educational attainment | 4.00E-08 | 31374203 |
| rs2949006 | Intelligence, self reported educational attainment | 3.00E-13 | 31374203 |
|  | Red blood cell distribution width | 1.00E-10 | 32888493 |
| rs6434928 | Bipolar disorder | 8.00E-10 | 32606422 |
| rs7596038 | Neuroticism measurement | 3.00E-09 | 29255261 |
| rs1080500 | Autism spectrum disorder | 6.00E-09 | 28540026 |
| rs35346733 | Bipolar disorder | 5.00E-10 | 32606422 |
|  | Anorexia nervosa, obsessive-compulsive disorder, attention deficit hyperactivity disorder, Tourette syndrome, unipolar depression, autism spectrum disorder, bipolar disorder | 8.00E-10 | 31835028 |
|  | Chronotype measurement | 4.00E-10 | 30696823 |
| rs7432375 | Feeling nervous measurement | 7.00E-09 | 29500382 |
|  | Neuroticism measurement | 4.00E-14 | 29255261 |
|  | Intelligence, self reported educational attainment | 6.00E-10 | 31374203 |
|  | Autism spectrum disorder | 3.00E-08 | 28540026 |
| rs75968099 | Autism spectrum disorder | 1.00E-11 | 28540026 |
| rs9881798 | Neuroticism measurement | 9.00E-10 | 29942085 |
| rs10520163 | Autism spectrum disorder | 9.00E-09 | 28540026 |
| rs13107325 | Risk-taking behaviour | 4.00E-13 | 30643258 |
|  | Abnormality of refraction | 4.00E-17 | 32231278 |
|  | BMI-adjusted waist-hip ratio | 3.00E-19 | 31669095 |
|  | Hemoglobin measurement | 1.00E-38 | 32888493 |
|  | Systolic blood pressure, alcohol drinking | 1.00E-15 | 29912962 |
|  | Physical activity measurement, body mass index | 2.00E-10 | 28448500 |
|  | Osteoarthritis | 8.00E-19 | 30664745 |
|  | Diastolic blood pressure | 5.00E-33 | 27841878 |
|  | Smoking behavior, body mass index | 2.00E-10 | 28443625 |
|  | Mean arterial pressure, alcohol drinking | 4.00E-11 | 29912962 |
|  | Body mass index | 4.00E-47 | 30239722 |
|  | Metabolic syndrome | 4.00E-10 | 31589552 |
|  | Body fat percentage | 4.00E-10 | 30593698 |
|  | Sleep duration | 3.00E-13 | 30846698 |
|  | Body height | 1.00E-21 | 30595370 |
|  | Cortical thickness, neuroimaging measurement | 8.00E-38 | 32665545 |
|  | Cortical surface area measurement, neuroimaging measurement | 5.00E-20 | 32665545 |
|  | Red blood cell density measurement | 1.00E-16 | 32888493 |
|  | Agents acting on the renin-angiotensin system use measurement | 4.00E-10 | 31015401 |
|  | Self reported educational attainment | 2.00E-24 | 30038396 |
|  | Cognitive function measurement | 1.00E-23 | 30038396 |
|  | Waist-hip ratio | 1.00E-20 | 30595370 |
|  | Eczema | 2.00E-10 | 30595370 |
|  | Systolic blood pressure | 1.00E-36 | 30595370 |
|  | Mathematical ability | 5.00E-10 | 30038396 |
|  | Erythrocyte count | 6.00E-24 | 30595370 |
|  | Vital capacity | 9.00E-18 | 30595370 |
|  | Low density lipoprotein cholesterol measurement | 1.00E-10 | 32154731 |
|  | Hematocrit | 2.00E-40 | 32888493 |
|  | Triglyceride measurement | 6.00E-15 | 32203549 |
|  | Nucleus accumbens volume | 8.00E-10 | 31636452 |
|  | Liver fat measurement, liver disease biomarker | 1.00E-133 | 32247823 |
|  | Alcohol use disorder measurement | 8.00E-19 | 32451486 |
|  | Mean arterial pressure | 1.00E-11 | 27618448 |
|  | Total cholesterol measurement | 5.00E-11 | 29507422 |
|  | Cardiovascular disease biomarker measurement | 6.00E-10 | 26908625 |
|  | Apolipoprotein A 1 measurement | 5.00E-82 | 32203549 |
|  | High density lipoprotein cholesterol measurement | 2.00E-108 | 32203549 |
|  | Lifestyle measurement | 2.00E-12 | 32317632 |
|  | Brain volume measurement | 2.00E-18 | 31676860 |
|  | Refractive error measurement | 3.00E-10 | 32352494 |
|  | Hypertension | 5.00E-12 | 31879980 |
|  | Intelligence | 2.00E-21 | 29942086 |
|  | Type II diabetes mellitus | 3.00E-08 | 32541925 |
|  | Diastolic blood pressure, alcohol drinking | 2.00E-23 | 29912962 |
|  | Alcohol consumption measurement | 1.00E-24 | 30643258 |
|  | Balding measurement | 4.00E-20 | 30573740 |
|  | Grip strength measurement | 4.00E-23 | 29691431 |
|  | Neuroimaging measurement, brain volume measurement | 5.00E-73 | 32665545 |
|  | Brain measurement, neuroimaging measurement | 3.00E-124 | 32665545 |
|  | Neuroimaging measurement | 9.00E-18 | 30649180 |
|  | Alcohol use disorder measurement, longitudinal alcohol consumption measurement | 2.00E-30 | 30940813 |
|  | BMI-adjusted waist circumference | 5.00E-20 | 31669095 |
| rs111294930 | Anorexia nervosa, obsessive-compulsive disorder, attention deficit hyperactivity disorder, Tourette syndrome, unipolar depression, autism spectrum disorder, bipolar disorder | 3.00E-10 | 31835028 |
| rs7701440 | Self reported educational attainment | 4.00E-08 | 27046643 |
| rs1339227 | Autism spectrum disorder | 3.00E-08 | 28540026 |
| rs12704290 | Bipolar disorder | 1.00E-09 | 32606422 |
|  | Intelligence, self reported educational attainment | 8.00E-09 | 31374203 |
|  | Autism spectrum disorder | 1.00E-10 | 28540026 |
|  | Anorexia nervosa, obsessive-compulsive disorder, attention deficit hyperactivity disorder, Tourette syndrome, unipolar depression, autism spectrum disorder, bipolar disorder | 3.00E-10 | 31835028 |
| rs3735025 | Autism spectrum disorder | 6.00E-10 | 28540026 |
| rs7801375 | Autism spectrum disorder | 3.00E-10 | 28540026 |
| rs11783093 | Smoking behavior measurement | 1.00E-16 | 31689377 |
|  | Smoking behavior | 7.00E-22 | 30643258 |
|  | Smoking initiation | 1.00E-31 | 30643251 |
| rs12416331 | Pulse pressure measurement | 6.00E-42 | 30578418 |
|  | Mean arterial pressure | 7.00E-15 | 30487518 |
| rs7893279 | Autism spectrum disorder | 3.00E-10 | 28540026 |
| rs2514218 | Unipolar depression, bipolar disorder | 3.00E-10 | 31926635 |
|  | Bipolar disorder | 5.00E-09 | 32606422 |
|  | Intelligence, self reported educational attainment | 2.00E-09 | 31374203 |
|  | Anorexia nervosa, obsessive-compulsive disorder, attention deficit hyperactivity disorder, Tourette syndrome, unipolar depression, autism spectrum disorder, bipolar disorder | 2.00E-11 | 31835028 |
| rs35774874 | Anorexia nervosa, obsessive-compulsive disorder, attention deficit hyperactivity disorder, Tourette syndrome, unipolar depression, autism spectrum disorder, bipolar disorder | 2.00E-09 | 31835028 |
| rs2007044 | Autism spectrum disorder | 6.00E-14 | 28540026 |
|  | Intelligence, self reported educational attainment | 2.00E-15 | 31374203 |
| rs2851447 | Autism spectrum disorder | 8.00E-12 | 28540026 |
| rs2332700 | Autism spectrum disorder | 2.00E-09 | 28540026 |
|  | Anorexia nervosa, obsessive-compulsive disorder, attention deficit hyperactivity disorder, Tourette syndrome, unipolar depression, autism spectrum disorder, bipolar disorder | 5.00E-10 | 31835028 |
| rs17514846 | Coronary artery disease | 8.00E-27 | 29212778 |
|  | Systolic blood pressure | 5.00E-09 | 30487518 |
|  | Hypertension | 3.00E-09 | 30487518 |
|  | Mean arterial pressure | 2.00E-10 | 27618448 |
|  | Parental longevity | 7.00E-10 | 29227965 |
| rs42945 | Testosterone measurement | 4.00E-08 | 32042192 |
| rs7191183 | Autism spectrum disorder | 2.00E-09 | 28540026 |
| rs4925114 | Body mass index | 3.00E-08 | 28892062 |
|  | Intelligence | 2.00E-08 | 29844566 |
| rs56775891 | Self reported educational attainment | 7.00E-18 | 30038396 |
| rs28758902 | Irritability measurement | 4.00E-08 | 29500382 |
|  | BMI-adjusted waist-hip ratio | 1.00E-09 | 31669095 |
|  | Autism spectrum disorder | 3.00E-08 | 28540026 |
| rs72986630 | Intelligence, self reported educational attainment | 5.00E-09 | 31374203 |
|  | Autism spectrum disorder | 5.00E-08 | 28540026 |
| rs6065094 | Intelligence, self reported educational attainment | 9.00E-11 | 31374203 |
| rs5757730 | Self reported educational attainment | 2.00E-10 | 30038396 |

* The SNPs for bipolar disorder, major depressive disorder, schizophrenia were associated with secondary traits at *P*<5×10^-8^ by searching GWAS Catalog; the SNPs for attention-deficit/hyperactivity disorder, autism spectrum disorder, obsessive compulsive disorder and post-traumatic stress disorder were at *P*<5×10^-6^ by searching GWAS Catalog.

Abbreviations: Chr, chromosome; EA, effect allele; SNP, single nucleotide polymorphism.

| **Supplementary Table 4. Characteristics of the associations of instrumental variables for three sleep-associated traits** | | | | | | | | | |
| --- | --- | --- | --- | --- | --- | --- | --- | --- | --- |
| Traits | SNP | Chr | Position | Closest gene | Effect allele | Other allele | β | SE | *P*-value |
| Chronotype | rs909757 | 1 | 4850823 | AJAP1 | T | C | 0.020 | 0.003 | 1.03E-08 |
| Chronotype | rs17448682 | 1 | 15966713 | DDI2 | T | C | 0.035 | 0.004 | 2.82E-16 |
| Chronotype | rs10917513 | 1 | 20006887 | HTR6,TMCO4 | T | C | -0.031 | 0.004 | 3.15E-15 |
| Chronotype | rs11208844 | 1 | 66851147 | PDE4B,SGIP1 | A | G | -0.029 | 0.005 | 5.38E-09 |
| Chronotype | rs12040629 | 1 | 77705365 | PIGK,AK5 | A | G | 0.073 | 0.005 | 4.71E-45 |
| Chronotype | rs11588913 | 1 | 79963816 | ELTD1, | A | G | -0.024 | 0.004 | 3.03E-10 |
| Chronotype | rs481214 | 1 | 93469865 | FAM69A,MTF2 | A | T | 0.023 | 0.004 | 2.05E-08 |
| Chronotype | rs11165655 | 1 | 96959104 | PTBP2 | A | G | -0.028 | 0.004 | 4.78E-13 |
| Chronotype | rs17575798 | 1 | 110086451 | GPR61 | A | G | -0.034 | 0.004 | 3.66E-14 |
| Chronotype | rs6690292 | 1 | 113188419 | CAPZA1 | T | C | -0.025 | 0.004 | 1.54E-08 |
| Chronotype | rs11102807 | 1 | 115061584 | TRIM33,BCAS2 | A | G | -0.022 | 0.004 | 8.09E-09 |
| Chronotype | rs9436119 | 1 | 150467753 | TARS2 | A | G | 0.040 | 0.003 | 5.70E-30 |
| Chronotype | rs115073088 | 1 | 174215858 | RABGAP1L | A | G | -0.076 | 0.011 | 4.31E-12 |
| Chronotype | rs6429233 | 1 | 241137033 | RGS7 | A | G | 0.020 | 0.004 | 1.20E-08 |
| Chronotype | rs1144566 | 1 | 182569626 | RGS16 | T | C | 0.231 | 0.011 | 2.00E-95 |
| Chronotype | rs13011556 | 2 | 4651923 | ALLC, | C | G | -0.029 | 0.004 | 1.14E-13 |
| Chronotype | rs17396357 | 2 | 48252311 | FBXO11,FOXN2 | T | C | 0.021 | 0.004 | 2.28E-08 |
| Chronotype | rs4672458 | 2 | 53736362 | ASB3 | T | C | -0.022 | 0.004 | 1.66E-10 |
| Chronotype | rs13414393 | 2 | 54275162 | PSME4,ACYP2 | T | C | -0.022 | 0.004 | 6.16E-09 |
| Chronotype | rs10175975 | 2 | 59429807 | FANCL | T | C | 0.025 | 0.004 | 1.11E-09 |
| Chronotype | rs359248 | 2 | 60477461 | BCL11A | T | G | -0.028 | 0.003 | 2.23E-16 |
| Chronotype | rs12464387 | 2 | 75445544 | TACR1,EVA1A | A | G | -0.021 | 0.004 | 1.06E-09 |
| Chronotype | rs6727752 | 2 | 76361783 | GCFC2,,LRRTM4 | A | G | 0.026 | 0.004 | 2.27E-10 |
| Chronotype | rs10520176 | 2 | 77217310 | LRRTM4 | T | C | 0.038 | 0.004 | 4.54E-24 |
| Chronotype | rs76064513 | 2 | 125438641 | CNTP5 | T | C | 0.034 | 0.006 | 3.09E-08 |
| Chronotype | rs77248969 | 2 | 136490731 | R3HDM1,UBXN4 | A | G | -0.033 | 0.006 | 4.91E-09 |
| Chronotype | rs28380327 | 2 | 144232491 | ARHGAP15 | A | T | 0.040 | 0.004 | 5.24E-25 |
| Chronotype | rs2166559 | 2 | 149551658 | EPC2,KIF5C | T | C | -0.033 | 0.005 | 1.56E-09 |
| Chronotype | rs747003 | 2 | 161916409 | RBMS1,TANK | T | C | 0.020 | 0.004 | 2.78E-08 |
| Chronotype | rs4666682 | 2 | 186203743 | ZNF804A,FSIP2 | A | G | -0.025 | 0.004 | 3.68E-09 |
| Chronotype | rs11677484 | 2 | 191578172 | B1,GLS | T | G | 0.023 | 0.004 | 1.18E-09 |
| Chronotype | rs184033703 | 2 | 206956138 | INO80D,NDUFS1 | A | G | 0.058 | 0.008 | 1.31E-13 |
| Chronotype | rs80271258 | 2 | 239311505 | TRAF3IP1,ASB1 | T | C | -0.089 | 0.006 | 4.16E-43 |
| Chronotype | rs62182135 | 2 | 240267305 | HDAC4 | A | C | -0.024 | 0.003 | 1.13E-12 |
| Chronotype | rs6794796 | 3 | 14383632 | LSM3,SLC6A6 | A | G | 0.025 | 0.004 | 3.07E-10 |
| Chronotype | rs9817910 | 3 | 18246870 | LOC339862 | A | G | -0.022 | 0.003 | 1.78E-10 |
| Chronotype | rs73050286 | 3 | 23224684 | UBE2E2 | T | C | 0.030 | 0.004 | 3.52E-11 |
| Chronotype | rs2362775 | 3 | 24924421 | THRB,RARB | T | C | -0.022 | 0.004 | 6.03E-09 |
| Chronotype | rs114848860 | 3 | 36859494 | DCLK3,TRANK1 | A | T | -0.077 | 0.010 | 1.19E-13 |
| Chronotype | rs12636669 | 3 | 50003323 | RBM6 | T | C | 0.057 | 0.006 | 5.26E-19 |
| Chronotype | rs7429614 | 3 | 77205438 | ROBO2 | T | G | 0.035 | 0.004 | 2.45E-19 |
| Chronotype | rs112201801 | 3 | 82591379 | GBE1 | T | C | -0.085 | 0.013 | 9.87E-11 |
| Chronotype | rs1449403 | 3 | 85591467 | CADM2 | A | G | 0.042 | 0.006 | 6.42E-13 |
| Chronotype | rs1398346 | 3 | 110271943 | LOC151760 | T | C | 0.026 | 0.005 | 4.35E-08 |
| Chronotype | rs72950188 | 3 | 116103275 | LSAMP | T | C | 0.045 | 0.007 | 1.67E-10 |
| Chronotype | rs13065394 | 3 | 132971327 | TMEM108 | T | G | -0.027 | 0.004 | 4.14E-11 |
| Chronotype | rs1599374 | 3 | 160891727 | B3GALNT1,NMD3 | A | G | 0.031 | 0.004 | 3.09E-15 |
| Chronotype | rs3850174 | 3 | 172364093 | NCEH1 | A | T | -0.035 | 0.004 | 2.88E-15 |
| Chronotype | rs9836621 | 3 | 182096311 | SOX2,ATP11B | T | C | -0.028 | 0.004 | 1.84E-13 |
| Chronotype | rs1468945 | 3 | 185990392 | DGKG | A | G | -0.036 | 0.004 | 5.81E-16 |
| Chronotype | rs4690085 | 4 | 2697300 | FAM193A | A | G | -0.019 | 0.003 | 2.02E-08 |
| Chronotype | rs4698678 | 4 | 18260776 | LCORL | C | G | 0.031 | 0.005 | 1.25E-11 |
| Chronotype | rs1502249 | 4 | 27495379 | STIM2 | A | G | 0.017 | 0.003 | 2.72E-08 |
| Chronotype | rs6838677 | 4 | 66520667 | EPHA5 | A | C | -0.021 | 0.004 | 2.68E-08 |
| Chronotype | rs4860734 | 4 | 67096904 | EPHA5 | A | G | 0.020 | 0.004 | 2.66E-08 |
| Chronotype | rs6816922 | 4 | 80206272 | PAQR3,A11 | A | C | -0.020 | 0.004 | 4.26E-08 |
| Chronotype | rs6846730 | 4 | 83279041 | HNRNPD | T | C | -0.032 | 0.004 | 2.54E-14 |
| Chronotype | rs2850979 | 4 | 102094764 | PPP3CA | T | C | -0.023 | 0.004 | 3.21E-09 |
| Chronotype | rs7700110 | 4 | 114439894 | CAMK2D | A | G | 0.024 | 0.004 | 4.11E-09 |
| Chronotype | rs17455138 | 4 | 130903511 | C4orf33 | T | C | 0.031 | 0.005 | 1.04E-10 |
| Chronotype | rs9991917 | 4 | 132512118 | / | A | T | 0.045 | 0.008 | 1.12E-08 |
| Chronotype | rs4241964 | 4 | 137053959 | / | T | G | -0.029 | 0.004 | 8.74E-16 |
| Chronotype | rs938836 | 4 | 139939653 | CCRN4L | A | G | -0.021 | 0.003 | 1.01E-09 |
| Chronotype | rs72729847 | 4 | 147296930 | SLC10A7 | T | C | -0.030 | 0.005 | 1.35E-10 |
| Chronotype | rs9997394 | 4 | 163704083 | FSTL5,F1 | A | G | -0.025 | 0.004 | 1.05E-09 |
| Chronotype | rs7701529 | 5 | 63861475 | RGS7BP | A | T | -0.030 | 0.004 | 5.10E-12 |
| Chronotype | rs7721608 | 5 | 76581258 | PDE8B | T | G | 0.020 | 0.003 | 2.01E-09 |
| Chronotype | rs4269995 | 5 | 87701223 | TMEM161B,MEF2C | T | C | -0.034 | 0.004 | 6.44E-20 |
| Chronotype | rs1559253 | 5 | 106657015 | EF5 | A | G | 0.022 | 0.004 | 5.48E-09 |
| Chronotype | rs13172141 | 5 | 122990902 | CSNK1G3,ZNF608 | A | T | 0.022 | 0.004 | 1.82E-09 |
| Chronotype | rs67988891 | 5 | 152204741 | NMUR2,GRIA1 | C | G | -0.036 | 0.004 | 1.63E-18 |
| Chronotype | rs2901796 | 5 | 163330708 | MAT2B | A | G | 0.025 | 0.004 | 6.06E-10 |
| Chronotype | rs12518401 | 5 | 173539588 | HMP19,MSX2 | A | G | -0.024 | 0.004 | 1.58E-10 |
| Chronotype | rs465670 | 5 | 176877624 | PRR7 | T | C | 0.024 | 0.004 | 2.74E-10 |
| Chronotype | rs9394154 | 6 | 11574374 | TMEM170B | C | G | -0.022 | 0.004 | 8.35E-10 |
| Chronotype | rs9381812 | 6 | 13183998 | PHACTR1 | A | G | -0.050 | 0.004 | 1.15E-33 |
| Chronotype | rs1811899 | 6 | 14878060 | CD83,JARID2 | T | C | -0.030 | 0.005 | 1.32E-09 |
| Chronotype | rs9465253 | 6 | 19102247 | RNF144B,ID4 | T | C | 0.023 | 0.004 | 3.00E-09 |
| Chronotype | rs486416 | 6 | 31856070 | EHMT2 | A | G | -0.020 | 0.003 | 1.39E-09 |
| Chronotype | rs12206814 | 6 | 41517457 | FOXP4 | C | G | 0.025 | 0.004 | 1.17E-08 |
| Chronotype | rs2396004 | 6 | 43355851 | ZNF318,ABCC10 | A | G | 0.021 | 0.004 | 3.04E-09 |
| Chronotype | rs1931814 | 6 | 62589167 | KHDRBS2 | A | G | 0.026 | 0.004 | 2.99E-13 |
| Chronotype | rs2881955 | 6 | 72479263 | OGFRL1,RIMS1 | T | C | 0.027 | 0.004 | 5.23E-11 |
| Chronotype | rs12195792 | 6 | 98705295 | MMS22L,POU3F2 | A | T | 0.034 | 0.004 | 4.80E-15 |
| Chronotype | rs11154718 | 6 | 99592404 | FBXL4,FAXC | T | C | -0.023 | 0.004 | 1.40E-09 |
| Chronotype | rs4535583 | 6 | 115699280 | FRK | T | C | 0.021 | 0.004 | 3.68E-08 |
| Chronotype | rs2050185 | 6 | 147936781 | SAMD5,SASH1 | A | G | 0.022 | 0.004 | 6.62E-09 |
| Chronotype | rs9479402 | 6 | 153135339 | VIP,FBXO5 | T | C | -0.219 | 0.018 | 4.05E-33 |
| Chronotype | rs9347926 | 6 | 165195547 | C6orf118 | A | T | 0.026 | 0.004 | 1.28E-13 |
| Chronotype | rs4027217 | 7 | 14093914 | ETV1,DGKB | A | C | -0.026 | 0.005 | 4.46E-08 |
| Chronotype | rs10237162 | 7 | 24085405 | STK31,,NPY | T | C | 0.037 | 0.004 | 1.86E-18 |
| Chronotype | rs10951325 | 7 | 32265545 | PDE1C | T | C | 0.034 | 0.004 | 7.80E-20 |
| Chronotype | rs6967481 | 7 | 50642701 | DDC,GRB10 | T | C | 0.032 | 0.003 | 8.39E-21 |
| Chronotype | rs4236237 | 7 | 69936477 | AUTS2 | A | C | -0.024 | 0.004 | 1.04E-10 |
| Chronotype | rs2944831 | 7 | 71779635 | CALN1 | A | G | 0.025 | 0.004 | 8.50E-11 |
| Chronotype | rs3807651 | 7 | 77823771 | MAGI2 | A | T | 0.025 | 0.004 | 8.77E-09 |
| Chronotype | rs10254050 | 7 | 96468077 | SHFM1,DLX6 | C | G | -0.058 | 0.005 | 3.58E-29 |
| Chronotype | rs17302081 | 7 | 115673079 | TFEC,TES | T | C | 0.022 | 0.004 | 8.21E-09 |
| Chronotype | rs6968240 | 7 | 121942674 | FEZF1 | A | C | 0.022 | 0.003 | 2.64E-10 |
| Chronotype | rs62465218 | 7 | 132294312 | PLX4,CHCHD3 | A | C | -0.027 | 0.005 | 9.74E-09 |
| Chronotype | rs6958557 | 7 | 133585794 | EXOC4 | T | G | 0.026 | 0.004 | 4.04E-12 |
| Chronotype | rs62479736 | 8 | 3654320 | CSMD1 | T | G | 0.024 | 0.004 | 4.10E-10 |
| Chronotype | rs35524253 | 8 | 4823608 | CSMD1 | A | G | 0.034 | 0.004 | 3.19E-18 |
| Chronotype | rs2979139 | 8 | 8268313 | SGK223,CLDN23 | A | G | -0.027 | 0.004 | 3.92E-14 |
| Chronotype | rs2322605 | 8 | 27164449 | TRIM35 | A | G | -0.022 | 0.004 | 2.43E-09 |
| Chronotype | rs71523448 | 8 | 31817493 | WRN,NRG1 | C | G | -0.050 | 0.007 | 1.62E-13 |
| Chronotype | rs6993892 | 8 | 33729200 | DUSP26, | T | C | -0.035 | 0.004 | 5.24E-20 |
| Chronotype | rs7845620 | 8 | 53129069 | ST18 | A | C | -0.043 | 0.005 | 8.09E-19 |
| Chronotype | rs10109566 | 8 | 59800446 | TOX | A | G | -0.022 | 0.004 | 8.65E-09 |
| Chronotype | rs34054660 | 8 | 65015659 | YTHDF3,BHLHE22 | A | G | 0.025 | 0.005 | 4.48E-08 |
| Chronotype | rs187028 | 8 | 73459513 | KCNB2 | A | T | -0.022 | 0.003 | 2.15E-10 |
| Chronotype | rs16939162 | 8 | 76653156 | HNF4G,ZFHX4 | A | G | 0.038 | 0.005 | 1.23E-14 |
| Chronotype | rs6988733 | 8 | 91535686 | CALB1,TMEM64 | T | C | 0.023 | 0.004 | 2.79E-08 |
| Chronotype | rs1871729 | 8 | 136223702 | ZFAT,KHDRBS3 | A | G | -0.023 | 0.004 | 7.76E-09 |
| Chronotype | rs6477309 | 9 | 8450638 | PTPRD | T | C | 0.031 | 0.004 | 4.64E-15 |
| Chronotype | rs2844016 | 9 | 24582747 | IZUMO3 | T | C | 0.027 | 0.004 | 2.41E-09 |
| Chronotype | rs308521 | 9 | 37367094 | ZCCHC7,GRHPR | T | C | 0.028 | 0.003 | 3.67E-17 |
| Chronotype | rs4878734 | 9 | 38010085 | SHB | A | T | 0.022 | 0.004 | 4.69E-08 |
| Chronotype | rs6560218 | 9 | 74245426 | TRPM3,TMEM2 | T | C | -0.022 | 0.004 | 1.39E-08 |
| Chronotype | rs12378543 | 9 | 83196097 | TLE4, | T | C | -0.023 | 0.004 | 4.77E-09 |
| Chronotype | rs555784 | 9 | 85318704 | SPATA31D1,,RASEF | A | T | -0.025 | 0.004 | 3.18E-10 |
| Chronotype | rs3138490 | 9 | 92219000 | SEMA4D,GADD45G | A | T | 0.024 | 0.004 | 6.98E-10 |
| Chronotype | rs10759208 | 9 | 109806199 | ZNF462,RAD23B | T | C | -0.025 | 0.004 | 4.42E-10 |
| Chronotype | rs10818834 | 9 | 126317324 | DENND1A | T | C | 0.030 | 0.004 | 4.73E-12 |
| Chronotype | rs10988239 | 9 | 131943440 | IER5L,C9orf106 | T | C | -0.021 | 0.003 | 3.26E-11 |
| Chronotype | rs28458909 | 9 | 140257189 | EXD3 | T | C | -0.070 | 0.006 | 9.52E-34 |
| Chronotype | rs497338 | 10 | 804315 | DIP2C,LARP4B | T | C | 0.027 | 0.004 | 1.91E-10 |
| Chronotype | rs66617308 | 10 | 56699338 | PCDH15,MTRNR2L5 | T | C | 0.018 | 0.003 | 4.40E-08 |
| Chronotype | rs9416744 | 10 | 60567937 | BICC1 | A | C | 0.034 | 0.004 | 7.38E-17 |
| Chronotype | rs12249410 | 10 | 64301941 | ZNF365 | T | G | -0.034 | 0.006 | 2.54E-09 |
| Chronotype | rs17712705 | 10 | 69623271 | DJC12,SIRT1 | A | G | -0.025 | 0.004 | 3.47E-09 |
| Chronotype | rs10762434 | 10 | 73044413 | UNC5B | C | G | 0.025 | 0.004 | 2.20E-08 |
| Chronotype | rs61875203 | 10 | 93888810 | CPEB3 | T | C | 0.026 | 0.004 | 1.97E-09 |
| Chronotype | rs11200159 | 10 | 123553392 | ATE1 | A | C | -0.023 | 0.004 | 4.15E-09 |
| Chronotype | rs9664044 | 10 | 126710791 | CTBP2 | T | C | -0.027 | 0.004 | 2.00E-09 |
| Chronotype | rs10830107 | 10 | 129304075 | DOCK1,NPS | A | G | 0.028 | 0.005 | 4.58E-09 |
| Chronotype | rs76518095 | 10 | 131149976 | GMT | T | C | 0.040 | 0.007 | 7.82E-09 |
| Chronotype | rs12771973 | 10 | 133749294 | PPP2R2D | A | G | -0.022 | 0.004 | 2.16E-08 |
| Chronotype | rs10742179 | 11 | 27650524 | LIN7C,BDNF | A | G | 0.035 | 0.004 | 3.38E-15 |
| Chronotype | rs621421 | 11 | 30405914 | ARL14EP,MPPED2 | T | C | -0.027 | 0.004 | 2.17E-14 |
| Chronotype | rs11032362 | 11 | 33759092 | CD59,FBXO3 | A | G | 0.070 | 0.006 | 1.15E-27 |
| Chronotype | rs7111582 | 11 | 43893222 | HSD17B12,ALKBH3 | A | G | -0.039 | 0.005 | 4.11E-14 |
| Chronotype | rs12808544 | 11 | 58373221 | ZFP91 | A | C | -0.035 | 0.004 | 3.08E-15 |
| Chronotype | rs662094 | 11 | 66342691 | CTSF,CCDC87 | A | G | 0.028 | 0.004 | 5.45E-13 |
| Chronotype | rs1278402 | 11 | 82972097 | ANKRD42,CCDC90B | A | G | 0.028 | 0.005 | 2.40E-09 |
| Chronotype | rs1508608 | 11 | 92893825 | SLC36A4 | A | G | 0.028 | 0.004 | 8.36E-13 |
| Chronotype | rs4121878 | 11 | 95120372 | SESN3,FAM76B | C | G | 0.022 | 0.004 | 8.41E-09 |
| Chronotype | rs17577073 | 11 | 99152801 | CNTN5 | A | C | 0.025 | 0.004 | 2.02E-10 |
| Chronotype | rs2514214 | 11 | 113395329 | DRD2,TMPRSS5 | A | G | 0.027 | 0.005 | 1.98E-09 |
| Chronotype | rs4936290 | 11 | 114009255 | ZBTB16 | A | C | -0.023 | 0.004 | 1.31E-10 |
| Chronotype | rs3867239 | 11 | 122093090 | BLID,UBASH3B | A | G | 0.026 | 0.004 | 1.94E-11 |
| Chronotype | rs74357745 | 11 | 122811822 | C11orf63 | A | G | 0.031 | 0.005 | 3.88E-10 |
| Chronotype | rs7943634 | 11 | 126734319 | KIRREL3 | T | C | -0.024 | 0.004 | 8.55E-10 |
| Chronotype | rs1799464 | 12 | 16286082 | DERA,SLC15A5 | A | G | -0.020 | 0.004 | 2.86E-08 |
| Chronotype | rs12298405 | 12 | 17015267 | LMO3, | T | C | -0.023 | 0.004 | 1.68E-10 |
| Chronotype | rs11611435 | 12 | 24089322 | SOX5 | T | C | 0.028 | 0.004 | 6.62E-11 |
| Chronotype | rs1843888 | 12 | 38737310 | ALG10B,CPNE8 | A | G | 0.051 | 0.004 | 2.36E-43 |
| Chronotype | rs247929 | 12 | 46294908 | ARID2 | C | G | 0.031 | 0.004 | 4.93E-17 |
| Chronotype | rs7299922 | 12 | 54702519 | NFE2,COPZ1 | A | G | 0.024 | 0.004 | 5.38E-11 |
| Chronotype | rs10877962 | 12 | 63520912 | PPM1H,AVPR1A | T | C | 0.036 | 0.004 | 3.70E-18 |
| Chronotype | rs7959983 | 12 | 90452978 | ATP2B1,CCER1 | T | C | -0.030 | 0.004 | 4.54E-16 |
| Chronotype | rs7304278 | 12 | 106989915 | POLR3B,RFX4 | A | G | -0.029 | 0.004 | 1.53E-14 |
| Chronotype | rs7298532 | 12 | 112510404 | A25 | T | C | 0.027 | 0.004 | 1.42E-10 |
| Chronotype | rs80097534 | 12 | 121029604 | POP5,CABP1 | T | G | -0.036 | 0.006 | 2.78E-09 |
| Chronotype | rs9597241 | 13 | 56281271 | / | A | C | 0.033 | 0.004 | 9.39E-14 |
| Chronotype | rs9571526 | 13 | 66590868 | PCDH9 | T | G | -0.027 | 0.005 | 5.14E-09 |
| Chronotype | rs2593487 | 13 | 69903058 | KLHL1 | A | G | -0.029 | 0.004 | 2.03E-12 |
| Chronotype | rs495593 | 13 | 72919800 | DACH1,MZT1 | A | G | 0.023 | 0.004 | 3.67E-09 |
| Chronotype | rs9573980 | 13 | 77590741 | FBXL3 | A | G | 0.127 | 0.010 | 5.03E-36 |
| Chronotype | rs1886205 | 13 | 94062095 | GPC6 | A | C | 0.029 | 0.004 | 6.25E-14 |
| Chronotype | rs9558942 | 13 | 107700218 | ARGLU1,FAM155A | T | C | -0.019 | 0.004 | 4.43E-08 |
| Chronotype | rs3815983 | 13 | 109779906 | MYO16 | T | C | -0.022 | 0.003 | 4.17E-10 |
| Chronotype | rs61990287 | 14 | 42069889 | LRFN5 | A | C | 0.025 | 0.004 | 6.04E-09 |
| Chronotype | rs7143933 | 14 | 62460219 | SPC1,SYT16 | T | G | 0.025 | 0.004 | 6.52E-09 |
| Chronotype | rs2978382 | 14 | 64769074 | ESR2,MTHFD1 | T | C | 0.023 | 0.004 | 6.47E-09 |
| Chronotype | rs4903203 | 14 | 74660508 | LIN52 | A | G | 0.025 | 0.004 | 1.67E-10 |
| Chronotype | rs4550384 | 14 | 85350142 | FLRT2 | T | G | 0.024 | 0.004 | 1.65E-09 |
| Chronotype | rs11845599 | 14 | 101016824 | BEGAIN | A | G | -0.027 | 0.004 | 5.22E-13 |
| Chronotype | rs59986227 | 15 | 48009263 | SEMA6D | C | G | -0.031 | 0.005 | 1.39E-11 |
| Chronotype | rs12442008 | 15 | 53725112 | ONECUT1,WDR72 | T | C | 0.029 | 0.004 | 5.10E-11 |
| Chronotype | rs12442674 | 15 | 96907819 | NR2F2,SPATA8 | A | C | 0.023 | 0.004 | 8.00E-09 |
| Chronotype | rs1873958 | 15 | 101147726 | ASB7 | A | G | 0.028 | 0.004 | 2.39E-15 |
| Chronotype | rs12445235 | 16 | 8195278 | RBFOX1,TMEM114 | C | G | -0.021 | 0.004 | 1.37E-08 |
| Chronotype | rs2304467 | 16 | 8988777 | USP7 | C | G | -0.024 | 0.004 | 6.78E-09 |
| Chronotype | rs11641239 | 16 | 23124193 | USP31 | T | C | 0.023 | 0.004 | 4.65E-09 |
| Chronotype | rs7203707 | 16 | 24518569 | CACNG3,RBBP6 | A | C | -0.020 | 0.003 | 5.00E-10 |
| Chronotype | rs4785296 | 16 | 49467234 | C16orf78,ZNF423 | C | G | 0.026 | 0.004 | 1.37E-09 |
| Chronotype | rs2550298 | 16 | 56367969 | GO1 | T | C | -0.040 | 0.004 | 5.67E-26 |
| Chronotype | rs8044054 | 16 | 60628436 | / | T | C | 0.031 | 0.004 | 7.05E-17 |
| Chronotype | rs72790386 | 16 | 68136932 | NFATC3 | T | G | 0.060 | 0.011 | 2.07E-08 |
| Chronotype | rs1061032 | 17 | 8064083 | VAMP2 | T | G | 0.064 | 0.006 | 1.69E-23 |
| Chronotype | rs12950382 | 17 | 30603994 | RHBDL3 | A | G | 0.023 | 0.004 | 4.70E-08 |
| Chronotype | rs4365329 | 17 | 31625887 | ASIC2 | A | T | -0.019 | 0.003 | 2.68E-08 |
| Chronotype | rs2011528 | 17 | 33980566 | AP2B1 | T | C | -0.033 | 0.005 | 3.17E-11 |
| Chronotype | rs3760381 | 17 | 43047083 | C1QL1,DCAKD | A | G | 0.027 | 0.004 | 3.84E-10 |
| Chronotype | rs12051 | 17 | 46103760 | COPZ2 | A | G | -0.026 | 0.004 | 4.86E-13 |
| Chronotype | rs55846845 | 17 | 50092201 | CA10 | A | G | -0.021 | 0.003 | 4.71E-11 |
| Chronotype | rs72829706 | 17 | 54173733 | PCTP,ANKFN1 | A | G | 0.056 | 0.009 | 1.24E-10 |
| Chronotype | rs8072058 | 17 | 55734198 | MSI2 | A | T | -0.028 | 0.005 | 8.59E-09 |
| Chronotype | rs412000 | 17 | 56709058 | TEX14 | C | G | -0.022 | 0.004 | 1.67E-09 |
| Chronotype | rs72841368 | 17 | 61391114 | TANC2 | A | T | -0.030 | 0.005 | 2.44E-11 |
| Chronotype | rs2916148 | 17 | 65482109 | PITPNC1 | A | G | 0.028 | 0.004 | 3.07E-14 |
| Chronotype | rs11545787 | 17 | 17398278 | RASD1 | A | G | -0.050 | 0.004 | 4.20E-30 |
| Chronotype | rs2580160 | 18 | 1816036 | ADCYAP1,METTL4 | A | G | 0.028 | 0.004 | 3.88E-11 |
| Chronotype | rs62082402 | 18 | 5186566 | C18orf42 | T | G | 0.050 | 0.006 | 6.90E-19 |
| Chronotype | rs1013987 | 18 | 22630836 | HRH4,ZNF521 | T | C | -0.029 | 0.004 | 5.57E-13 |
| Chronotype | rs4419127 | 18 | 31663654 | NOL4 | A | G | 0.044 | 0.004 | 8.00E-30 |
| Chronotype | rs9950528 | 18 | 35762461 | CELF4, | A | G | -0.024 | 0.004 | 4.32E-09 |
| Chronotype | rs12969848 | 18 | 38152835 | / | T | C | 0.036 | 0.004 | 1.06E-21 |
| Chronotype | rs4800998 | 18 | 53429655 | TCF4,TXNL1 | A | T | 0.039 | 0.005 | 4.63E-14 |
| Chronotype | rs11152350 | 18 | 60240352 | ZCCHC2 | A | C | -0.028 | 0.004 | 1.20E-13 |
| Chronotype | rs1025601 | 18 | 73056278 | TSHZ1,SMIM21 | T | C | -0.022 | 0.004 | 2.67E-08 |
| Chronotype | rs10402849 | 19 | 2695661 | GNG7 | T | C | 0.026 | 0.004 | 2.98E-09 |
| Chronotype | rs36055559 | 19 | 5799433 | DUS3L,NRTN | A | G | -0.036 | 0.005 | 7.09E-12 |
| Chronotype | rs7248205 | 19 | 10770305 | ILF3 | T | C | 0.027 | 0.004 | 9.96E-13 |
| Chronotype | rs9636202 | 19 | 18449238 | LSM4,PGPEP1 | A | G | -0.026 | 0.004 | 3.67E-11 |
| Chronotype | rs73026775 | 19 | 31052954 | ZNF536,TSHZ3 | A | G | -0.034 | 0.006 | 2.16E-08 |
| Chronotype | rs58876439 | 19 | 42600984 | POU2F2 | A | G | 0.047 | 0.007 | 3.05E-11 |
| Chronotype | rs11670534 | 19 | 47003906 | PPP5D1 | T | C | -0.031 | 0.005 | 5.46E-10 |
| Chronotype | rs6131805 | 20 | 16222093 | MACROD2,KIF16B | T | G | 0.026 | 0.004 | 1.68E-11 |
| Chronotype | rs6131942 | 20 | 17348608 | PCSK2 | A | G | -0.026 | 0.003 | 7.42E-16 |
| Chronotype | rs1474754 | 20 | 20077178 | C20orf26 | A | G | -0.021 | 0.004 | 1.76E-08 |
| Chronotype | rs6047481 | 20 | 21539564 | NKX22,PAX1 | A | T | 0.025 | 0.004 | 3.40E-09 |
| Chronotype | rs1737893 | 20 | 31051699 | C20orf112 | T | C | -0.025 | 0.004 | 9.49E-11 |
| Chronotype | rs57236847 | 20 | 44668401 | SLC12A5 | C | G | 0.027 | 0.005 | 4.38E-09 |
| Chronotype | rs695459 | 22 | 28848278 | TTC28 | T | C | -0.022 | 0.004 | 1.17E-08 |
| Chronotype | rs28459838 | 22 | 35846168 | MCM5,RASD2 | T | C | 0.027 | 0.004 | 1.30E-09 |
| Chronotype | rs139911 | 22 | 40704052 | TNRC6B | T | C | -0.034 | 0.004 | 1.39E-18 |
| Insomnia | rs10800992 | 1 | 190900576 | RP11-463J7.2 | T | C | 0.042 | 0.006 | 3.84E-12 |
| Insomnia | rs11119409 | 1 | 210293333 | SYT14 | T | C | -0.035 | 0.006 | 1.19E-08 |
| Insomnia | rs11588755 | 1 | 57819204 | DAB1 | A | G | -0.035 | 0.006 | 5.14E-09 |
| Insomnia | rs12030482 | 1 | 96961268 | EEF1A1P11 | A | T | 0.041 | 0.007 | 8.16E-09 |
| Insomnia | rs1289939 | 1 | 117944435 | MAN1A2 | T | C | -0.041 | 0.007 | 6.00E-09 |
| Insomnia | rs1937447 | 1 | 66358242 | PDE4B | C | G | -0.039 | 0.007 | 2.08E-08 |
| Insomnia | rs2089358 | 1 | 37194103 | / | T | C | -0.041 | 0.007 | 2.75E-10 |
| Insomnia | rs623025 | 1 | 201765094 | IPO9-AS1 | T | C | -0.038 | 0.007 | 3.16E-08 |
| Insomnia | rs6702604 | 1 | 107190062 | / | A | G | -0.037 | 0.006 | 1.30E-09 |
| Insomnia | rs10928256 | 2 | 146458738 | / | T | C | 0.034 | 0.006 | 1.61E-08 |
| Insomnia | rs116466468 | 2 | 159137557 | CCDC148 | T | C | 0.044 | 0.007 | 2.11E-10 |
| Insomnia | rs11679943 | 2 | 77724624 | LRRTM4 | A | G | 0.037 | 0.006 | 3.16E-09 |
| Insomnia | rs12991815 | 2 | 68071990 | AC010987.6 | C | G | 0.040 | 0.006 | 3.02E-11 |
| Insomnia | rs1519102 | 2 | 66677816 | MEIS1 | C | G | -0.037 | 0.006 | 1.90E-09 |
| Insomnia | rs1530938 | 2 | 236900633 | AGAP1 | A | G | 0.036 | 0.006 | 8.82E-10 |
| Insomnia | rs55772859 | 2 | 208042581 | / | A | C | 0.042 | 0.006 | 4.82E-11 |
| Insomnia | rs56097173 | 2 | 44262449 | / | T | C | 0.040 | 0.006 | 2.69E-10 |
| Insomnia | rs62213452 | 2 | 210380152 | MAP2 | T | G | 0.037 | 0.007 | 2.39E-08 |
| Insomnia | rs6545798 | 2 | 60521311 | / | A | T | -0.041 | 0.006 | 1.19E-11 |
| Insomnia | rs6756610 | 2 | 147480394 | / | C | G | 0.037 | 0.006 | 1.14E-09 |
| Insomnia | rs75452188 | 2 | 67134426 | AC007403.2 | A | G | 0.052 | 0.009 | 1.58E-08 |
| Insomnia | rs7571486 | 2 | 176473295 | / | A | G | -0.039 | 0.007 | 1.40E-09 |
| Insomnia | rs7599697 | 2 | 239231477 | TRAF3IP1 | T | C | -0.037 | 0.006 | 5.00E-09 |
| Insomnia | rs823247 | 2 | 2850540 | / | T | C | -0.037 | 0.006 | 5.25E-10 |
| Insomnia | rs10865954 | 3 | 49211989 | KLHDC8B | T | C | 0.042 | 0.006 | 1.92E-11 |
| Insomnia | rs1567084 | 3 | 71435955 | FOXP1 | A | G | 0.033 | 0.006 | 2.14E-08 |
| Insomnia | rs17025198 | 3 | 88001713 | HTR1F | A | G | 0.041 | 0.007 | 2.19E-08 |
| Insomnia | rs2216427 | 3 | 180785697 | SOX2-OT | C | G | 0.035 | 0.006 | 1.60E-09 |
| Insomnia | rs2364921 | 3 | 158522463 | MFSD1 | T | C | -0.034 | 0.006 | 2.13E-08 |
| Insomnia | rs35110063 | 3 | 43066558 | KRBOX1:FAM198A | A | G | 0.039 | 0.006 | 8.82E-11 |
| Insomnia | rs3774751 | 3 | 50209053 | SEMA3F | T | G | -0.041 | 0.006 | 7.32E-12 |
| Insomnia | rs62264767 | 3 | 117642005 | LOC107986022 | A | C | 0.065 | 0.008 | 1.63E-14 |
| Insomnia | rs6808140 | 3 | 10581380 | ATP2B2 | T | C | 0.039 | 0.006 | 5.35E-11 |
| Insomnia | rs694786 | 3 | 173112907 | NLGN1 | T | C | -0.044 | 0.006 | 1.97E-13 |
| Insomnia | rs7615602 | 3 | 18718055 | / | C | G | -0.040 | 0.007 | 2.59E-09 |
| Insomnia | rs7625896 | 3 | 44062561 | / | A | G | 0.036 | 0.006 | 5.28E-09 |
| Insomnia | rs11722569 | 4 | 112822731 | LOC105377369 | T | C | 0.034 | 0.006 | 2.91E-08 |
| Insomnia | rs13138995 | 4 | 148987430 | ARHGAP10 | A | G | 0.034 | 0.006 | 1.97E-08 |
| Insomnia | rs16990210 | 4 | 34720226 | / | T | C | -0.046 | 0.008 | 1.97E-08 |
| Insomnia | rs62301574 | 4 | 22050165 | / | C | G | -0.042 | 0.007 | 1.37E-08 |
| Insomnia | rs72657797 | 4 | 90820809 | MMRN1 | T | C | -0.056 | 0.008 | 1.52E-12 |
| Insomnia | rs16903122 | 5 | 87693561 | TMEM161B-AS1 | T | C | 0.055 | 0.007 | 9.04E-16 |
| Insomnia | rs17083297 | 5 | 92995477 | FAM172A | A | C | -0.044 | 0.008 | 1.60E-09 |
| Insomnia | rs17223714 | 5 | 50492629 | RNU6-1296P | A | G | 0.046 | 0.007 | 2.44E-10 |
| Insomnia | rs17367725 | 5 | 107112116 | RN7SKP122 | T | C | -0.036 | 0.006 | 9.29E-09 |
| Insomnia | rs2431108 | 5 | 103947968 | RP11-6N13.1 | T | C | -0.053 | 0.006 | 7.83E-17 |
| Insomnia | rs37445 | 5 | 106899684 | EF5 | A | G | -0.036 | 0.006 | 4.88E-09 |
| Insomnia | rs55972276 | 5 | 135653737 | TRPC7 | A | C | 0.073 | 0.009 | 4.19E-17 |
| Insomnia | rs6601080 | 5 | 179511043 | / | A | G | 0.035 | 0.006 | 2.21E-08 |
| Insomnia | rs6888135 | 5 | 141254063 | PCDH1 | A | C | 0.038 | 0.006 | 1.21E-10 |
| Insomnia | rs701394 | 5 | 80296487 | RASGRF2 | A | G | -0.036 | 0.006 | 6.83E-09 |
| Insomnia | rs8180457 | 5 | 107209814 | FBXL17 | T | C | -0.056 | 0.008 | 1.12E-11 |
| Insomnia | rs10944696 | 6 | 94498850 | SNORA18 | A | G | -0.038 | 0.007 | 7.99E-09 |
| Insomnia | rs10947690 | 6 | 37631768 | MDGA1 | A | G | -0.047 | 0.007 | 4.04E-12 |
| Insomnia | rs10947987 | 6 | 41754370 | PRICKLE4 | T | C | -0.033 | 0.006 | 4.08E-08 |
| Insomnia | rs11756035 | 6 | 18843810 | MIR548A1 | C | G | 0.051 | 0.009 | 1.29E-08 |
| Insomnia | rs2388840 | 6 | 99598756 | BDH2P1 | A | G | -0.037 | 0.006 | 1.37E-09 |
| Insomnia | rs3131638 | 6 | 31475127 | MICB | A | G | -0.044 | 0.007 | 7.88E-10 |
| Insomnia | rs314281 | 6 | 105400605 | LIN28B | T | C | -0.043 | 0.006 | 6.03E-13 |
| Insomnia | rs4709655 | 6 | 163280204 | PACRG | T | C | -0.054 | 0.009 | 3.09E-09 |
| Insomnia | rs728017 | 6 | 124292594 | NKAIN2 | A | G | -0.035 | 0.006 | 9.51E-09 |
| Insomnia | rs9373590 | 6 | 101212001 | ASCC3 | A | T | 0.040 | 0.006 | 2.18E-11 |
| Insomnia | rs9394502 | 6 | 38452503 | BTBD9 | T | C | -0.054 | 0.006 | 7.76E-18 |
| Insomnia | rs12666306 | 7 | 115082406 | / | A | G | 0.042 | 0.006 | 2.24E-12 |
| Insomnia | rs17520265 | 7 | 119674508 | / | A | G | -0.091 | 0.016 | 2.87E-08 |
| Insomnia | rs190073 | 7 | 10985188 | LOC107986767 | A | G | -0.034 | 0.006 | 2.86E-08 |
| Insomnia | rs2598293 | 7 | 133989882 | SLC35B4 | T | C | 0.035 | 0.006 | 2.48E-09 |
| Insomnia | rs521484 | 7 | 49894349 | VWC2 | A | G | -0.040 | 0.007 | 1.53E-08 |
| Insomnia | rs670501 | 7 | 108625185 | / | T | C | 0.053 | 0.007 | 7.40E-13 |
| Insomnia | rs6967168 | 7 | 132672192 | CHCHD3 | T | G | -0.044 | 0.007 | 1.39E-10 |
| Insomnia | rs6978112 | 7 | 1966841 | MAD1L1 | T | C | 0.034 | 0.006 | 2.11E-08 |
| Insomnia | rs73671843 | 7 | 3520024 | SDK1 | A | G | -0.056 | 0.009 | 5.49E-10 |
| Insomnia | rs75932578 | 7 | 106844694 | COG5 | T | C | -0.040 | 0.007 | 4.15E-08 |
| Insomnia | rs8180817 | 7 | 114047542 | FOXP2 | C | G | -0.049 | 0.006 | 1.83E-16 |
| Insomnia | rs10955647 | 8 | 114154187 | CSMD3 | T | G | 0.033 | 0.006 | 1.84E-08 |
| Insomnia | rs17643634 | 8 | 91650818 | TMEM64 | T | C | -0.060 | 0.008 | 1.34E-13 |
| Insomnia | rs2737240 | 8 | 116657235 | TRPS1 | A | G | 0.036 | 0.007 | 3.37E-08 |
| Insomnia | rs28552587 | 8 | 103356226 | UBR5 | A | G | 0.033 | 0.006 | 3.30E-08 |
| Insomnia | rs28611339 | 8 | 10170037 | MSRA | T | G | 0.058 | 0.009 | 8.46E-11 |
| Insomnia | rs4588900 | 8 | 73890425 | / | A | G | 0.033 | 0.006 | 1.57E-08 |
| Insomnia | rs671985 | 8 | 60914783 | / | A | G | -0.038 | 0.006 | 2.79E-10 |
| Insomnia | rs871994 | 8 | 35190619 | UNC5D | A | C | 0.035 | 0.006 | 5.50E-09 |
| Insomnia | rs874168 | 8 | 30849450 | PURG | T | C | 0.034 | 0.006 | 7.95E-09 |
| Insomnia | rs10756571 | 9 | 14534505 | / | T | C | 0.036 | 0.006 | 1.80E-08 |
| Insomnia | rs10758593 | 9 | 4292083 | GLIS3 | A | G | -0.036 | 0.006 | 4.90E-09 |
| Insomnia | rs10761240 | 9 | 96361922 | PHF2 | A | G | -0.043 | 0.006 | 2.12E-12 |
| Insomnia | rs118166957 | 9 | 8858043 | PTPRD | T | C | 0.068 | 0.008 | 1.95E-16 |
| Insomnia | rs1927902 | 9 | 120518991 | / | T | C | 0.053 | 0.007 | 1.15E-14 |
| Insomnia | rs2792990 | 9 | 125621610 | RC3H2 | C | G | 0.054 | 0.008 | 1.15E-10 |
| Insomnia | rs6597649 | 9 | 133786652 | FIBCD1 | T | C | 0.033 | 0.006 | 3.05E-08 |
| Insomnia | rs7044885 | 9 | 81739348 | RP11-165H23.1 | C | G | -0.041 | 0.006 | 5.67E-12 |
| Insomnia | rs72773790 | 9 | 139109080 | QSOX2 | T | C | 0.037 | 0.006 | 3.71E-09 |
| Insomnia | rs10825503 | 10 | 57177470 | PCDH15 | T | G | 0.033 | 0.006 | 1.43E-08 |
| Insomnia | rs11001276 | 10 | 76825638 | DUPD1 | A | T | -0.038 | 0.007 | 2.52E-08 |
| Insomnia | rs224029 | 10 | 64519299 | ALDH7A1P4 | T | C | -0.039 | 0.006 | 2.51E-10 |
| Insomnia | rs7475916 | 10 | 77771194 | C10orf11 | C | G | -0.037 | 0.006 | 6.70E-09 |
| Insomnia | rs10898940 | 11 | 73455292 | RAB6A | A | C | 0.034 | 0.006 | 8.09E-09 |
| Insomnia | rs11605348 | 11 | 47606483 | NDUFS3 | A | G | -0.045 | 0.006 | 7.01E-13 |
| Insomnia | rs12790660 | 11 | 57667222 | OR5AZ1P | T | C | -0.040 | 0.006 | 4.49E-10 |
| Insomnia | rs214934 | 11 | 17193475 | PIK3C2A | A | T | -0.038 | 0.006 | 3.16E-09 |
| Insomnia | rs2221119 | 11 | 88598444 | GRM5 | C | G | 0.036 | 0.006 | 2.00E-09 |
| Insomnia | rs4592425 | 11 | 62697813 | RN7SL259P | T | G | 0.040 | 0.006 | 4.31E-10 |
| Insomnia | rs524859 | 11 | 66041079 | RAB1B,LOC107984340 | A | G | -0.044 | 0.006 | 1.48E-12 |
| Insomnia | rs56133505 | 11 | 72348039 | PDE2A | A | G | 0.041 | 0.006 | 5.59E-12 |
| Insomnia | rs566673 | 11 | 66401373 | RBM14-RBM4:RBM4 | T | G | -0.039 | 0.006 | 1.18E-10 |
| Insomnia | rs667730 | 11 | 83277325 | DLG2 | T | C | 0.033 | 0.006 | 2.26E-08 |
| Insomnia | rs72899452 | 11 | 45415577 | / | T | C | 0.074 | 0.012 | 1.00E-09 |
| Insomnia | rs12310246 | 12 | 84700945 | / | A | G | 0.045 | 0.007 | 4.74E-11 |
| Insomnia | rs2286729 | 12 | 6873818 | MLF2 | A | G | 0.070 | 0.011 | 5.37E-11 |
| Insomnia | rs28582096 | 12 | 123856998 | hsa-mir-8072 | A | G | -0.054 | 0.007 | 1.74E-13 |
| Insomnia | rs4767645 | 12 | 118385788 | KSR2 | T | G | -0.037 | 0.006 | 6.47E-10 |
| Insomnia | rs61921611 | 12 | 66367726 | HMGA2 | T | C | -0.044 | 0.006 | 7.84E-12 |
| Insomnia | rs6606731 | 12 | 109982578 | UBE3B | A | T | 0.043 | 0.008 | 1.51E-08 |
| Insomnia | rs1031654 | 13 | 54382035 | LINC00558 | A | C | -0.051 | 0.007 | 3.88E-12 |
| Insomnia | rs11149313 | 13 | 85294881 | LINC00333 | A | G | 0.040 | 0.007 | 2.38E-09 |
| Insomnia | rs2389631 | 13 | 96932868 | HS6ST3 | A | C | -0.040 | 0.006 | 2.03E-10 |
| Insomnia | rs6562066 | 13 | 60532796 | DIAPH3 | T | C | 0.039 | 0.006 | 1.38E-10 |
| Insomnia | rs7992992 | 13 | 54721699 | LINC00458 | A | G | 0.051 | 0.009 | 1.15E-08 |
| Insomnia | rs9527083 | 13 | 53991125 | / | A | G | -0.076 | 0.006 | 1.61E-32 |
| Insomnia | rs9540729 | 13 | 66947124 | PCDH9 | A | T | 0.036 | 0.006 | 1.40E-09 |
| Insomnia | rs9563886 | 13 | 61720066 | MIR3169 | T | C | -0.034 | 0.006 | 3.08E-08 |
| Insomnia | rs12912299 | 15 | 38897857 | RASGRP1 | T | C | -0.043 | 0.006 | 4.42E-13 |
| Insomnia | rs12917449 | 15 | 74331659 | PML | A | C | -0.042 | 0.008 | 2.97E-08 |
| Insomnia | rs715338 | 15 | 57215867 | TCF12 | A | G | 0.041 | 0.006 | 7.85E-12 |
| Insomnia | rs7168238 | 15 | 66709386 | MAP2K1 | C | G | 0.064 | 0.011 | 1.80E-08 |
| Insomnia | rs7402939 | 15 | 99183876 | / | T | C | -0.036 | 0.006 | 5.19E-09 |
| Insomnia | rs3184470 | 16 | 715164 | WDR90 | A | G | -0.038 | 0.006 | 9.73E-10 |
| Insomnia | rs34214423 | 16 | 52303107 | CASC22,LOC105371261 | A | C | 0.045 | 0.008 | 3.18E-09 |
| Insomnia | rs35322724 | 16 | 77137324 | MON1B | A | C | 0.049 | 0.006 | 3.75E-16 |
| Insomnia | rs3902952 | 16 | 61647589 | CDH8 | T | C | 0.048 | 0.008 | 2.55E-10 |
| Insomnia | rs4238755 | 16 | 52746089 | / | A | C | -0.043 | 0.007 | 2.30E-10 |
| Insomnia | rs4788203 | 16 | 29978827 | TMEM219 | A | G | -0.035 | 0.006 | 6.32E-09 |
| Insomnia | rs830716 | 16 | 12323509 | SNX29 | C | G | 0.045 | 0.007 | 8.68E-12 |
| Insomnia | rs9931543 | 16 | 56128782 | RP11-461O7.1 | T | C | 0.048 | 0.007 | 1.11E-12 |
| Insomnia | rs11650304 | 17 | 46035001 | PRR15L | C | G | 0.067 | 0.012 | 1.23E-08 |
| Insomnia | rs34490907 | 17 | 26933741 | SPAG5-AS1 | C | G | 0.054 | 0.009 | 1.76E-08 |
| Insomnia | rs4643373 | 17 | 47123423 | IGF2BP1 | T | C | 0.041 | 0.007 | 1.58E-10 |
| Insomnia | rs62068188 | 17 | 2400876 | METTL16 | T | C | 0.049 | 0.008 | 1.18E-09 |
| Insomnia | rs7214267 | 17 | 43157709 | NMT1 | A | G | -0.044 | 0.006 | 5.09E-13 |
| Insomnia | rs8076183 | 17 | 61024696 | / | T | C | -0.038 | 0.006 | 2.75E-10 |
| Insomnia | rs9889282 | 17 | 50259142 | CA10 | A | C | -0.042 | 0.006 | 4.70E-12 |
| Insomnia | rs60565673 | 18 | 52906830 | TCF4 | T | G | -0.043 | 0.006 | 1.59E-12 |
| Insomnia | rs12983032 | 19 | 5073447 | KDM4B | A | G | -0.043 | 0.006 | 1.07E-11 |
| Insomnia | rs6510033 | 19 | 30710785 | / | A | G | -0.037 | 0.007 | 4.66E-08 |
| Insomnia | rs908668 | 19 | 56134038 | ZNF784 | T | C | 0.050 | 0.007 | 1.41E-11 |
| Insomnia | rs2867690 | 20 | 41972028 | SCAR15 | T | C | 0.042 | 0.008 | 3.70E-08 |
| Insomnia | rs6019663 | 20 | 47774512 | STAU1 | T | C | 0.040 | 0.007 | 6.47E-10 |
| Insomnia | rs742760 | 20 | 50985290 | RP4-723E3.1 | A | T | 0.043 | 0.008 | 2.48E-08 |
| Insomnia | rs76145129 | 20 | 62670427 | LINC00176 | T | G | -0.050 | 0.009 | 2.73E-08 |
| Insomnia | rs2838787 | 21 | 46539725 | ADARB1 | A | G | -0.036 | 0.006 | 7.65E-09 |
| Sleep duration | rs12567114 | 1 | 98527951 | DPYD | A | G | 0.890 | 0.152 | 4.30E-09 |
| Sleep duration | rs269054 | 1 | 57864304 | DAB1 | A | T | 0.819 | 0.138 | 2.10E-09 |
| Sleep duration | rs61796569 | 1 | 66476437 | PDE4B | T | C | 0.927 | 0.154 | 1.50E-09 |
| Sleep duration | rs915416 | 1 | 34731984 | CSMD2, C1orf94, GJB5 | C | G | 1.156 | 0.150 | 9.90E-15 |
| Sleep duration | rs10173260 | 2 | 210377845 | MAP2 | C | T | 0.770 | 0.139 | 2.90E-08 |
| Sleep duration | rs11885663 | 2 | 166944004 | SCN1A, GALNT3, TTC21B | T | C | 0.973 | 0.157 | 8.60E-10 |
| Sleep duration | rs12611523 | 2 | 139195328 | SPOPL | A | G | 0.758 | 0.137 | 3.10E-08 |
| Sleep duration | rs374153 | 2 | 40382712 | SLC8A1 | C | T | 1.057 | 0.186 | 9.10E-09 |
| Sleep duration | rs4128364 | 2 | 147612734 | PABPCP2, LOC100133235 | C | T | 0.876 | 0.143 | 1.40E-09 |
| Sleep duration | rs4538155 | 2 | 157040773 | NR4A2, GPD2 | T | C | 0.779 | 0.142 | 3.60E-08 |
| Sleep duration | rs62120041 | 2 | 9185564 | MBOAT2 | T | C | 1.567 | 0.274 | 9.60E-09 |
| Sleep duration | rs72804080 | 2 | 59358659 | LINC01122, LOC101927285 | G | A | 1.068 | 0.192 | 2.90E-08 |
| Sleep duration | rs75539574 | 2 | 58871658 | VRK2, LINC01122 | C | A | 2.175 | 0.244 | 6.90E-19 |
| Sleep duration | rs7556815 | 2 | 114085785 | PAX8, LOC100130100 | A | G | 2.443 | 0.164 | 1.30E-49 |
| Sleep duration | rs112230981 | 3 | 55879269 | ERC2 | A | G | 1.892 | 0.314 | 2.20E-09 |
| Sleep duration | rs13088093 | 3 | 135838598 | PPP2R3A, PCCB, STAG1, MSL2 | G | T | 0.976 | 0.144 | 7.00E-12 |
| Sleep duration | rs17732997 | 3 | 70470834 | FOXP1, LOC100128160 | C | G | 0.776 | 0.137 | 1.20E-08 |
| Sleep duration | rs7644809 | 3 | 107564459 | BBX, LOC285205 | T | C | 0.784 | 0.138 | 1.60E-08 |
| Sleep duration | rs13109404 | 4 | 102896591 | BANK1 | T | G | 1.872 | 0.264 | 1.40E-12 |
| Sleep duration | rs17427571 | 4 | 82254908 | PRKG2 | A | G | 0.830 | 0.146 | 1.30E-08 |
| Sleep duration | rs2192528 | 4 | 18327896 | LCORL, LOC645174 | A | G | 0.802 | 0.136 | 2.70E-09 |
| Sleep duration | rs35531607 | 4 | 92533225 | CCSER1 | C | T | 0.770 | 0.136 | 1.50E-08 |
| Sleep duration | rs11567976 | 5 | 137654218 | CDC25C, EGR1, ETF1, GFRA3, HSPA9, NME5, CDC23, KIF20A, BRD8, FAM53C, REEP2, KDM3B | T | C | 0.768 | 0.137 | 2.10E-08 |
| Sleep duration | rs151014368 | 5 | 176751059 | LMAN2, FGFR4, SLC34A1, RGS14, PRELID1, NSD1, MXD3 | A | G | 0.966 | 0.169 | 9.10E-09 |
| Sleep duration | rs365663 | 5 | 1428883 | SLC6A3 | A | G | 0.878 | 0.137 | 1.00E-10 |
| Sleep duration | rs460692 | 5 | 3126584 | LINC01377, LOC100132531, LOC285577 | C | T | 1.263 | 0.200 | 3.60E-10 |
| Sleep duration | rs56372231 | 5 | 102321905 | PAM, PPIP5K2, GIN1, C5ORF30 | T | C | 1.017 | 0.144 | 2.20E-12 |
| Sleep duration | rs113113059 | 6 | 43160375 | CUL9, MEA1, PPP2R5D, SMAD5-AS2, SRF, CUL7, DNPH1, MRPL2, TTBK1, RRP36, KLC4, KLHDC3 | T | C | 0.968 | 0.164 | 8.40E-09 |
| Sleep duration | rs2231265 | 6 | 89790201 | PNRC1 | G | A | 0.897 | 0.162 | 2.70E-08 |
| Sleep duration | rs34556183 | 6 | 28584775 | ZBED9,SCAND3, LOC646160, | A | G | 1.015 | 0.151 | 2.30E-11 |
| Sleep duration | rs80193650 | 6 | 33464363 | ZBTB9, KIFC1, PHF1, SYNGAP1, CUTA | G | A | 1.010 | 0.184 | 4.10E-08 |
| Sleep duration | rs9345234 | 6 | 93162639 | LOC100129847, LOC100128159 | C | A | 0.781 | 0.138 | 1.80E-08 |
| Sleep duration | rs9382445 | 6 | 54937974 | FAM83B | T | C | 0.872 | 0.140 | 4.80E-10 |
| Sleep duration | rs2079070 | 7 | 114126432 | FOXP2 | C | G | 1.053 | 0.154 | 7.50E-12 |
| Sleep duration | rs34731055 | 7 | 2106928 | MAD1L1 | T | C | 1.168 | 0.177 | 3.70E-11 |
| Sleep duration | rs7806045 | 7 | 132610266 | CHCHD3 | T | C | 0.887 | 0.158 | 1.40E-08 |
| Sleep duration | rs330088 | 8 | 9149746 | PPP1R3B, LOC100129150 | C | T | 0.868 | 0.137 | 2.70E-10 |
| Sleep duration | rs73219758 | 8 | 14279446 | SGCZ | G | A | 0.984 | 0.150 | 5.60E-11 |
| Sleep duration | rs10973207 | 9 | 37100525 | EBLN3, ZCCHC7 | T | G | 1.226 | 0.187 | 6.00E-11 |
| Sleep duration | rs1776776 | 9 | 140497072 | ARRDC1, MRPL41, EHMT1, ARRDC1-AS1, DPH7, ZMYND19 | T | C | 1.198 | 0.205 | 4.90E-09 |
| Sleep duration | rs10761674 | 10 | 64618340 | EGR2, ADO | C | T | 0.740 | 0.136 | 4.20E-08 |
| Sleep duration | rs12246842 | 10 | 21830580 | MLLT10, DJC1, SKIDA1 | A | G | 0.804 | 0.136 | 3.90E-09 |
| Sleep duration | rs7915425 | 10 | 125016501 | BUB3, LOC100131719, GPR26 | T | C | 1.144 | 0.179 | 2.00E-10 |
| Sleep duration | rs1057703 | 11 | 122830251 | BSX, C11orf63 | G | T | 1.164 | 0.192 | 1.10E-09 |
| Sleep duration | rs11602180 | 11 | 48162453 | PTPRJ, OR4X2, OR4B1, OR4S1, OR4X1 | C | T | 1.095 | 0.184 | 2.30E-09 |
| Sleep duration | rs1263056 | 11 | 116576415 | BUD13 | A | G | 0.768 | 0.137 | 2.00E-08 |
| Sleep duration | rs1517572 | 11 | 28829882 | METT5D1, OR2BH1P | C | A | 0.879 | 0.138 | 1.50E-10 |
| Sleep duration | rs1553132 | 11 | 88297740 | GRM5 | G | A | 0.870 | 0.155 | 2.50E-08 |
| Sleep duration | rs1939455 | 11 | 101520886 | TRPC6 | G | T | 1.226 | 0.214 | 1.20E-08 |
| Sleep duration | rs4592416 | 11 | 43800474 | HSD17B12 | G | A | 0.881 | 0.136 | 9.30E-11 |
| Sleep duration | rs7115226 | 11 | 113408518 | DRD2 | A | C | 1.594 | 0.261 | 1.70E-09 |
| Sleep duration | rs7951019 | 11 | 118358027 | KMT2A,ARCN1, IFT46, TMEM25 | G | T | 2.213 | 0.391 | 1.20E-08 |
| Sleep duration | rs34354917 | 12 | 38764559 | ALG10B, CPNE8 | C | A | 0.825 | 0.150 | 3.90E-08 |
| Sleep duration | rs4767550 | 12 | 117951150 | KSR2 | G | A | 0.858 | 0.139 | 6.30E-10 |
| Sleep duration | rs10483350 | 14 | 29816155 | MIR548AI, LOC100128215, PRKD1 | G | A | 1.042 | 0.172 | 1.50E-09 |
| Sleep duration | rs11621908 | 14 | 78495761 | ADCK1, FRDAP | C | T | 1.446 | 0.250 | 5.60E-09 |
| Sleep duration | rs55658675 | 14 | 65554638 | MAX, FNTB, GPX2, CHURC1, RAB15 | C | T | 0.788 | 0.142 | 2.00E-08 |
| Sleep duration | rs61985058 | 14 | 60233841 | RTN1 | T | C | 1.116 | 0.194 | 1.30E-08 |
| Sleep duration | rs6575005 | 14 | 26954078 | NOVA1 | T | C | 0.934 | 0.159 | 4.40E-09 |
| Sleep duration | rs8038326 | 15 | 47989799 | SEMA6D | A | G | 0.955 | 0.152 | 2.80E-10 |
| Sleep duration | rs11643715 | 16 | 23909538 | PRKCB | G | C | 0.834 | 0.150 | 3.20E-08 |
| Sleep duration | rs3095508 | 16 | 6550400 | RBFOX1 | C | A | 0.921 | 0.138 | 3.10E-11 |
| Sleep duration | rs8050478 | 16 | 56120461 | GO1 | G | A | 0.960 | 0.136 | 1.70E-12 |
| Sleep duration | rs9940646 | 16 | 53800629 | FTO | C | G | 1.017 | 0.137 | 1.20E-13 |
| Sleep duration | rs205024 | 17 | 11227352 | SHISA6 | T | C | 0.830 | 0.140 | 3.90E-09 |
| Sleep duration | rs7503199 | 17 | 8134275 | PER1, PFAS, SMAD5-AS5, VAMP2, AURKB, ARHGEF15, RANGRF, BORCS6, CTC1, TMEM107, KRBA2, SLC25A35 | C | T | 0.885 | 0.154 | 1.00E-08 |
| Sleep duration | rs9903973 | 17 | 50571227 | CA10, LOC339209 | C | T | 0.766 | 0.136 | 2.60E-08 |
| Sleep duration | rs12607679 | 18 | 53059748 | TCF4 | T | C | 1.208 | 0.156 | 8.30E-15 |
| Sleep duration | rs10421649 | 19 | 9942262 | PIN1, OLFM2 | A | T | 0.798 | 0.138 | 6.90E-09 |
| Abbreviations: Chr, chromosome; SE, standard error; SNP, single nucleotide polymorphism. | | | | | | | | | |

**Supplementary Table 5. Characteristics of instrumental variables for psychiatric disorders used in the bidirectional Mendelian randomization.**

| Trait | SNP | Chr | Position | Closest gene | Effect allele | Other allele | β | SE | *P* value |
| --- | --- | --- | --- | --- | --- | --- | --- | --- | --- |
| ADHD | rs11420276 | 1 | 44184192 | ST3GAL3 | G | GT | 0.107 | 0.015 | 6.45E-13 |
| ADHD | rs1222063 | 1 | 96602440 |  | A | G | 0.096 | 0.017 | 3.07E-08 |
| ADHD | rs4636402 | 1 | 73916016 |  | C | G | 0.062 | 0.014 | 4.53E-06 |
| ADHD | rs11682489 | 2 | 145739217 | TEX41 | T | C | 0.066 | 0.014 | 3.14E-06 |
| ADHD | rs13023832 | 2 | 215219808 | SPAG16 | A | G | 0.115 | 0.022 | 9.33E-08 |
| ADHD | rs17636214 | 2 | 178877989 | PDE11A | A | G | -0.197 | 0.041 | 2.07E-06 |
| ADHD | rs55748262 | 2 | 10982635 |  | A | G | -0.085 | 0.018 | 2.94E-06 |
| ADHD | rs756354 | 2 | 78840319 |  | C | G | -0.072 | 0.014 | 3.39E-07 |
| ADHD | rs4858241 | 3 | 20669071 |  | T | G | 0.082 | 0.014 | 8.17E-09 |
| ADHD | rs4894783 | 3 | 171626047 | LOC105374218 | A | T | -0.066 | 0.014 | 2.58E-06 |
| ADHD | rs62259516 | 3 | 82773696 |  | T | C | 0.141 | 0.029 | 8.29E-07 |
| ADHD | rs62262061 | 3 | 49917686 |  | A | G | 0.079 | 0.016 | 1.07E-06 |
| ADHD | rs7634587 | 3 | 107516847 | BBX | A | G | 0.065 | 0.014 | 2.20E-06 |
| ADHD | rs1272878 | 4 | 2783049 |  | T | C | 0.091 | 0.018 | 2.83E-07 |
| ADHD | rs28411770 | 4 | 31151456 |  | T | C | 0.086 | 0.015 | 1.15E-08 |
| ADHD | rs4586908 | 4 | 153093640 | LOC105377490 | C | G | -0.062 | 0.014 | 4.90E-06 |
| ADHD | rs72616363 | 4 | 130923603 |  | A | T | 0.239 | 0.051 | 2.74E-06 |
| ADHD | rs77216804 | 4 | 112227986 |  | A | T | 0.104 | 0.022 | 1.53E-06 |
| ADHD | rs1077612 | 5 | 173003455 |  | T | C | -0.076 | 0.017 | 4.65E-06 |
| ADHD | rs185160613 | 5 | 3233586 |  | T | C | -0.195 | 0.042 | 4.04E-06 |
| ADHD | rs4839923 | 6 | 98274701 |  | A | G | 0.065 | 0.014 | 1.90E-06 |
| ADHD | rs6933023 | 6 | 70857743 | COL19A1 | T | C | 0.069 | 0.014 | 4.06E-07 |
| ADHD | rs10262192 | 7 | 114091753 | FOXP2 | A | G | 0.074 | 0.014 | 3.66E-08 |
| ADHD | rs117624174 | 7 | 2081425 | MAD1L1 | T | C | -0.142 | 0.031 | 3.08E-06 |
| ADHD | rs180822580 | 7 | 29825324 |  | A | G | -0.157 | 0.033 | 1.29E-06 |
| ADHD | rs28452470 | 7 | 121957582 |  | A | T | 0.074 | 0.014 | 1.89E-07 |
| ADHD | rs1431592 | 8 | 64322977 |  | T | C | 0.068 | 0.015 | 3.63E-06 |
| ADHD | rs5893200 | 8 | 93389399 |  | G | GT | 0.074 | 0.015 | 1.14E-06 |
| ADHD | rs7459616 | 8 | 786553 | DLGAP2 | C | G | 0.065 | 0.014 | 2.07E-06 |
| ADHD | rs10965173 | 9 | 2197193 | LOC107987043 | A | G | 0.079 | 0.017 | 2.28E-06 |
| ADHD | rs143912172 | 9 | 78461648 |  | A | C | 0.138 | 0.029 | 1.88E-06 |
| ADHD | rs78305268 | 9 | 35949778 |  | A | G | 0.212 | 0.046 | 3.91E-06 |
| ADHD | rs7087891 | 10 | 8792629 |  | A | T | 0.070 | 0.014 | 5.22E-07 |
| ADHD | rs80153782 | 10 | 8539223 |  | A | T | -0.092 | 0.020 | 3.66E-06 |
| ADHD | rs1255536 | 11 | 95318585 |  | A | G | 0.071 | 0.015 | 3.68E-06 |
| ADHD | rs1791794 | 11 | 61437088 |  | A | G | -0.069 | 0.015 | 2.45E-06 |
| ADHD | rs28633403 | 11 | 813264 | RPLP2 | A | G | 0.072 | 0.014 | 4.46E-07 |
| ADHD | rs7930199 | 11 | 25701663 |  | T | C | -0.174 | 0.036 | 1.40E-06 |
| ADHD | rs7330802 | 13 | 78837721 | OBI1-AS1 | A | G | 0.070 | 0.014 | 5.07E-07 |
| ADHD | rs9531784 | 13 | 85982098 | LINC00351 | T | C | 0.085 | 0.017 | 1.05E-06 |
| ADHD | rs9553291 | 13 | 24978452 |  | T | C | 0.119 | 0.025 | 1.36E-06 |
| ADHD | rs9596789 | 13 | 53924726 |  | C | G | -0.157 | 0.034 | 4.42E-06 |
| ADHD | rs1959848 | 14 | 63045670 |  | T | C | -0.070 | 0.014 | 3.69E-07 |
| ADHD | rs60798171 | 15 | 87775903 |  | T | G | -0.078 | 0.016 | 7.25E-07 |
| ADHD | rs1428102 | 16 | 18026440 | LOC107984893 | A | G | 0.065 | 0.014 | 2.98E-06 |
| ADHD | rs8058358 | 16 | 10360376 |  | T | C | -0.067 | 0.014 | 2.77E-06 |
| ADHD | rs17084312 | 18 | 69031589 |  | T | C | 0.179 | 0.034 | 1.60E-07 |
| ADHD | rs4144756 | 18 | 39305154 |  | A | G | 0.077 | 0.015 | 1.46E-07 |
| ADHD | rs11698378 | 20 | 35803101 | MROH8 | T | C | -0.085 | 0.018 | 1.58E-06 |
| ADHD | rs2144782 | 20 | 21176604 |  | T | C | 0.071 | 0.015 | 1.33E-06 |
| ADHD | rs6063848 | 20 | 51238004 | LINC01524, LOC105372666 | T | G | 0.077 | 0.015 | 3.97E-07 |
| ADHD | rs2835344 | 21 | 37801345 | LOC105369301 | T | C | -0.073 | 0.016 | 3.76E-06 |
| ADHD | rs7277470 | 21 | 42368132 |  | A | T | 0.065 | 0.014 | 2.70E-06 |
| ADHD | rs992936 | 21 | 18417022 |  | T | C | 0.072 | 0.014 | 1.78E-07 |
| ADHD | rs5771783 | 22 | 49155329 |  | T | C | 0.064 | 0.014 | 4.26E-06 |
| ASD | rs10780122 | 5 | 163569501 |  | T | C | -0.236 | 0.046 | 2.83E-07 |
| ASD | rs1861059 | 5 | 57080169 |  | T | C | 0.161 | 0.033 | 1.19E-06 |
| ASD | rs2235392 | 6 | 11764061 | ADTRP | A | G | -0.123 | 0.026 | 2.16E-06 |
| ASD | rs12534801 | 7 | 133053154 | EXOC4 | A | C | -0.142 | 0.030 | 2.21E-06 |
| ASD | rs62441548 | 7 | 4992306 | MMD2 | A | G | 0.195 | 0.043 | 4.90E-06 |
| ASD | rs7026354 | 9 | 119184126 |  | A | G | 0.137 | 0.025 | 4.99E-08 |
| ASD | rs66847009 | 12 | 3982482 | PARP11 | T | C | -0.207 | 0.045 | 3.60E-06 |
| ASD | rs112096205 | 13 | 70915010 |  | A | G | 0.224 | 0.048 | 2.41E-06 |
| ASD | rs198540 | 17 | 48643801 | CACNA1G | T | C | 0.138 | 0.029 | 2.42E-06 |
| BD | rs7544145 | 1 | 150138699 | LOC105371433 | T | C | 0.082 | 0.015 | 4.83E-08 |
| BD | rs57195239 | 2 | 97376407 | LMAN2L | I | D | -0.071 | 0.012 | 3.77E-09 |
| BD | rs17183814 | 2 | 166152389 | SCN2A | A | G | -0.133 | 0.022 | 2.02E-09 |
| BD | rs3804640 | 3 | 107793709 | CD47 | A | G | 0.063 | 0.011 | 1.99E-08 |
| BD | rs11724116 | 4 | 162294038 | / | T | C | -0.085 | 0.015 | 2.37E-08 |
| BD | rs10035291 | 5 | 80796368 | SSBP2 | T | C | 0.068 | 0.012 | 2.67E-08 |
| BD | rs10455979 | 6 | 166995260 | RPS6KA2 | C | G | -0.062 | 0.011 | 4.31E-08 |
| BD | rs73188321 | 7 | 105048158 | / | T | C | -0.078 | 0.013 | 1.06E-09 |
| BD | rs10994318 | 10 | 62125856 | ANK3 | C | G | 0.135 | 0.023 | 6.76E-09 |
| BD | rs10896090 | 11 | 65945186 | PACS1 | A | G | 0.08 | 0.014 | 1.90E-08 |
| BD | rs12575685 | 11 | 70517927 | SHANK2 | A | G | 0.07 | 0.012 | 7.71E-09 |
| BD | rs10744560 | 12 | 2387099 | CACNA1C | T | C | 0.073 | 0.012 | 3.62E-10 |
| BD | rs4447398 | 15 | 42904904 | STARD9 | A | C | 0.094 | 0.016 | 9.37E-09 |
| BD | rs139221256 | 15 | 85357857 | / | I | D | -0.07 | 0.013 | 2.67E-08 |
| BD | rs11647445 | 16 | 9926966 | GRIN2A | T | G | -0.076 | 0.012 | 1.08E-10 |
| BD | rs112114764 | 17 | 42201041 | HDAC5 | T | G | -0.068 | 0.012 | 2.45E-08 |
| BD | rs11557713 | 18 | 60243876 | ZCCHC2 | A | G | 0.067 | 0.012 | 3.64E-08 |
| BD | rs111444407 | 19 | 19358207 | NCAN | T | C | 0.093 | 0.015 | 1.33E-09 |
| MDD | rs1432639 | 1 | 72813218 | RERE | A | C | 0.039 | 0.005 | 4.60E-15 |
| MDD | rs2389016 | 1 | 80799329 | / | T | C | 0.03 | 0.005 | 1.00E-08 |
| MDD | rs4261101 | 1 | 90796053 | / | A | G | -0.03 | 0.005 | 1.00E-08 |
| MDD | rs9402472 | 6 | 99566521 | / | A | G | 0.03 | 0.006 | 2.80E-08 |
| MDD | rs12666117 | 7 | 109105611 | / | A | G | 0.03 | 0.005 | 1.40E-08 |
| MDD | rs1354115 | 9 | 2983774 | / | A | C | 0.03 | 0.005 | 2.40E-08 |
| MDD | rs10959913 | 9 | 11544964 | / | T | G | 0.03 | 0.006 | 5.10E-09 |
| MDD | rs7856424 | 9 | 119733595 | ASTN2 | T | C | -0.03 | 0.005 | 8.50E-09 |
| MDD | rs4904738 | 14 | 42179732 | LRFN5 | T | C | -0.03 | 0.005 | 2.60E-09 |
| MDD | rs8025231 | 15 | 37648402 | / | A | C | -0.03 | 0.005 | 2.40E-12 |
| MDD | rs7198928 | 16 | 7,666,402 | RBFOX1 | T | C | 0.03 | 0.005 | 1.00E-08 |
| MDD | rs17727765 | 17 | 27576962 | CRYBA1 | T | C | -0.051 | 0.009 | 8.50E-09 |
| OCD | rs116347760 | 1 | 114201251 | MAGI3 | A | T | 0.630 | 0.134 | 2.39E-06 |
| OCD | rs56343802 | 2 | 139823241 |  | A | T | -0.170 | 0.037 | 3.97E-06 |
| OCD | rs1030757 | 4 | 93697153 | GRID2 | A | C | -0.165 | 0.034 | 1.09E-06 |
| OCD | rs13141765 | 4 | 6243646 |  | T | C | -0.266 | 0.056 | 1.86E-06 |
| OCD | rs55687617 | 7 | 56775429 |  | A | G | -0.270 | 0.058 | 2.67E-06 |
| OCD | rs4733767 | 8 | 128581578 |  | A | G | 0.194 | 0.039 | 7.10E-07 |
| OCD | rs72781967 | 10 | 5622426 |  | T | C | -0.167 | 0.035 | 2.43E-06 |
| OCD | rs639560 | 15 | 42381240 | PLA2G4D | T | C | -0.416 | 0.088 | 2.23E-06 |
| OCD | rs72783425 | 16 | 14148431 |  | A | C | 0.340 | 0.073 | 3.53E-06 |
| OCD | rs117310268 | 18 | 19675267 |  | T | C | 0.449 | 0.097 | 3.31E-06 |
| OCD | rs77885126 | 18 | 58420429 |  | T | C | -0.603 | 0.131 | 4.38E-06 |
| OCD | rs9952159 | 18 | 3660801 | DLGAP1, LOC107985137 | T | C | 0.182 | 0.040 | 4.21E-06 |
| OCD | rs909701 | 22 | 44973368 |  | C | G | -0.155 | 0.034 | 4.05E-06 |
| PTSD | rs2163050 | 2 | 41826738 |  | A | G | 0.100 | 0.021 | 2.12E-06 |
| PTSD | rs1444764 | 3 | 123929043 | KALRN | A | G | 0.078 | 0.016 | 1.90E-06 |
| PTSD | rs139591016 | 4 | 99816170 | EIF4E | T | C | -0.249 | 0.053 | 2.86E-06 |
| PTSD | rs111771848 | 5 | 25997223 |  | G | GA | -0.355 | 0.075 | 2.18E-06 |
| PTSD | rs140928208 | 5 | 15586134 | FBXL7 | A | G | 0.447 | 0.093 | 1.46E-06 |
| PTSD | rs12528870 | 6 | 166197276 | PDE10A | A | G | 0.176 | 0.038 | 2.69E-06 |
| PTSD | rs34517852 | 6 | 157789333 |  | A | T | 0.109 | 0.019 | 3.16E-09 |
| PTSD | rs9364611 | 6 | 162163506 | PRKN | T | C | -0.124 | 0.023 | 4.36E-08 |
| PTSD | rs12706983 | 7 | 131337495 |  | T | G | -0.090 | 0.019 | 3.01E-06 |
| PTSD | rs7807190 | 7 | 1851461 |  | A | G | 0.083 | 0.018 | 4.04E-06 |
| PTSD | rs62583551 | 9 | 138660237 | KCNT1 | A | G | -0.097 | 0.020 | 1.22E-06 |
| PTSD | rs80074987 | 10 | 28323804 |  | T | G | 0.238 | 0.051 | 2.64E-06 |
| PTSD | rs149509653 | 11 | 118558425 |  | A | G | 0.090 | 0.019 | 1.74E-06 |
| PTSD | rs77537694 | 11 | 132725161 | OPCML | A | G | 0.130 | 0.028 | 3.25E-06 |
| PTSD | rs17041470 | 12 | 102430430 | WASHC3 | A | G | -0.298 | 0.064 | 3.66E-06 |
| PTSD | rs4078035 | 12 | 131887167 |  | T | G | 0.173 | 0.035 | 1.10E-06 |
| PTSD | rs57753395 | 13 | 99412436 | LOC105370330 | A | G | 0.376 | 0.071 | 9.13E-08 |
| PTSD | rs17108326 | 14 | 72628507 | RGS6 | A | G | 0.106 | 0.021 | 3.91E-07 |
| PTSD | rs78608260 | 19 | 53637559 | ZNF415 | A | G | -0.321 | 0.069 | 3.71E-06 |
| PTSD | rs35334143 | 20 | 47530708 |  | C | G | -0.080 | 0.017 | 3.15E-06 |
| PTSD | rs73154700 | 22 | 31266823 | OSBP2 | A | G | 0.126 | 0.027 | 3.45E-06 |
| SCZ | rs12129719 | 1 | 66324512 | PDE4B | G | A | -0.054 | 0.01 | 3.35E-08 |
| SCZ | rs6678676 | 1 | 200266503 | LINC00862, ZNF281 | T | C | -0.065 | 0.012 | 3.22E-08 |
| SCZ | rs2970610 | 1 | 44097530 | ARTN, ATP6V0B, B4GALT2, CCDC24, CDC20, DPH2, ELOVL1, HYI, IPO13, KDM4A, KDM4A-AS1, MED8, MIR6079, MIR6734, MIR6735, MPL, PTPRF, SLC6A9, ST3GAL3, SZT2 | T | C | 0.068 | 0.01 | 1.39E-11 |
| SCZ | rs4648845 | 1 | 2387101 | PLCH2 | C | T | -0.077 | 0.011 | 6.74E-12 |
| SCZ | rs28374258 | 1 | 190949551 | LOC440704 | T | A | -0.074 | 0.012 | 6.35E-10 |
| SCZ | rs72769124 | 1 | 239210058 | / | C | A | -0.109 | 0.017 | 4.73E-10 |
| SCZ | rs10803138 | 1 | 243555219 | CEP170, LOC731275, MIR4677, SDCCAG8 | A | G | -0.067 | 0.011 | 2.03E-09 |
| SCZ | rs14403 | 1 | 243663893 | AKT3, SDCCAG8 | C | T | 0.074 | 0.012 | 1.71E-10 |
| SCZ | rs2660304 | 1 | 98512127 | DPYD, DPYD-AS2, MIR137, MIR137HG, MIR2682 | G | T | -0.109 | 0.012 | 2.18E-18 |
| SCZ | rs6680011 | 1 | 95840866 | FLJ31662 | A | C | -0.074 | 0.013 | 2.83E-08 |
| SCZ | rs11165867 | 1 | 97878068 | DPYD, DPYD-AS1 | C | T | -0.069 | 0.013 | 3.87E-08 |
| SCZ | rs4144797 | 2 | 233562197 | C2orf82, GIGYF2, KCNJ13, NGEF | T | C | 0.082 | 0.01 | 4.33E-16 |
| SCZ | rs10196799 | 2 | 185640728 | ZNF804A | A | T | 0.056 | 0.01 | 4.51E-09 |
| SCZ | rs11685299 | 2 | 225391296 | CUL3 | C | A | 0.06 | 0.01 | 3.86E-09 |
| SCZ | rs56145559 | 2 | 73623439 | ALMS1 | C | T | -0.071 | 0.012 | 1.01E-09 |
| SCZ | rs2077586 | 2 | 73161551 | EMX1 | A | G | 0.061 | 0.011 | 2.96E-08 |
| SCZ | rs56807175 | 2 | 146436223 | / | T | TC | -0.088 | 0.013 | 1.36E-11 |
| SCZ | rs489939 | 3 | 161470592 | LOC101243545, OTOL1 | G | A | 0.057 | 0.01 | 1.24E-08 |
| SCZ | rs34796896 | 3 | 180623255 | DNAJC19, FXR1, LOC101928882, SOX2-OT | G | A | 0.083 | 0.012 | 3.19E-12 |
| SCZ | rs55672338 | 3 | 181051857 | SOX2-OT | T | A | -0.059 | 0.01 | 1.52E-09 |
| SCZ | rs6800435 | 3 | 10804551 | LINC00606, SLC6A11 | C | A | -0.082 | 0.015 | 2.00E-08 |
| SCZ | rs7632921 | 3 | 71543758 | FOXP1, MIR1284 | G | T | 0.056 | 0.01 | 9.52E-09 |
| SCZ | rs704373 | 3 | 63867355 | ATXN7, C3orf49, PSMD6, PSMD6-AS2, THOC7, THOC7-AS1 | A | G | 0.065 | 0.01 | 1.39E-10 |
| SCZ | rs62334820 | 4 | 176855221 | GPM6A | C | T | -0.081 | 0.012 | 9.60E-12 |
| SCZ | rs215411 | 4 | 23423603 | MIR548AJ2 | T | A | -0.058 | 0.01 | 1.40E-08 |
| SCZ | rs13169274 | 5 | 137855305 | ETF1 | T | C | -0.06 | 0.01 | 7.06E-10 |
| SCZ | rs16902086 | 5 | 45285752 | HCN1, LOC100506674, MRPS30 | A | G | -0.066 | 0.01 | 5.55E-11 |
| SCZ | rs16867576 | 5 | 88746331 | MEF2C-AS1 | A | G | 0.101 | 0.015 | 1.65E-11 |
| SCZ | rs217287 | 6 | 84407466 | ME1, PRSS35, SNAP91 | C | T | 0.07 | 0.01 | 9.53E-13 |
| SCZ | rs760608 | 6 | 114719447 | LOC107986638 | G | A | 0.061 | 0.011 | 1.90E-08 |
| SCZ | rs634940 | 6 | 93077500 | / | G | T | -0.063 | 0.011 | 1.30E-08 |
| SCZ | rs3130820 | 6 | 29206683 | xMHC | T | A | 0.248 | 0.018 | 2.12E-44 |
| SCZ | rs7789569 | 7 | 104927586 | KMT2E, KMT2E-AS1, LHFPL3, LHFPL3-AS2, LINC01004, SRPK2 | T | C | 0.065 | 0.01 | 7.00E-11 |
| SCZ | rs12705761 | 7 | 110976264 | IMMP2L, LRRN3 | G | C | 0.066 | 0.01 | 5.11E-11 |
| SCZ | rs211829 | 7 | 110048893 | LOC105375451 | T | C | 0.059 | 0.01 | 2.29E-09 |
| SCZ | rs7010876 | 8 | 89264751 | MMP16 | T | A | 0.062 | 0.011 | 6.51E-09 |
| SCZ | rs36043959 | 8 | 111472014 | / | G | A | 0.068 | 0.01 | 4.07E-12 |
| SCZ | rs67439964 | 8 | 143343398 | LINC00051, TSNARE1 | T | C | 0.072 | 0.012 | 1.16E-09 |
| SCZ | rs1473594 | 8 | 60696526 | CA8 | T | C | 0.064 | 0.01 | 3.33E-11 |
| SCZ | rs11993663 | 8 | 10032894 | MSRA | C | A | -0.056 | 0.01 | 3.40E-08 |
| SCZ | rs1042992 | 8 | 26269191 | BNIP3L, PPP2R2A | C | T | -0.074 | 0.012 | 3.67E-09 |
| SCZ | rs10156310 | 8 | 38209129 | NSD3 | A | T | 0.073 | 0.012 | 5.56E-10 |
| SCZ | rs55669358 | 8 | 34312412 | / | T | C | -0.095 | 0.017 | 1.37E-08 |
| SCZ | rs10985817 | 9 | 101071090 | GABBR2, MIR6854, TBC1D2 | T | C | -0.08 | 0.013 | 1.02E-09 |
| SCZ | rs1319017 | 9 | 84736303 | SPATA31D1, SPATA31D3, SPATA31D4, SPATA31D5P | G | A | -0.066 | 0.01 | 7.82E-11 |
| SCZ | rs12293670 | 11 | 124612932 | ESAM, MSANTD2, NRGN, VSIG2 | A | G | 0.081 | 0.01 | 1.70E-15 |
| SCZ | rs1765142 | 11 | 30378559 | ARL14EP, FSHB, MPPED2 | C | A | -0.058 | 0.01 | 1.13E-08 |
| SCZ | rs1899543 | 11 | 24406419 | LUZP2 | A | T | -0.058 | 0.009 | 1.23E-09 |
| SCZ | rs7951870 | 11 | 46373311 | DGKZ | T | C | -0.093 | 0.013 | 2.99E-13 |
| SCZ | rs58950470 | 11 | 65383755 | PCNX3 | G | T | -0.058 | 0.01 | 2.07E-08 |
| SCZ | rs4936215 | 11 | 133852684 | IGSF9B | A | G | 0.092 | 0.012 | 5.32E-14 |
| SCZ | rs893949 | 11 | 134296384 | / | C | T | 0.054 | 0.01 | 2.98E-08 |
| SCZ | rs2917569 | 11 | 132568255 | OPCML | T | C | 0.061 | 0.01 | 3.11E-10 |
| SCZ | rs4240748 | 12 | 92246786 | C12orf79 | C | G | -0.056 | 0.01 | 2.15E-08 |
| SCZ | rs1120004 | 12 | 23633432 | LOC101928441 | T | G | 0.062 | 0.011 | 1.42E-08 |
| SCZ | rs61937595 | 12 | 57682956 | INHBC, LRP1, MIR1228, NDUFA4L2, NXPH4, R3HDM2, SHMT2, STAC3 | C | T | 0.125 | 0.019 | 3.28E-11 |
| SCZ | rs36104021 | 12 | 103361112 | ASCL1 | CA | C | 0.07 | 0.012 | 7.31E-09 |
| SCZ | rs10783624 | 12 | 39522907 | ABCD2, KIF21A | C | A | 0.06 | 0.01 | 5.44E-09 |
| SCZ | rs9545047 | 13 | 79859456 | NDFIP2, NDFIP2-AS1, RBM26, RBM26-AS1 | A | C | 0.056 | 0.01 | 1.15E-08 |
| SCZ | rs10083370 | 14 | 104314182 | LINC00637, PPP1R13B | G | A | 0.075 | 0.01 | 3.44E-14 |
| SCZ | rs150437760 | 14 | 59981768 | CCDC175 | A | C | 0.121 | 0.022 | 4.58E-08 |
| SCZ | rs35604463 | 14 | 99712032 | BCL11B | G | A | 0.056 | 0.01 | 1.66E-08 |
| SCZ | rs10148671 | 14 | 29469373 | LINC02326 | T | C | -0.063 | 0.01 | 5.46E-10 |
| SCZ | rs1191551 | 14 | 30000405 | MIR548AI, PRKD1 | T | G | 0.073 | 0.012 | 4.12E-10 |
| SCZ | rs12148337 | 15 | 70589272 | LOC105370878 | G | T | -0.082 | 0.014 | 1.16E-08 |
| SCZ | rs3743078 | 15 | 78894759 | CHRNA3, CHRNA5, CHRNB4, HYKK, IREB2, PSMA4 | C | G | -0.078 | 0.011 | 3.11E-12 |
| SCZ | rs783540 | 15 | 83254708 | CPEB1 | A | G | -0.059 | 0.01 | 8.45E-10 |
| SCZ | rs12908161 | 15 | 85207825 | ADAMTSL3, ALPK3, DNM1P41, EFTUD1P1, GOLGA2P7, GOLGA6L4, GOLGA6L5P, LINC00933, LOC440300, LOC642423, LOC102724034, LOC103171574, NMB, SCAND2P, SEC11A, UBE2Q2L, UBE2Q2P1, WDR73, ZNF592, ZSCAN2 | A | G | 0.067 | 0.011 | 9.41E-10 |
| SCZ | rs12898315 | 15 | 61854003 | LOC107984782 | G | A | -0.057 | 0.01 | 2.51E-09 |
| SCZ | rs56282503 | 15 | 40566759 | ANKRD63, PAK6, PLCB2 | T | C | -0.062 | 0.011 | 2.30E-08 |
| SCZ | rs281299 | 15 | 47686081 | SEMA6D | C | T | -0.056 | 0.01 | 2.19E-08 |
| SCZ | rs17465671 | 16 | 63712719 | / | C | A | 0.057 | 0.01 | 4.14E-09 |
| SCZ | rs1975802 | 16 | 68285847 | PLA2G15 | A | G | -0.069 | 0.013 | 3.56E-08 |
| SCZ | rs7499750 | 16 | 13749265 | / | A | C | 0.07 | 0.011 | 4.24E-10 |
| SCZ | rs198160 | 16 | 24240725 | / | G | T | 0.056 | 0.01 | 4.88E-08 |
| SCZ | rs11646127 | 16 | 29966277 | ALDOA, C16orf92, DOC2A, FAM57B, GDPD3, HIRIP3, INO80E, KCTD13, MAPK3, PPP4C, TAOK2, TBX6, TMEM219, YPEL3 | G | C | 0.07 | 0.01 | 5.52E-13 |
| SCZ | rs12447542 | 16 | 7744180 | RBFOX1 | G | A | -0.086 | 0.015 | 1.44E-08 |
| SCZ | rs2161711 | 16 | 71359066 | CALB2, CHST4, CMTR2, HYDIN, ZNF19, ZNF23 | A | G | 0.07 | 0.013 | 4.22E-08 |
| SCZ | rs7216638 | 17 | 2156453 | LOC101927839, SMG6, SRR | T | A | 0.065 | 0.01 | 4.59E-10 |
| SCZ | rs7225476 | 17 | 78561603 | RPTOR | G | A | -0.052 | 0.01 | 4.86E-08 |
| SCZ | rs7508148 | 19 | 50152142 | BCL2L12, IRF3, NOSIP, PRMT1, PRR12, PRRG2, RRAS, SCAF1 | T | C | 0.077 | 0.012 | 4.06E-11 |
| SCZ | rs2905432 | 19 | 19484295 | ATP13A1, CILP2, GATAD2A, GMIP, HAPLN4, LPAR2, MAU2, NCAN, NDUFA13, PBX4, SUGP1, TM6SF2, TSSK6, YJEFN3, ZNF101 | G | A | 0.069 | 0.01 | 6.62E-12 |
| SCZ | rs2053079 | 19 | 30987423 | ZNF536 | A | G | -0.07 | 0.011 | 1.82E-10 |
| SCZ | rs6035706 | 20 | 20821005 | / | A | G | -0.06 | 0.01 | 7.24E-09 |
| SCZ | rs6002655 | 22 | 42603814 | TCF20 | C | T | -0.075 | 0.01 | 2.15E-14 |

Abbreviations: ADHD, attention-deficit/hyperactivity disorder; ASD, autism spectrum disorder; BD, bipolar disorder; Chr, chromosome; MDD, major depressive disorder; OCD, obsessive compulsive disorder; OR, odds ratio; PTSD, post-traumatic stress disorder; SCZ, schizophrenia; SE, standard error; SNP, single nucleotide polymorphism.

**Supplementary Table 6. The number of genetic variants used as instrumental variables for slee-related traits in then present study.**

| Risk factors | Independent SNPs* | SNPs associated with other traits | IVs | SNPs available in the outcome | | | | | | |
| --- | --- | --- | --- | --- | --- | --- | --- | --- | --- | --- |
|  |  |  |  | ADHD | ASD | BD | MDD | OCD | PTSD | SCZ |
| Chronotype | 268 | 40 | 228 | 220 | 221 | 228 | 228 | 227 | 228 | 228 |
| Insomnia | 184 | 34 | 150 | 144 | 149 | 150 | 150 | 150 | 150 | 150 |
| Sleep duration | 72 | 3 | 69 | 68 | 67 | 69 | 69 | 69 | 69 | 69 |

* Genetic variants for each exposure were selected based on the genome-wide significance (*P*<5×10^-8^) and clumping with an r^2^ threshold of 0.01 and a distance threshold of 250kb.

Abbreviations: ADHD, attention-deficit/hyperactivity disorder; ASD, autism spectrum disorder; BD, bipolar disorder; IVs, instrumental variable; MDD, major depressive disorder; PTSD, post-traumatic stress disorder; SCZ, schizophrenia.

**Supplementary Table 7. The number of genetic variants used as instrumental variables for psychiatric disorders in the present study.**

| Risk factors | Independent SNPs* | SNPs associated with other traits | IVs | SNPs available in the outcome | | |
| --- | --- | --- | --- | --- | --- | --- |
|  |  |  |  | Chronotype | Insomnia | Sleep duration |
| ADHD | 72 | 17 | 55 | 42 | 40 | 43 |
| ASD | 9 | 0 | 9 | 9 | 9 | 9 |
| BD | 22 | 4 | 18 | 15 | 14 | 15 |
| MDD | 36 | 24 | 12 | 11 | 11 | 11 |
| OCD | 15 | 2 | 13 | 12 | 12 | 12 |
| PTSD | 23 | 2 | 21 | 18 | 17 | 19 |
| SCZ | 123 | 38 | 85 | 70 | 68 | 70 |

* Genetic variants for each exposure were selected based on the genome-wide significance (*P*<5×10^-8^) and clumping with an r^2^ threshold of 0.01 and a distance threshold of 250kb.

Abbreviations: ADHD, attention-deficit/hyperactivity disorder; ASD, autism spectrum disorder; BD, bipolar disorder; IVs, instrumental variables; MDD, major depressive disorder; OCD, obsessive compulsive disorder; PTSD, post-traumatic stress disorder; SCZ, schizophrenia.

**Supplementary Table 8. Associations between genetically predicted insomnia and risk of psychiatric disorders**

| Risk factors | No. SNP | OR (95%CI) | *P* value for association | *P* value for MR-Egger intercept |
| --- | --- | --- | --- | --- |
| **ADHD** |  |  |  |  |
| IVW (random) | 144 | 1.33 (1.22-1.45) | 5.03E-11 |  |
| MR-Egger | 144 | 1.64 (1.04-2.58) | 0.034 | 0.359 |
| Weighted median | 144 | 1.27 (1.16-1.40) | 5.14E-07 |  |
| MR-PRESSO | 144 | 1.33 (1.22-1.45) | 8.73E-10 |  |
| Outlier corrected MR-PRESSO (3 outlier corrected) | 141 | 1.35 (1.24-1.45) | 9.23E-12 |  |
| Modified weights IVW | 144 | 1.33 (1.22-1.45) | 4.98E-11 |  |
| RAPS | 144 | 1.32 (1.24-1.40) | <2.0E-16 |  |
| **ASD** |  |  |  |  |
| IVW (random) | 149 | 1.12 (1.00-1.25) | 0.045 |  |
| MR-Egger | 149 | 1.98 (1.08-3.63) | 0.027 | 0.061 |
| Weighted median | 149 | 1.08 (0.92-1.26) | 0.337 |  |
| MR-PRESSO | 149 | 1.12 (1.00-1.25) | 0.046 |  |
| Modified weights IVW | 149 | 1.12 (1.00-1.25) | 0.045 |  |
| RAPS | 149 | 1.07 (0.96-1.20) | 0.219 |  |
| **BD** |  |  |  |  |
| IVW (random) | 150 | 1.12 (1.03-1.21) | 0.009 |  |
| MR-Egger | 150 | 1.41 (0.90-2.21) | 0.132 | 0.297 |
| Weighted median | 150 | 1.12 (1.01-1.23) | 0.025 |  |
| MR-PRESSO | 150 | 1.12 (1.03-1.21) | 0.010 |  |
| Outlier corrected MR-PRESSO (1 outlier corrected) | 149 | 1.11 (1.02-1.20) | 0.015 |  |
| Modified weights IVW | 150 | 1.12 (1.03-1.21) | 0.009 |  |
| RAPS | 150 | 1.11 (1.05-1.18) | 3.61E-04 |  |
| **MDD** |  |  |  |  |
| IVW (random) | 150 | 1.31 (1.25-1.37) | 6.88E-31 |  |
| MR-Egger | 150 | 0.64 (1.00-1.63) | 0.051 | 0.851 |
| Weighted median | 150 | 1.32 (1.25-1.39) | 5.13E-24 |  |
| MR-PRESSO | 150 | 1.31 (1.25-1.37) | 1.87E-22 |  |
| Outlier corrected MR-PRESSO (2 outlier corrected) | 148 | 1.30 (1.25-1.36) | 8.10E-24 |  |
| Modified weights IVW | 150 | 1.31 (1.25-1.37) | 7.36E-31 |  |
| RAPS | 150 | 1.32 (1.27-1.37) | <2.0E-16 |  |
| **OCD** |  |  |  |  |
| IVW (random) | 150 | 1.08 (0.93-1.25) | 0.332 |  |
| MR-Egger | 150 | 1.07 (0.48-2.39) | 0.864 | 0.994 |
| Weighted median | 150 | 1.12 (0.91-1.37) | 0.287 |  |
| MR-PRESSO | 150 | 1.08 (0.93-1.25) | 0.333 |  |
| Modified weights IVW | 150 | 1.08 (0.93-1.25) | 0.332 |  |
| RAPS | 150 | 1.08 (0.93-1.26) | 0.303 |  |
| **PTSD** |  |  |  |  |
| IVW (random) | 150 | 1.32 (1.23-1.40) | 1.42E-16 |  |
| MR-Egger | 150 | 1.27 (0.90-1.80) | 1.82E-01 | 0.840 |
| Weighted median | 150 | 1.37 (1.25-1.50) | 2.82E-11 |  |
| MR-PRESSO | 150 | 1.31 (1.23-1.40) | 7.16E-14 |  |
| Modified weights IVW | 150 | 1.31 (1.23-1.40) | 1.43E-16 |  |
| RAPS | 150 | 1.33 (1.25-1.42) | <2.0E-16 |  |
| **SCZ** |  |  |  |  |
| IVW (random) | 150 | 1.08 (0.99-1.18) | 0.094 |  |
| MR-Egger | 150 | 1.06 (0.66-1.71) | 0.813 | 0.941 |
| Weighted median | 150 | 1.07 (0.98-1.16) | 0.127 |  |
| MR-PRESSO | 150 | 1.08 (0.99-1.18) | 0.097 |  |
| Outlier corrected MR-PRESSO (9 outlier corrected) | 139 | 1.09 (1.01-1.18) | 0.022 |  |
| Modified weights IVW | 150 | 1.08 (0.99-1.18) | 0.094 |  |
| RAPS | 150 | 1.08 (1.03-1.13) | 0.003 |  |

Abbreviations: ADHD, attention-deficit/hyperactivity disorder; ASD, autism spectrum disorder; BD, bipolar disorder; CI, confidence interval; IVW, inverse-variance weighted; MDD, major depressive disorder; OR, odds ratio; OCD, obsessive compulsive disorder; PTSD, post-traumatic stress disorder; RAPS, Robust Adjusted Profile Score; SCZ, schizophrenia; SNP, single nucleoside polymorphism; MR-PRESSO test, MR Pleiotropy RESidual Sum and Outlier test.

**Supplementary Table 9. Mendelian randomization analyses of sleep-associated traits with major depressive disorder and post-traumatic stress disorder using datasets excluding the UK Biobank dataset.**

| Risk factors | No. SNP | OR (95%CI) | *P* value for association | *P* value for MR-Egger intercept |
| --- | --- | --- | --- | --- |
| **Chronotype** |  |  |  |  |
| **MDD** |  |  |  |  |
| IVW (random) | 59 | 0.89 (0.70-1.13) | 0.327 |  |
| MR-Egger | 59 | 0.63 (0.24-1.61) | 0.330 | 0.453 |
| Weighted median | 59 | 0.77 (0.56-1.07) | 0.124 |  |
| MR-PRESSO | 59 | 0.89 (0.70-1.13) | 0.331 |  |
| Modified weights IVW | 59 | 0.89 (0.70-1.13) | 0.327 |  |
| RAPS | 59 | 0.87 (0.69-1.10) | 0.249 |  |
| **PTSD** |  |  |  |  |
| IVW (random) | 222 | 1.01 (0.83-1.23) | 0.893 |  |
| MR-Egger | 222 | 1.01 (0.53-1.92) | 0.980 | 0.987 |
| Weighted median | 222 | 0.98 (0.73-1.31) | 0.881 |  |
| MR-PRESSO | 222 | 1.01 (0.83-1.23) | 0.893 |  |
| Modified weights IVW | 222 | 1.01 (0.83-1.23) | 0.893 |  |
| RAPS | 222 | 1.01 (0.82-1.24) | 0.913 |  |
| **Sleep duration** |  |  |  |  |
| **MDD** |  |  |  |  |
| IVW (random) | 15 | 1.00 (0.98-1.01) | 0.449 |  |
| MR-Egger | 15 | 0.91 (0.85-0.98) | 0.019 | 0.025 |
| Weighted median | 15 | 0.99 (0.98-1.01) | 0.405 |  |
| MR-PRESSO | 15 | 1.00 (0.98-1.01) | 0.442 |  |
| Modified weights IVW | 15 | 1.00 (0.98-1.01) | 0.429 |  |
| RAPS | 15 | 1.00 (0.98-1.01) | 0.496 |  |
| **PTSD** |  |  |  |  |
| IVW (random) | 69 | 1.00 (0.99-1.01) | 0.515 |  |
| MR-Egger | 69 | 0.98 (0.94-1.02) | 0.271 | 0.337 |
| Weighted median | 69 | 0.99 (0.98-1.01) | 0.448 |  |
| MR-PRESSO | 69 | 1.00 (0.99-1.01) | 0.494 |  |
| Modified weights IVW | 69 | 1.00 (0.99-1.01) | 0.491 |  |
| RAPS | 69 | 1.00 (0.99-1.01) | 0.465 |  |
| **Insomnia** |  |  |  |  |
| **MDD** |  |  |  |  |
| IVW (random) | 40 | 1.15 (0.97-1.36) | 0.127 |  |
| MR-Egger | 40 | 1.34 (0.57-3.14) | 0.508 | 0.719 |
| Weighted median | 40 | 1.18 (0.92-1.51) | 0.201 |  |
| MR-PRESSO | 40 | 1.15 (0.97-1.36) | 0.123 |  |
| Modified weights IVW | 40 | 1.15 (0.97-1.36) | 0.115 |  |
| RAPS | 40 | 1.16 (0.96-1.39) | 0.115 |  |
| **PTSD** |  |  |  |  |
| IVW (random) | 150 | 1.30 (1.08-1.56) | 0.005 |  |
| MR-Egger | 150 | 1.65 (0.62-4.41) | 0.320 | 0.630 |
| Weighted median | 150 | 1.31 (1.03-1.66) | 0.027 |  |
| MR-PRESSO | 150 | 1.30 (1.08-1.56) | 0.005 |  |
| Modified weights IVW | 150 | 1.30 (1.08-1.56) | 0.005 |  |
| RAPS | 150 | 1.31 (1.10-1.56) | 0.002 |  |

Abbreviations: CI, confidence interval; IVW, inverse-variance weighted; MDD, major depressive disorder; MR, Mendelian randomization; MR-PRESSO test, MR Pleiotropy RESidual Sum and Outlier test; OR, odds ratio; PTSD, post-traumatic stress disorder; RAPS, Robust Adjusted Profile Score.

**Supplementary Table 10. Associations between genetically predicted chronotype and risk of psychiatric disorders.**

| Risk factors | No. SNPs | OR (95%CI) | *P* value for association | *P* value for MR-Egger intercept |
| --- | --- | --- | --- | --- |
| **ADHD** |  |  |  |  |
| IVW (random) | 220 | 0.96 (0.88-1.04) | 0.324 |  |
| MR-Egger | 220 | 0.84 (0.66-1.08) | 0.180 | 0.286 |
| Weighted median | 220 | 0.96 (0.86-1.07) | 0.452 |  |
| MR-PRESSO | 220 | 0.96 (0.88-1.04) | 0.325 |  |
| Outlier corrected MR-PRESSO (1 outlier corrected) | 219 | 0.95 (0.87-1.03) | 0.229 |  |
| Modified weights IVW | 220 | 0.96 (0.88-1.04) | 0.324 |  |
| RAPS | 220 | 0.94 (0.87-1.00) | 0.062 |  |
| **ASD** |  |  |  |  |
| IVW (random) | 221 | 0.95 (0.82-1.09) | 0.466 |  |
| MR-Egger | 221 | 1.22 (0.75-1.99) | 0.419 | 0.287 |
| Weighted median | 221 | 1.04 (0.85-1.25) | 0.722 |  |
| MR-PRESSO | 221 | 0.95 (0.82-1.09) | 0.467 |  |
| Modified weights IVW | 221 | 0.95 (0.82-1.09) | 0.466 |  |
| RAPS | 221 | 0.97 (0.85-1.10) | 0.609 |  |
| **BD** |  |  |  |  |
| IVW (random) | 228 | 1.09 (0.99-1.20) | 0.068 |  |
| MR-Egger | 228 | 1.25 (0.95-1.64) | 0.113 | 0.314 |
| Weighted median | 228 | 1.14 (1.02-1.27) | 0.018 |  |
| MR-PRESSO global test | 228 | 1.09 (0.99-1.20) | 0.070 |  |
| Outlier corrected MR-PRESSO (6 outlier corrected) | 222 | 1.12 (1.02-1.22) | 0.015 |  |
| Modified weights IVW | 228 | 1.09 (0.99-1.20) | 0.068 |  |
| RAPS | 228 | 1.11 (1.04-1.19) | 0.002 |  |
| **MDD** |  |  |  |  |
| IVW (random) | 228 | 0.94 (0.89-0.98) | 0.009 |  |
| MR-Egger | 228 | 0.88 (0.76-1.01) | 0.078 | 0.362 |
| Weighted median | 228 | 0.90 (0.84-0.97) | 0.003 |  |
| MR-PRESSO | 228 | 0.94 (0.89-0.98) | 0.010 |  |
| Modified weights IVW | 228 | 0.94 (0.89-0.98) | 0.009 |  |
| RAPS | 228 | 0.94 (0.90-0.98) | 0.003 |  |
| **OCD** |  |  |  |  |
| IVW (random) | 227 | 0.99 (0.82-1.20) | 0.918 |  |
| MR-Egger | 227 | 1.57 (0.89-2.77) | 0.124 | 0.095 |
| Weighted median | 227 | 1.08 (0.83-1.41) | 0.572 |  |
| MR-PRESSO | 227 | 0.99 (0.82-1.20) | 0.918 |  |
| Outlier corrected MR-PRESSO (1 outlier corrected) | 226 | 1.01 (0.84-1.22) | 0.931 |  |
| Modified weights IVW | 227 | 0.99 (0.82-1.20) | 0.918 |  |
| RAPS | 227 | 1.01 (0.85-1.20) | 0.903 |  |
| **PTSD** |  |  |  |  |
| IVW (random) | 228 | 0.97 (0.90-1.05) | 0.520 |  |
| MR-Egger | 228 | 1.03 (0.82-1.28) | 0.820 | 0.626 |
| Weighted median | 228 | 0.99 (0.89-1.11) | 0.895 |  |
| MR-PRESSO | 228 | 0.97 (0.90-1.05) | 0.521 |  |
| Outlier corrected MR-PRESSO (2 outlier corrected) | 226 | 0.97 (0.90-1.04) | 0.403 |  |
| Modified weights IVW | 228 | 0.97 (0.90-1.05) | 0.520 |  |
| RAPS | 228 | 0.98 (0.91-1.06) | 0.591 |  |
| **SCZ** |  |  |  |  |
| IVW (random) | 228 | 0.88 (0.80-0.97) | 0.011 |  |
| MR-Egger | 228 | 1.04 (0.78-1.37) | 0.801 | 0.220 |
| Weighted median | 228 | 0.89 (0.81-0.99) | 0.031 |  |
| MR-PRESSO | 228 | 0.88 (0.80-0.97) | 0.012 |  |
| Outlier corrected MR-PRESSO (15 outlier corrected) | 213 | 0.89 (0.81-0.96) | 0.006 |  |
| Modified weights IVW | 228 | 0.88 (0.80-0.97) | 0.011 |  |
| RAPS | 228 | 0.89 (0.84-0.94) | 1.62E-05 |  |

Abbreviations: ADHD, attention-deficit/hyperactivity disorder; ASD, autism spectrum disorder; BD, bipolar disorder; CI, confidence interval; IVW, inverse-variance weighted; MDD, major depressive disorder; OR, odds ratio; OCD, obsessive compulsive disorder; PTSD, post-traumatic stress disorder; RAPS, Robust Adjusted Profile Score; SCZ, schizophrenia; SNP, single nucleoside polymorphism; MR-PRESSO test, MR Pleiotropy RESidual Sum and Outlier test.

**Supplementary Table 11. Associations between genetically predicted sleep duration and risk of psychiatric disorders.**

| Risk factors | No. SNPs | OR (95%CI) | *P* value for association | *P* value for MR-Egger intercept |
| --- | --- | --- | --- | --- |
| **ADHD** |  |  |  |  |
| IVW (random) | 68 | 1.00 (0.99-1.01) | 0.902 |  |
| MR-Egger | 68 | 1.00 (0.98-1.03) | 0.705 | 0.518 |
| Weighted median | 68 | 1.00 (0.99-1.00) | 0.311 |  |
| MR-PRESSO | 68 | 1.00 (0.99-1.01) | 0.903 |  |
| Outlier corrected MR-PRESSO (3 outlier corrected) | 65 | 1.00 (1.00-1.01) | 0.761 |  |
| Modified weights IVW | 68 | 1.00 (0.99-1.01) | 0.902 |  |
| RAPS | 68 | 1.00 (1.00-1.00) | 0.986 |  |
| **ASD** |  |  |  |  |
| IVW (random) | 67 | 1.00 (0.99-1.00) | 0.464 |  |
| MR-Egger | 67 | 1.02 (0.99-1.06) | 0.193 | 0.518 |
| Weighted median | 67 | 1.00 (0.99-1.01) | 0.837 |  |
| MR-PRESSO | 67 | 1.00 (0.99-1.01) | 0.467 |  |
| Modified weights IVW | 67 | 1.00 (0.99-1.01) | 0.464 |  |
| RAPS | 67 | 1.00 (0.99-1.00) | 0.250 |  |
| **BD** |  |  |  |  |
| IVW (random) | 69 | 1.01 (1.00-1.01) | 0.029 |  |
| MR-Egger | 69 | 1.00 (0.98-1.02) | 1.000 | 0.545 |
| Weighted median | 69 | 1.01 (1.00-1.01) | 0.026 |  |
| MR-PRESSO global test | 69 | 1.01 (1.00-1.01) | 0.033 |  |
| Outlier corrected MR-PRESSO (1 outlier corrected) | 68 | 1.01 (1.00-1.01) | 0.012 |  |
| Modified weights IVW | 69 | 1.01 (1.00-1.01) | 0.029 |  |
| RAPS | 69 | 1.01 (1.00-1.01) | 0.002 |  |
| **MDD** |  |  |  |  |
| IVW (random) | 69 | 1.00 (1.00-1.00) | 0.880 |  |
| MR-Egger | 69 | 1.00 (0.99-1.01) | 0.859 | 0.82 |
| Weighted median | 69 | 1.00 (1.00-1.00) | 0.786 |  |
| MR-PRESSO | 69 | 1.00 (1.00-1.00) | 0.881 |  |
| Modified weights IVW | 69 | 1.00 (1.00-1.00) | 0.880 |  |
| RAPS | 69 | 1.00 (1.00-1.00) | 0.144 |  |
| **OCD** |  |  |  |  |
| IVW (random) | 69 | 1.00 (0.99-1.01) | 0.576 |  |
| MR-Egger | 69 | 0.98 (0.94-1.01) | 0.166 | 0.201 |
| Weighted median | 69 | 1.00 (0.99-1.01) | 0.893 |  |
| MR-PRESSO | 69 | 1.00 (0.99-1.01) | 0.578 |  |
| Modified weights IVW | 69 | 1.00 (0.99-1.01) | 0.576 |  |
| RAPS | 69 | 1.00 (0.99-1.01) | 0.714 |  |
| **PTSD** |  |  |  |  |
| IVW (random) | 69 | 1.00 (0.99-1.00) | 0.471 |  |
| MR-Egger | 69 | 0.98 (0.97-1.00) | 0.037 | 0.05 |
| Weighted median | 69 | 1.00 (0.99-1.00) | 0.560 |  |
| MR-PRESSO | 69 | 1.00 (0.99-1.00) | 0.474 |  |
| Modified weights IVW | 69 | 1.00 (0.99-1.00) | 0.471 |  |
| RAPS | 69 | 1.00 (0.99-1.00) | 0.528 |  |
| **SCZ** |  |  |  |  |
| IVW (random) | 69 | 1.01 (1.00-1.01) | 0.074 |  |
| MR-Egger | 69 | 1.00 (0.98-1.03) | 0.800 | 0.817 |
| Weighted median | 69 | 1.01 (1.00-1.01) | 0.032 |  |
| MR-PRESSO | 69 | 1.01 (1.00-1.01) | 0.078 |  |
| Outlier corrected MR-PRESSO (15 outlier corrected) | 61 | 1.01 (1.00-1.01) | 0.074 |  |
| Modified weights IVW | 69 | 1.01(1.00-1.01) | 0.074 |  |
| RAPS | 69 | 1.01(1.00-1.01) | 4.05E-04 |  |

Abbreviations: ADHD, attention-deficit/hyperactivity disorder; ASD, autism spectrum disorder; BD, bipolar disorder; CI, confidence interval; IVW, inverse-variance weighted; MDD, major depressive disorder; OR, odds ratio; OCD, obsessive compulsive disorder; PTSD, post-traumatic stress disorder; RAPS, Robust Adjusted Profile Score; SCZ, schizophrenia; SNP, single nucleoside polymorphism; MR-PRESSO test, MR Pleiotropy RESidual Sum and Outlier test.

**Supplementary Table 12. Associations between genetically predicted psychiatric disorders and sleep-associated traits.**

| Risk factors | No. SNP | OR (95%CI) | *P* value for association | *P* value for MR-Egger intercept |
| --- | --- | --- | --- | --- |
| **Insomnia** |  |  |  |  |
| **ADHD** |  |  |  |  |
| IVW (random) | 40 | 1.03 (0.99-1.06) | 0.160 |  |
| MR-Egger | 40 | 0.99 (0.88-1.12) | 0.865 | 0.544 |
| Weighted median | 40 | 1.00 (0.96-1.04) | 1.000 |  |
| MR-PRESSO | 40 | 1.03 (0.99-1.06) | 0.168 |  |
| Outlier corrected MR-PRESSO (1 outlier corrected) | 39 | 1.02 (0.98-1.05) | 0.314 |  |
| Modified weights IVW | 40 | 1.03 (0.99-1.06) | 0.160 |  |
| RAPS | 40 | 1.01 (0.98-1.03) | 0.473 |  |
| **ASD** |  |  |  |  |
| IVW (random) | 9 | 0.99 (0.96-1.01) | 0.288 |  |
| MR-Egger | 9 | 0.94 (0.82-1.08) | 0.383 | 0.505 |
| Weighted median | 9 | 0.98 (0.94-1.02) | 0.283 |  |
| MR-PRESSO | 9 | 0.98 (0.96-1.01) | 0.319 |  |
| Modified weights IVW | 9 | 0.98 (0.96-1.01) | 0.288 |  |
| RAPS | 9 | 0.98 (0.95-1.01) | 0.240 |  |
| **BD** |  |  |  |  |
| IVW (random) | 14 | 1.04 (0.98-1.10) | 0.206 |  |
| MR-Egger | 14 | 1.04 (0.77-1.40) | 0.802 | 0.988 |
| Weighted median | 14 | 1.01 (0.95-1.08) | 0.683 |  |
| MR-PRESSO | 14 | 1.04 (0.98-1.10) | 0.228 |  |
| Modified weights IVW | 14 | 1.04 (0.98-1.10) | 0.206 |  |
| RAPS | 14 | 1.05 (1.01-1.10) | 0.027 |  |
| **MDD** |  |  |  |  |
| IVW (random) | 11 | 1.37 (1.15-1.65) | 0.001 |  |
| MR-Egger | 11 | 0.45 (0.14-1.50) | 0.195 | 0.067 |
| Weighted median | 11 | 1.40 (1.18-1.65) | 1.24E-04 |  |
| MR-PRESSO | 11 | 1.38 (1.15-1.65) | 0.006 |  |
| Outlier corrected MR-PRESSO (1 outlier corrected) | 10 | 1.45 (1.24-1.69) | 0.001 |  |
| Modified weights IVW | 11 | 1.37 (1.15-1.65) | 6.09E-04 |  |
| RAPS | 11 | 1.47 (1.31-1.64) | 2.16E-11 |  |
| **OCD** |  |  |  |  |
| IVW (random) | 12 | 1.00 (0.98-1.02) | 0.709 |  |
| MR-Egger | 12 | 1.04 (1.00-1.09) | 0.073 | 0.074 |
| Weighted median | 12 | 1.01 (0.99-1.03) | 0.443 |  |
| MR-PRESSO | 12 | 1.00 (0.98-1.02) | 0.716 |  |
| Modified weights IVW | 12 | 1.00 (0.98-1.02) | 0.709 |  |
| RAPS | 12 | 1.01 (0.99-1.02) | 0.566 |  |
| **PTSD** |  |  |  |  |
| IVW (random) | 17 | 1.06 (1.03-1.10) | 7.85E-04 |  |
| MR-Egger | 17 | 1.04 (0.96-1.13) | 0.313 | 0.603 |
| Weighted median | 17 | 1.07 (1.02-1.12) | 0.006 |  |
| MR-PRESSO | 17 | 1.06 (1.03-1.10) | 0.004 |  |
| Modified weights IVW | 17 | 1.06 (1.03-1.10) | 0.001 |  |
| RAPS | 17 | 1.07 (1.03-1.11) | 0.001 |  |
| **SCZ** |  |  |  |  |
| IVW (random) | 68 | 1.02 (0.99-1.05) | 0.120 |  |
| MR-Egger | 68 | 1.04 (0.90-1.21) | 0.568 | 0.763 |
| Weighted median | 68 | 1.01 (0.98-1.05) | 0.394 |  |
| MR-PRESSO | 68 | 1.02 (0.99-1.05) | 0.125 |  |
| Outlier corrected MR-PRESSO (1 outlier corrected) | 67 | 1.02 (1.00-1.05) | 0.058 |  |
| Modified weights IVW | 68 | 1.02 (0.99-1.05) | 0.120 |  |
| RAPS | 68 | 1.03 (1.00-1.05) | 0.016 |  |
| **Chronotype** |  |  |  |  |
| **ADHD** |  |  |  |  |
| IVW (random) | 42 | 1.00 (0.97-1.03) | 0.808 |  |
| MR-Egger | 42 | 1.04 (0.93-1.16) | 0.500 | 0.437 |
| Weighted median | 42 | 0.98 (0.95-1.01) | 0.205 |  |
| MR-PRESSO | 42 | 1.00 (0.97-1.03) | 0.809 |  |
| Outlier corrected MR-PRESSO (2 outlier corrected) | 39 | 0.99 (0.96-1.01) | 0.297 |  |
| Modified weights IVW | 42 | 1.00 (0.97-1.03) | 0.808 |  |
| RAPS | 42 | 0.99 (0.97-1.01) | 0.194 |  |
| **ASD** |  |  |  |  |
| IVW (random) | 9 | 0.99 (0.96-1.02) | 0.459 |  |
| MR-Egger | 9 | 1.08 (0.96-1.21) | 0.200 | 0.136 |
| Weighted median | 9 | 0.99 (0.95-1.02) | 0.370 |  |
| MR-PRESSO | 9 | 0.99 (0.96-1.02) | 0.480 |  |
| Modified weights IVW | 9 | 0.99 (0.96-1.02) | 0.459 |  |
| RAPS | 9 | 0.99 (0.96-1.01) | 0.335 |  |
| **BD** |  |  |  |  |
| IVW (random) | 15 | 0.99 (0.92-1.07) | 0.885 |  |
| MR-Egger | 15 | 1.05 (0.70-1.57) | 0.808 | 0.783 |
| Weighted median | 15 | 0.98 (0.92-1.04) | 0.442 |  |
| MR-PRESSO | 15 | 0.99 (0.92-1.07) | 0.887 |  |
| Outlier corrected MR-PRESSO (3 outlier corrected) | 12 | 1.01 (0.95-1.07) | 0.806 |  |
| Modified weights IVW | 15 | 0.99 (0.92-1.07) | 0.885 |  |
| RAPS | 15 | 0.98 (0.95-1.02) | 0.345 |  |
| **MDD** |  |  |  |  |
| IVW (random) | 11 | 1.01 (0.85-1.20) | 0.928 |  |
| MR-Egger | 11 | 1.28 (0.35-4.61) | 0.707 | 0.713 |
| Weighted median | 11 | 1.05 (0.91-1.21) | 0.521 |  |
| MR-PRESSO | 11 | 1.01 (0.85-1.20) | 0.930 |  |
| Outlier corrected MR-PRESSO (1 outlier corrected) | 10 | 1.07 (0.94-1.23) | 0.337 |  |
| Modified weights IVW | 11 | 1.01 (0.85-1.20) | 0.928 |  |
| RAPS | 11 | 1.06 (0.97-1.17) | 0.185 |  |
| **OCD** |  |  |  |  |
| IVW (random) | 12 | 1.01 (0.99-1.03) | 0.239 |  |
| MR-Egger | 12 | 0.96 (0.93-1.00) | 0.058 | 0.006 |
| Weighted median | 12 | 1.00 (0.98-1.02) | 0.960 |  |
| MR-PRESSO | 12 | 1.01 (0.99-1.03) | 0.263 |  |
| Modified weights IVW | 12 | 1.01 (0.99-1.03) | 0.238 |  |
| RAPS | 12 | 1.01 (0.99-1.02) | 0.408 |  |
| **PTSD** |  |  |  |  |
| IVW (random) | 18 | 1.00 (0.96-1.03) | 0.772 |  |
| MR-Egger | 18 | 0.96 (0.90-1.04) | 0.329 | 0.344 |
| Weighted median | 18 | 1.01 (0.97-1.05) | 0.802 |  |
| MR-PRESSO | 18 | 1.00 (0.96-1.03) | 0.776 |  |
| Modified weights IVW | 18 | 1.00 (0.96-1.03) | 0.772 |  |
| RAPS | 18 | 1.00 (0.97-1.03) | 0.891 |  |
| **SCZ** |  |  |  |  |
| IVW (random) | 70 | 0.97 (0.94-1.01) | 0.123 |  |
| MR-Egger | 70 | 0.95 (0.78-1.16) | 0.626 | 0.828 |
| Weighted median | 70 | 0.98 (0.95-1.01) | 0.119 |  |
| MR-PRESSO | 70 | 0.97 (0.94-1.01) | 0.128 |  |
| Outlier corrected MR-PRESSO (9 outlier corrected) | 61 | 1.03 (0.95-1.11) | 0.132 |  |
| Modified weights IVW | 70 | 0.97 (0.94-1.01) | 0.123 |  |
| RAPS | 70 | 0.99 (0.97-1.01) | 0.266 |  |
| **Sleep duration** |  |  |  |  |
| **ADHD** |  |  |  |  |
| IVW (random) | 43 | 0.98 (0.97-1.00) | 0.026 |  |
| MR-Egger | 43 | 0.98 (0.93-1.04) | 0.537 | 0.989 |
| Weighted median | 43 | 1.00 (0.98-1.01) | 0.610 |  |
| MR-PRESSO | 43 | 0.98 (0.97-1.00) | 0.031 |  |
| Outlier corrected MR-PRESSO (3 outlier corrected) | 40 | 0.99 (0.98-1.00) | 0.195 |  |
| Modified weights IVW | 43 | 0.98 (0.97-1.00) | 0.026 |  |
| RAPS | 43 | 0.99 (0.98-1.00) | 0.188 |  |
| **ASD** |  |  |  |  |
| IVW (random) | 9 | 1.01 (1.00-1.03) | 0.123 |  |
| MR-Egger | 9 | 1.05 (0.98-1.12) | 0.173 | 0.294 |
| Weighted median | 9 | 1.01 (0.99-1.02) | 0.492 |  |
| MR-PRESSO | 9 | 1.01 (1.00-1.03) | 0.161 |  |
| Modified weights IVW | 9 | 1.01 (1.00-1.03) | 0.123 |  |
| RAPS | 9 | 1.01 (1.00-1.02) | 0.080 |  |
| **BD** |  |  |  |  |
| IVW (random) | 15 | 1.00 (0.97-1.02) | 0.722 |  |
| MR-Egger | 15 | 0.92 (0.79-1.06) | 0.228 | 0.248 |
| Weighted median | 15 | 0.98 (0.96-1.01) | 0.173 |  |
| MR-PRESSO | 15 | 0.99 (0.97-1.02) | 0.727 |  |
| Outlier corrected MR-PRESSO (2 outlier corrected) | 13 | 0.98 (0.96-1.00) | 0.041 |  |
| Modified weights IVW | 15 | 0.99 (0.97-1.02) | 0.722 |  |
| RAPS | 15 | 0.98 (0.96-1.00) | 0.018 |  |
| **MDD** |  |  |  |  |
| IVW (random) | 11 | 0.92 (0.87-0.98) | 0.015 |  |
| MR-Egger | 11 | 0.64 (0.48-0.99) | 0.045 | 0.014 |
| Weighted median | 11 | 0.92 (0.87-0.98) | 0.011 |  |
| MR-PRESSO | 11 | 0.92 (0.87-0.98) | 0.035 |  |
| Outlier corrected MR-PRESSO (1 outlier corrected) | 10 | 0.90 (0.86-0.95) | 0.002 |  |
| Modified weights IVW | 11 | 0.92 (0.87-0.98) | 0.015 |  |
| RAPS | 11 | 0.91 (0.87-0.95) | 1.28E-04 |  |
| **OCD** |  |  |  |  |
| IVW (random) | 12 | 1.00 (0.99-1.01) | 0.935 |  |
| MR-Egger | 12 | 0.99 (0.97-1.01) | 0.441 | 0.418 |
| Weighted median | 12 | 1.00 (0.99-1.01) | 0.977 |  |
| MR-PRESSO | 12 | 1.00 (0.99-1.01) | 0.937 |  |
| Modified weights IVW | 12 | 1.00 (0.99-1.01) | 0.935 |  |
| RAPS | 12 | 1.00 (0.99-1.01) | 0.979 |  |
| **PTSD** |  |  |  |  |
| IVW (random) | 19 | 1.00 (0.97-1.02) | 0.831 |  |
| MR-Egger | 19 | 1.02 (0.97-1.08) | 0.397 | 0.296 |
| Weighted median | 19 | 0.99 (0.97-1.01) | 0.516 |  |
| MR-PRESSO | 19 | 1.00 (0.97-1.02) | 0.833 |  |
| Outlier corrected MR-PRESSO (2 outlier corrected) | 17 | 1.01 (1.00-1.02) | 0.156 |  |
| Modified weights IVW | 19 | 1.00 (0.97-1.02) | 0.831 |  |
| RAPS | 19 | 1.01 (0.99-1.02) | 0.267 |  |
| **SCZ** |  |  |  |  |
| IVW (random) | 70 | 1.02 (1.00-1.03) | 0.058 |  |
| MR-Egger | 70 | 1.08 (0.99-1.18) | 0.072 | 0.139 |
| Weighted median | 70 | 1.01 (1.00-1.03) | 0.089 |  |
| MR-PRESSO global test | 70 | 1.01 (1.00-1.03) | 0.062 |  |
| Outlier corrected MR-PRESSO (5 outlier corrected) | 65 | 1.02(1.00-1.03) | 0.022 |  |
| Modified weights IVW | 70 | 1.01 (1.00-1.03) | 0.058 |  |
| RAPS | 70 | 1.02 (1.01-1.03) | 0.001 |  |

Abbreviations: ADHD, attention-deficit/hyperactivity disorder; ASD, autism spectrum disorder; BD, bipolar disorder; CI, confidence interval; IVW, inverse-variance weighted; MDD, major depressive disorder; OR, odds ratio; OCD, obsessive compulsive disorder; PTSD, post-traumatic stress disorder; RAPS, Robust Adjusted Profile Score; SCZ, schizophrenia; SNP, single nucleoside polymorphism; MR-PRESSO test, MR Pleiotropy RESidual Sum and Outlier test.
